# Circulating metabolomic signatures of ultra-processed and minimally processed food intake and risk of cardiovascular disease and type 2 diabetes: 20-year follow-up in the Whitehall II cohort

**DOI:** 10.1186/s12916-026-05081-7

**Published:** 2026-07-21

**Authors:** Mengmei E. Wang, Annie Britton, Michail Katsoulis, Kiara Chang, Eszter P. Vamos, Clare H. Llewellyn

**Affiliations:** 1 Research Department of Epidemiology and Public Health, University College London, London, UK; 2 Public Health Policy Evaluation Unit, School of Public Health, Imperial College London, London, UK; 3 Unit for Lifelong Health and Ageing, University College London, London, UK; 4 Research Department of Behavioural Science and Health, University College London, London, UK

**Keywords:** Nutritional epidemiology, Cardiovascular disease, Type 2 diabetes, Ultra-processed foods, Minimally processed foods, Metabolic diseases, Metabolomics

## Abstract

**Background:**

Higher intakes of ultra-processed food (UPF) and minimally processed food (MPF) are linked to increased and decreased cardiometabolic risks, respectively, though mechanisms underlying these associations remain less understood. We aimed to identify metabolomic signatures associated with UPF and MPF intakes, examine their prospective associations with cardiovascular disease (CVD) and type 2 diabetes (T2D), and quantify their mediation of processing-disease associations.

**Methods:**

In the Whitehall II cohort, UPF and MPF intakes in 6010 participants were derived using the Nova classification from 127-item food frequency questionnaires administered at phases 3 and 5 (1991–1994, 1997–1999), averaged across phases. Fasting plasma samples were analysed for 233 metabolites using nuclear magnetic resonance (NMR) spectroscopy. Metabolomic signatures for UPF and MPF intake were derived using elastic net regression. Cox models examined associations between metabolomic signatures and incident CVD and T2D. Mediation analyses quantified the proportion explained by metabolomic signatures.

**Results:**

We identified metabolomic signatures for UPF (34 metabolites) and MPF (50 metabolites). The UPF signature correlated positively with pro-inflammatory and atherogenic metabolites and inversely with omega-3 fatty acids and high-density lipoproteins; the MPF signature showed the opposite pattern. 1094 CVD and 853 T2D events occurred over 20 and 18 years median follow-up, respectively. Comparing the highest to lowest tertile, higher UPF signature scores were associated with greater risk of (hazard ratio [HR] = 1.19; 95% confidence interval [CI] 1.02–1.39) and T2D (HR = 1.20; 95% CI 1.03–1.42), whereas higher MPF signature scores were inversely associated with these outcomes (CVD: HR = 0.81; 95% CI 0.69–0.94; T2D: HR = 0.60; 95% CI 0.48–0.73). Associations between the signatures and outcomes were robust to additional adjustment for UPF or MPF intake. The signatures mediated 42.8–51.1% of processing-disease associations (51.1% for UPF-CVD, 46.6% for MPF-CVD, 42.8% for UPF-T2D, and 47.8% for MPF-T2D; all p < 0.05).

**Conclusions:**

Metabolomic signatures of UPF and MPF intake showed opposing associations with CVD and T2D risk, partially mediating these processing–disease associations. These findings suggest biological pathways linking UPF intake to adverse cardiometabolic outcomes, though the unexplained proportion indicates additional mechanisms may contribute.

## Background

Cardiovascular disease (CVD) and type 2 diabetes (T2D) account for over 20 million deaths annually and affect more than one billion people worldwide [1]. Each poses a major public health and economic challenge, contributing significantly to healthcare costs and loss of productivity [1, 2]. These conditions frequently co-occur and share underlying pathophysiological mechanisms, including chronic inflammation, insulin resistance, and dyslipidemia [3]. While dietary factors represent key modifiable determinants of cardiometabolic risk, the rapid global dietary transition toward industrial food processing has coincided with rising disease incidence [4].

In particular, ultra-processed foods (UPF) have been in the spotlight in recent years. UPF, defined by the Nova classification, are industrial formulations made mostly or entirely from substances extracted or derived from foods, together with cosmetic additives, and typically contain little or no intact whole food [5]. UPF now constitutes 50–70% of energy intake in the UK, 14–44% across the rest of Europe and are rapidly expanding in low- and middle-income countries [6, 7]. An umbrella review reported that higher UPF consumption was associated with increased risks of T2D and CVD [8]. UPF tend to be more energy dense and higher in added sugars, saturated fat, and sodium than non-UPF, which may contribute to adverse cardiometabolic risk through established pathways including obesity, insulin resistance, and hypertension [8–13]. However, the review reported that the associations remained robust in most studies after adjustment for dietary composition and other potential confounders [14–16]. Processing-specific factors may explain these independent effects: techniques such as high-temperature treatment, chemical modification, and the use of novel food additives can disrupt metabolic regulation through pathways not captured by nutrient composition alone [13, 17, 18].

Despite growing epidemiological evidence, the molecular mechanisms linking UPF intake to cardiometabolic disease remain poorly understood. Observational studies have shown that associations between UPF intake and adverse health outcomes persist after adjustment for overall dietary quality and macronutrient composition, suggesting that health effects may not be fully explained by nutritional composition alone [19]. This gap highlights the critical need for mechanistic insights into how food processing influences human metabolism beyond traditional nutritional parameters.

Metabolomics profiling has emerged as a promising approach for investigating UPF-cardiometabolic health relationships, offering potential insights into the biological mechanisms linking high UPF intake with poorer health outcomes [20]. Recent controlled feeding trials have shown that diets composed of 80 versus 0% energy from UPF produced differences in plasma and urine metabolites [21], supporting the use of metabolomics to characterise UPF dietary patterns. Elucidating UPF-associated metabolomic signatures may help clarify the mechanisms through which food processing levels are associated with cardiometabolic health. Concurrently, growing interest in the health benefits of MPF warrants investigation of the potential favourable biological pathways, which remain insufficiently characterised.

To date, few studies have directly compared the metabolomic signatures of UPF and MPF intake. To our knowledge, only one study has analysed both in parallel [22], and most investigations have been constrained by narrow metabolite coverage, cross-sectional designs, or single time-point dietary assessments [23, 24]. Critically, no prospective study has investigated how metabolomic signatures of UPF and MPF relate to long-term cardiometabolic disease risk [22, 25]. This limits our ability to determine whether observed health risks are attributable to adverse metabolite profiles associated with UPF intake or to the absence of favourable metabolite profiles associated with MPF intake, or both.

We therefore leveraged data from a large, well-characterised population-based cohort with repeated dietary assessments and extended follow-up, to: (1) identify metabolomic signatures associated with UPF and MPF intake; (2) examine their prospective associations with incident CVD and T2D; and (3) quantify the proportion of processing-disease associations mediated by these metabolomic signatures.

## Methods

### Study population

Data were from the Whitehall II cohort study, established in 1985 using 10,308 British civil servants (67% men) aged 35–55 years recruited from 20 London-based government departments [26]. Follow-up clinical examinations have occurred approximately every five years, with the latest examination in 2019–2022 (phase 13). Participants provided written informed consent, and ethical approval was obtained from the University College London Hospital Committee on the Ethics of Human Research (reference number 85/0938).

In this study, we included participants who attended phase 5 clinical examinations (n = 8628) with dietary intake data from either phase 3 (1991–1994), phase 5 (1997–1999), or both. As metabolomics data were measured at phase 5 only, dietary intakes from phases 3 and 5 were averaged to capture cumulative dietary exposure and to establish temporal precedence of dietary exposure before metabolite measurement. The study timeline and data collection phases are illustrated in Additional file 1: Fig. S1. Exclusions included: missing dietary data at both phases (n = 1374), implausible energy intake (<500 or >3500 kcal for women; <800 or >4000 kcal for men; n = 470), death or loss to follow-up before phase 5 (n = 124), and non-participation in phase 5 examinations (n = 379), yielding 6281 participants with metabolite and dietary data. Participants with existing CVD (n = 170) or T2D (n = 101) at phase 5 (baseline) were excluded, yielding a final analytical sample of 6010 participants (Additional file 1: Fig. S2). We compared key baseline characteristics between participants included in the analytic sample and those excluded (Additional file 2: Table S1). Characteristics were broadly similar between groups.

### Measurement of metabolomics

Whitehall II utilised the Nightingale Health NMR (nuclear magnetic resonance) metabolomics platform (Helsinki, Finland) for the analysis of metabolites [27]. The metabolite panel comprised 233 metabolites measured by NMR spectroscopy, including: lipoprotein subclasses (very-low-density lipoprotein (VLDL), intermediate-density lipoprotein (IDL), low-density lipoprotein (LDL), and high-density lipoprotein (HDL)); cholesterol and lipid measures (total and free cholesterol, triglycerides, phospholipids); fatty acids (including omega-3, omega-6, and saturation ratios); apolipoproteins (A1 and B); glycolysis-related metabolites (glucose, lactate, pyruvate, citrate); amino acids; ketone bodies; fluid balance markers (albumin, creatinine); and inflammation-related markers (e.g., glycoprotein acetyls). The full list of 233 metabolites is provided in Additional file 2: Table S2. All metabolite concentrations were log-transformed and standardised to reduce skewness.

### Dietary assessment

Dietary intake was assessed at phase 3 and phase 5 using a validated 127-item semi-quantitative food frequency questionnaire (FFQ); detailed methodology has been reported elsewhere [28]. Two researchers independently classified each FFQ item according to Nova, with discrepancies resolved by consensus. Items potentially spanning multiple processing levels were assigned to the category corresponding to the most commonly consumed form in the UK [5]; where ambiguity remained, items were conservatively assigned to lower Nova categories, favouring attenuation of observed associations. The complete Nova classification of the Whitehall II FFQ has been published previously [12, 29].

Foods were also assigned to subcategories based on food type: UPF subcategories included savoury snacks, beverages, sauces, dairy products, UPF meats, sweets, cereals, breads and ready-to-eat meals; MPF subcategories comprised fresh fruits and vegetables, whole grains, legumes and beans, dairy products, meats and poultry, fish, and beverages (water, unsweetened tea and coffee), consistent with prior operationalisations in prospective cohort studies [30, 31]. UPF and MPF intakes were calculated as percentage of total daily food weight (grams) rather than energy, to capture non-nutritional processing factors, particularly in low- or zero-calorie ultra-processed products such as non-nutritively sweetened beverages [32]. Details of food item classification according to the Nova system, along with the categorisation of UPF and MPF subgroups, are provided in Additional file 2: Tables S3–S4.

### Covariates assessment

In line with prior literature, we adjusted for covariates measured at phase 5 (1997–1999), including sociodemographic characteristics, health behaviours, dietary indicators, and clinical measures, obtained via self-reported questionnaires and standardised clinical examinations [26].

Socio-demographic factors included age, sex, marital status (married/cohabiting vs. other), ethnicity (white vs. non-white), educational attainment (under secondary, secondary, or university level), and socioeconomic status (SES) based on civil service employment grade (low [clerical/support], intermediate [professional/executive], or high [administrative]).

Behavioural factors included smoking status (current, former, or never), physical activity levels (active [>2.5 h/week of moderate physical activity or >1 h/week of vigorous physical activity], inactive [<1 h/week of both moderate and vigorous physical activity], or moderately active [neither active nor inactive]), and alcohol intake (abstainers [none in the past week], moderate [1–14 units/week], or heavy [≥15 units/week], where 1 unit = 8 g alcohol) [33, 34].

Dietary factors comprised total energy intake (in kilocalories) as well as sodium (mg/day), total fat (g/day), sugar intake (g/day), fibre (g/day) and Alternative Healthy Eating Index-2010 (AHEI-2010).

Clinical factors were considered as follows: family history of CVD and T2D, body mass index (BMI) was calculated as weight in kilograms divided by height in metres squared (kg/m²) and modelled as a continuous variable. Height and weight were obtained in clinic by trained staff; full measurement details are reported elsewhere [35]. Hypertension was defined as either systolic blood pressure ≥140 mmHg, diastolic blood pressure ≥90 mmHg, or the use of antihypertensive medication. Dyslipidaemia was defined as meeting any one of the following criteria: total cholesterol >6.0 mmol/L; triglycerides >1.7 mmol/L; HDL-cholesterol <1.0 mmol/L; LDL-cholesterol >4.0 mmol/L; or receiving lipid-lowering medication [36].

### Outcome ascertainment

The primary outcomes were incident CVD and T2D. CVD cases were identified through Hospital Episode Statistics (HES) and mortality registers using International Classification of Diseases (ICD) 9/10 codes, with follow-up to October 2019. T2D cases were ascertained from clinical assessments and HES linkage (to March 2017) based on: (1) fasting glucose ≥7.0 mmol/L; (2) glucose-lowering medication use; (3) oral glucose tolerance test (OGTT) showing fasting glucose ≥7.0 mmol/L or 2-h glucose ≥11.1 mmol/L; (4) HbA1c ≥6.5%; or (5) self-reported or HES-recorded diabetes diagnosis. Participants meeting any criterion were classified as having T2D. Detailed outcome classifications are in Additional file 2: Table S5.

### Statistical analyses

#### Multiple imputation of missing covariates and UPF/MPF intake

Before performing inferential analyses, missing data for covariates, UPF/MPF intake, and metabolites were imputed using the random forest-based algorithm implemented in the R package ‘missForest’ (maximum 50 iterations), chosen for its ability to capture complex nonlinear relationships without parametric assumptions [37]. For participants with dietary data at both phases 3 and 5, average intakes were calculated to represent long-term exposure; when data were missing at one phase (8.6% at phase 3; 16.3% at phase 5), imputation enabled calculation of average intake. Among covariates, educational attainment had the highest proportion of missing data (24.3%). Imputed and complete data were compared in Additional file 2: Table S6.

#### Characteristics of the study participants

Baseline characteristics of the participants according to tertiles of average UPF and MPF intake from phases 3 (1991–1994) and 5 (1997–1999) were summarised. Continuous variables were summarised as medians (IQR) because several showed skewed, non-normal distributions.

#### Identification of UPF, MPF-related metabolites

Metabolites, UPF intake, and MPF intake were log-transformed to reduce skewness and subsequently standardised to z-scores (mean = 0, SD = 1) to enable comparison of effect sizes [38]. Associations between average UPF/MPF intake (phases 3 and 5) and plasma metabolites measured at phase 5 were examined using multivariable linear regression, adjusting for sociodemographic factors, lifestyle variables, total energy intake, dietary quality, and clinical measures (Additional file 2: Table S7–S8). Multiple testing was controlled using the Benjamini–Hochberg procedure to limit false-positive findings while maintaining power in this high-dimensional setting (false discovery rate [FDR]-adjusted p < 0.002).

Elastic net regression was then applied to the FDR-significant metabolites to derive the metabolomic signature, without covariate adjustment. Participants were randomly split into a 70% training set (n = 4207) and a 30% testing set (n = 1803); characteristics of the training and testing sets were similar (Additional file 2: Table S9). The same analytic pipeline was applied separately for UPF and MPF intake. The elastic net model involves two hyperparameters: α, which controls the balance between lasso (α = 1) and ridge (α = 0) penalties, and λ, which determines the overall strength of regularisation [39]. Both hyperparameters were tuned on the training set by 10-fold cross-validation: α was optimised using the ‘caret’ package, and λ was then selected as the value minimising cross-validation error (λ.min) using the ‘glmnet’ package. For the UPF signature, the final model selected α = 0.53 and λ = 0.24; for the MPF signature, α = 0.20 and λ = 0.36. The tuned model was refit in the full training set, and model robustness was subsequently assessed using leave-one-out cross-validation within the training set [40]. Metabolites with non-zero coefficients were retained, and the training-set coefficients were used as weights to compute the metabolomic signature score in the testing set (weighted sum).

Partial Spearman correlations (covariate-adjusted) were used to relate dietary intakes to metabolomic signature scores. To identify food contributors, covariate-adjusted partial Spearman correlations were calculated between signature metabolites and UPF or MPF food subgroups. Multiple testing was controlled using the more conservative Bonferroni correction, appropriate for this targeted follow-up analysis where minimising false-positive findings was prioritised over discovery, with statistical significance defined as P < 0.05 divided by the number of tests.

#### Survival analysis of UPF and MPF intake, metabolomic signatures, and cardiometabolic outcomes

Cox proportional hazards regression models assessed associations between UPF and MPF intake and incident CVD and T2D. Both dietary intakes and metabolomic signature scores were modelled as tertiles and as continuous z-standardised variables (per 1-SD increase). Metabolomic signature scores were calculated as the weighted sum of metabolite concentrations selected by elastic net regression, with weights corresponding to the elastic net coefficients.

Follow-up time was calculated from the date of the baseline assessment (phase 5) to the date of the first event (CVD or T2D), death, or the end of the follow-up period, whichever occurred first. Hazard ratios (HR) and 95% confidence intervals (CI) were estimated for each tertile, with tertile 1 (lowest intake) as the reference category. Proportional hazards assumptions were verified using Schoenfeld residuals tests; no violations across all models were detected (all p > 0.05).

Two sequential models were fitted. Model 1 adjusted for sociodemographic factors (sex, age, ethnicity, employment grade, marital status, education), lifestyle variables (physical activity, alcohol consumption, smoking status), total energy intake, clinical measures (BMI, dyslipidaemia, hypertension) and family history of CVD and T2D. Model 2 additionally adjusted for dietary quality indicators (sugar, sodium, fat and fibre intake) to examine whether associations were independent of nutrient composition. P for trend was calculated by treating the categorical variable as continuous using the median value within each category.

#### Mediation analysis

To quantify mediation, we conducted causal mediation analyses using the R package “mediation”. UPF or MPF intake was specified as the exposure, with incident CVD and T2D as the outcomes. We examined two sets of mediators: (i) the corresponding metabolomic signature score, and (ii) individual nutrient mediators (sugar, sodium, and saturated fat), each modelled separately. For each mediator, linear regression was used for the exposure–mediator relationship and we fitted parametric survival models for time-to-event (accelerated failure time models) including both the exposure and the mediator, adjusting for covariates as specified in Model 1. Dietary intake variables (total sugar, total fat, and sodium) were excluded from mediation models as they may lie on the causal pathway. The treatment and control values for the z-scored exposure were set at 1 and 0. Natural indirect effects (average causal mediation effects), natural direct effects, and total effects were estimated, with 95% CIs obtained via 1000 nonparametric bootstrap resamples. The proportion mediated was calculated from the indirect and total effects and reported only where the indirect effect was statistically significant. These analyses assume no unmeasured confounding of the exposure–outcome, exposure–mediator, and mediator–outcome relationships, and that no mediator–outcome confounder is affected by the exposure. The plausibility of these assumptions was considered in the interpretation of results [41].

#### Subgroup and sensitivity analyses

Stratified analyses were conducted according to sex (male, female), BMI (<25, 25–29.9, ≥30 kg/m²), and smoking status (current, former, and never smokers). Interactions between stratified factors and UPF/MPF intake or the metabolomic signature on the risk of CVD and T2D were tested. To explore which types of UPF drove the observed associations, nine UPF subgroups were each modelled separately as continuous variables (per 1-SD increment, %g/day) in relation to incident CVD and T2D.

Several sensitivity analyses were conducted to assess the robustness of findings. First, to assess sensitivity to the choice of imputation method, multiple imputation by chained equations (MICE) was performed, and Cox regression estimates from ten imputed datasets were pooled under Rubin’s rules [42]. Second, complete-case analyses were performed, restricting to participants with no missing data for exposures, covariates, or metabolites (N = 4551), to evaluate potential bias introduced by imputation. Third, lag analyses were conducted, excluding incident cases occurring within the first two years of follow-up, to minimise the potential for reverse causation whereby subclinical disease may have influenced dietary intake or metabolite levels. Lag analyses were applied to: (i) associations between UPF/MPF intake and incident CVD; (ii) associations between metabolomic signature scores and incident CVD; and (iii) corresponding models for incident T2D. Fourth, we adjusted for the Alternative Healthy Eating Index-2010 (AHEI-2010) in place of individual nutrient intake indicators, to examine whether overall diet quality, rather than specific nutrients, accounted for the observed associations. Fifth, to evaluate sensitivity to Nova classification decisions, two alternative reclassification scenarios were constructed for 35 FFQ items whose Nova assignment was potentially ambiguous: an upper bound scenario, in which ambiguous items were reclassified to maximise UPF intake, and a lower bound scenario, in which ambiguous items were reclassified to minimise UPF intake. Spearman correlations and Cox models were estimated under each scenario. Sixth, models examining the association between metabolomic signature scores and incident CVD and T2D were further adjusted for UPF or MPF intake, to assess whether the metabolomic signature captured information beyond self-reported dietary exposure. Seventh, the signatures were re-derived with confounders entered as unpenalised terms in the elastic net, and their correlation with the primary signatures assessed. Eighth, because NMR metabolomic profiling was undertaken at phase 5, analyses were repeated using phase 5 dietary intake alone, rather than averaging intake across phases 3 and 5, to assess whether the findings were robust when dietary exposure was temporally aligned with metabolomic assessment. Finally, to compare the relative discriminative performance of self-reported dietary intake and metabolomic signatures for incident CVD and T2D, Harrell’s C-index was estimated from Cox proportional hazards models with sequential addition of exposures to the base covariate set.

Analyses used R 4.4.1 and STATA 18.0 (StataCorp, College Station, TX); significance was set at *p* < 0.05 (two-sided), unless otherwise specified.

## Results

Baseline characteristics of the 6010 participants free of CVD and T2D at baseline are reported in Table 1. The majority were white (>90%), male (~77%), and married (~76%), with a median age of 55 years. Median overall UPF and MPF intakes were 18.3% (IQR: 14.0–23.5) and 62.1% (54.0–69.3) of g/day, respectively. Across increasing tertiles of UPF intake, median intake rose from 10.1% (10.2–12.3) to 17.5% (16.8–19.9) to 28.6% (23.5–30.5); corresponding medians for MPF intake were 48.0% (44.1–54.0), 62.2% (59.9–64.4), and 71.8% (69.3–75.5). Participants in the highest UPF tertile were more likely to be physically inactive (41.0 vs 32.1%) and to be current smokers (10.3 vs 8.2%), though heavy alcohol consumption was lower in this group (32.3 vs 37.2%). Individuals in the highest MPF tertile were less likely to be current smokers (8.1 vs 11.0%) or to engage in heavy alcohol intake (29.7 vs 36.6%).

**Table 1:** Baseline characteristics of study participants by tertiles of ultra-processed food (UPF) and minimally processed food (MPF) intake (N = 6010)

Variables	UPF intake			MPF intake		
	Tertile 1	Tertile 2	Tertile 3	Tertile 1	Tertile 2	Tertile 3
Overall Nova food intake (% g/day), median (IQR)^a^	18.3 (14.0–23.5)			62.1 (54.0–69.3)		
Nova food intake (% g/day) by tertiles, median (IQR)^a^	10.1 (10.2–12.3)	17.5 (16.8–19.9)	28.6 (23.5–30.5)	48.0 (44.1–54.0)	62.2 (59.9–64.4)	71.8 (69.3–75.5)
**Sex, n (%)**						
Male	1503 (75.0)	1544 (77.1)	1556 (77.6)	1589 (79.3)	1498 (74.8)	1516 (75.6)
Female	500 (25.0)	459 (22.9)	448 (22.4)	414 (20.7)	505 (25.2)	488 (24.4)
**Ethnicity, n (%)**						
White	1806 (90.2)	1821 (90.9)	1830 (91.3)	1827 (91.2)	1810 (90.4)	1820 (90.8)
Non-white	197 (9.8)	182 (9.1)	174 (8.7)	176 (8.8)	193 (9.6)	184 (9.2)
Age, median (IQR), years	54.9 (50.6–61.0)	55.1 (50.8–60.9)	55.5 (50.7–61.8)	55.2 (50.6–61.5)	55.1 (50.8–60.9)	55.3 (50.6–60.9)
**Family history, n (%)**						
**CVD**						
Yes	201 (10.0)	204 (10.2)	194 (9.7)	199 (9.9)	202 (10.1)	198 (9.9)
No	1802 (90.0)	1799 (89.8)	1810 (90.3)	1804 (90.2)	1801 (89.9)	1806 (90.1)
**T2D**						
Yes	159 (7.9)	175 (8.7)	170 (8.5)	126 (6.3)	156 (7.8)	222 (11.1)
No	1844 (92.1)	1828 (91.3)	1834 (91.5)	1877 (93.7)	1847 (92.2)	1782 (88.9)
**Marital status, n (%)**						
Yes	1510 (75.4)	1544 (77.1)	1520 (75.8)	1527 (76.2)	1527 (76.2)	1520 (75.8)
No	493 (24.6)	459 (22.9)	484 (24.2)	476 (23.8)	476 (23.8)	484 (24.2)
**Smoking, n (%)**						
Current smoker	164 (8.2)	182 (9.1)	206 (10.3)	220 (11.0)	170 (8.5)	162 (8.1)
Former smoker	779 (38.9)	783 (39.1)	686 (34.2)	757 (37.8)	741 (37.0)	750 (37.4)
Never smoker	1060 (52.9)	1038 (51.8)	1112 (55.5)	1026 (51.2)	1092 (54.5)	1092 (54.5)
**Alcohol consumption, n (%)**						
Abstainers	337 (16.8)	304 (15.2)	370 (18.5)	351 (17.5)	294 (14.7)	366 (18.3)
Moderate drinkers	921 (46.0)	977 (48.8)	986 (49.2)	919 (45.9)	922 (46.0)	1043 (52.0)
Heavy drinkers	745 (37.2)	722 (36.0)	648 (32.3)	733 (36.6)	787 (39.3)	595 (29.7)
**Education, n (%)**						
<Secondary	653 (32.6)	701 (35.0)	778 (38.8)	750 (37.4)	700 (34.9)	682 (34.0)
Secondary	538 (26.9)	491 (24.5)	472 (23.6)	480 (24.0)	510 (25.5)	511 (25.5)
University	812 (40.5)	811 (40.5)	754 (37.6)	773 (38.6)	793 (39.6)	811 (40.5)
**Physical activity, n (%)**						
Inactive	642 (32.1)	785 (39.2)	822 (41.0)	700 (34.9)	750 (37.4)	799 (39.9)
Active	899 (44.9)	915 (45.7)	880 (43.9)	920 (45.9)	900 (44.9)	874 (43.6)
Moderately active	462 (23.0)	303 (15.1)	302 (15.1)	383 (19.2)	353 (17.7)	331 (16.5)
**Employment grade, n (%)**						
Low	240 (12.0)	298 (14.9)	379 (18.9)	359 (17.9)	389 (19.4)	281 (14.0)
Medium	951 (47.5)	939 (46.9)	901 (45.0)	877 (43.8)	887 (44.3)	850 (42.4)
High	811 (40.5)	766 (38.2)	724 (36.1)	767 (38.3)	727 (36.3)	873 (43.6)
Body mass index, median (IQR), kg/m²	25.6 (23.6–27.8)	25.6 (23.5–28.1)	25.7 (23.6–28.3)	25.9 (23.8–27.9)	25.6 (23.6–27.8)	25.5 (23.4–27.9)
**Hypertension, n (%)**						
Yes	380 (19.0)	403 (20.1)	416 (20.8)	442 (22.1)	426 (21.3)	331 (16.5)
No	1623 (81.0)	1600 (79.9)	1588 (79.2)	1561 (77.9)	1577 (78.7)	1673 (83.5)
**Dyslipidaemia, n (%)**						
Yes	353 (17.6)	366 (18.3)	394 (19.7)	344 (17.2)	403 (20.1)	366 (18.3)
No	1650 (82.4)	1637 (81.7)	1610 (80.3)	1659 (82.8)	1600 (79.9)	1638 (81.7)
Total energy intake, median (IQR), kcal/day	1911 (1596–2270)	2181 (1791–2570)	2349 (1963–2787)	2322 (1890–2780)	2229 (1823–2571)	2000 (1611–2332)
Total sodium intake, median (IQR), mg/day	2194 (1719–2711)	2624 (2115–3253)	2963 (2378–3611)	2815 (2200–3511)	2647 (2126–3309)	2324 (1788–2929)
Total sugar intake, median (IQR), g/day	117 (93–148)	130 (101–161)	138 (108–171)	125 (97.2–161.8)	130 (102–163)	129 (100–160)
Total saturated fat intake, median (IQR), g/day	24.3 (11.0–34.9)	30.3 (21.5–41.7)	34.4 (25.2–46.7)	33.2 (23.0–45.3)	29.8 (22.0–37.8)	25.3 (18.6–33.6)
Total fat intake, median (IQR), g/day	64.0 (49.4–82.0)	75.9 (58.4–94.8)	84.6 (66.1–106.9)	81.0 (63.2–104.5)	75.6 (58.6–94.8)	66.5 (51.0–85.8)
Total Fibre intake, median (IQR), g/day	25.7 (20.1–33.1)	25.4 (19.8–30.5)	23.5 (18.1–30.1)	24.3 (18.5–31.2)	25.6 (21.8–32.3)	26.1 (22.9–33.5)
AHEI-2010 index	54.0 (46.5–60.5)	53.5 (46.5–60.0)	53.0 (47.0–60.5)	53.5 (46.5–60.5)	53.6 (46.7–60.4)	53.7 (47.0–61.5)

Abbreviations: *UPF* ultra-processed foods, *MPF* minimally processed foods, *IQR* interquartile range, *SD* standard deviation, *CVD* cardiovascular disease, *T2D* type 2 diabetes, *AHEI* alternative healthy eating index

^a^Continuous variables are expressed as median (interquartile range) or mean (standard deviation); categorical variables are expressed as n (%). Overall UPF and MPF intake are reported for the whole sample to contextualise the 1-SD associations reported in downstream analyses.

Nutrient profiles differed substantially across tertiles. Higher UPF intake was associated with greater intakes of energy (1911 to 2349 kcal/day), sodium (2194 to 2963 mg/day), total sugar (117 to 138 g/day), saturated fat (24.3 to 34.4 g/day), and total fat (64.0 to 84.6 g/day), alongside a modest decrease in total fibre (25.7 to 23.5 g/day). Higher MPF intake showed the opposite pattern, with lower intakes of energy, sodium, sugar, saturated fat, and total fat, and higher total fibre (24.3 to 26.1 g/day) across increasing tertiles.

### Identification of metabolites for UPF and MPF metabolomic signature scores

**Fig. 1:**
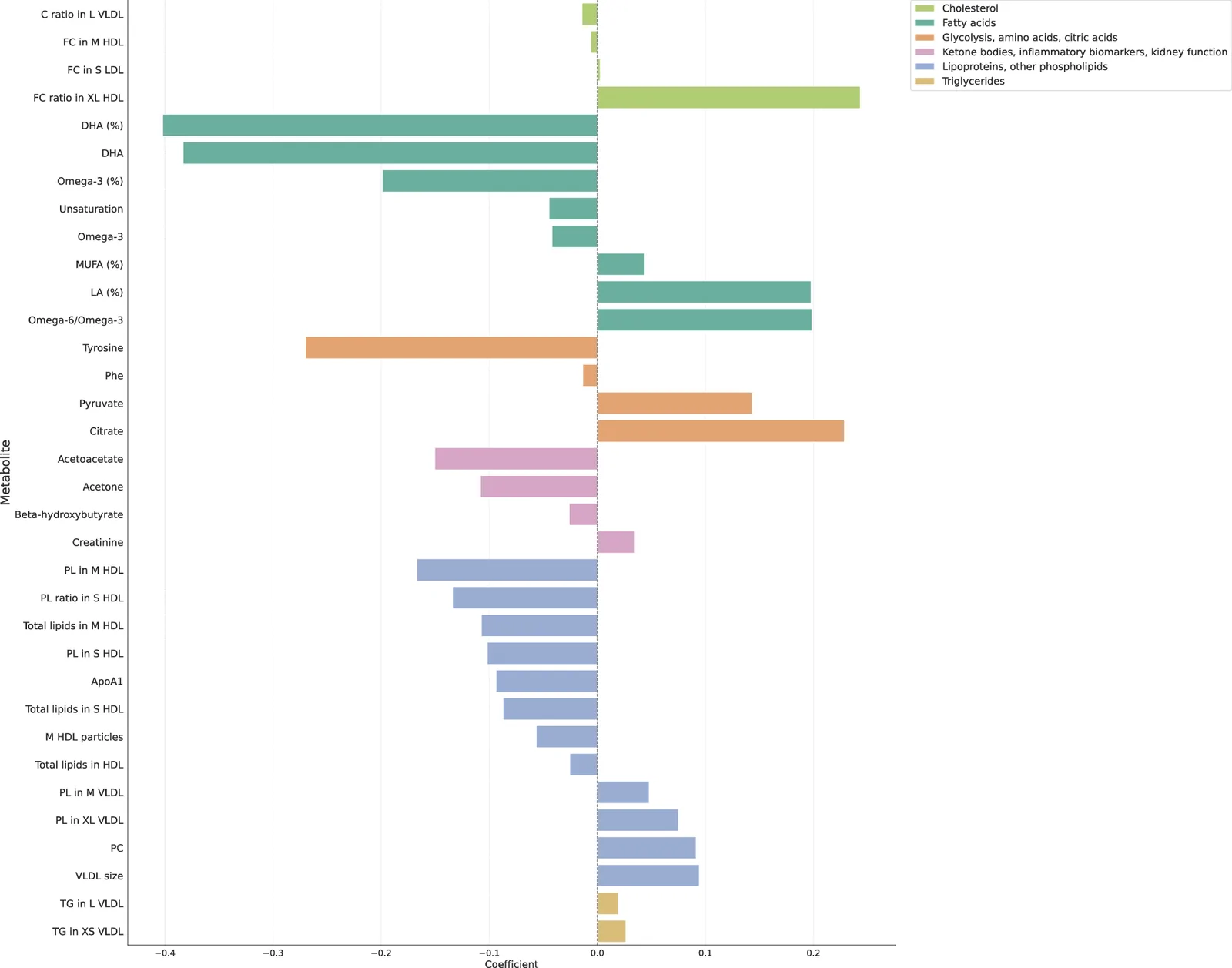
The coefficients of 34 metabolites with the strongest influence on the component in the UPF metabolomic signature. Legend: Bars show the non-zero coefficients for metabolites retained in the final elastic-net model used to derive the UPF signature score. Positive coefficients indicate biomarkers contributing to a higher UPF signature score (higher levels with higher UPF intake), whereas negative coefficients indicate inverse contributions. Colours denote metabolite types. Abbreviations: C cholesterol, FC free cholesterol, PL phospholipids, TG triglycerides, PC phosphatidylcholines, ApoA1 apolipoprotein A-I, VLDL very-low-density lipoprotein, LDL low-density lipoprotein, HDL high-density lipoprotein, XL very large, L large, M medium, S small, XS very small, DHA docosahexaenoic acid, LA linoleic acid, MUFA monounsaturated fatty acids, Omega-3 omega-3 fatty acids, Omega-6/Omega-3 ratio of omega-6 to omega-3 fatty acids, Unsaturation degree of unsaturation, Phe phenylalanine. “VLDL size” denotes mean VLDL particle diameter; “ratio in” a subclass denotes the ratio of that component to total lipids within the subclass; “(%)” denotes the metabolite expressed as a percentage of total fatty acids

**Fig. 2:**
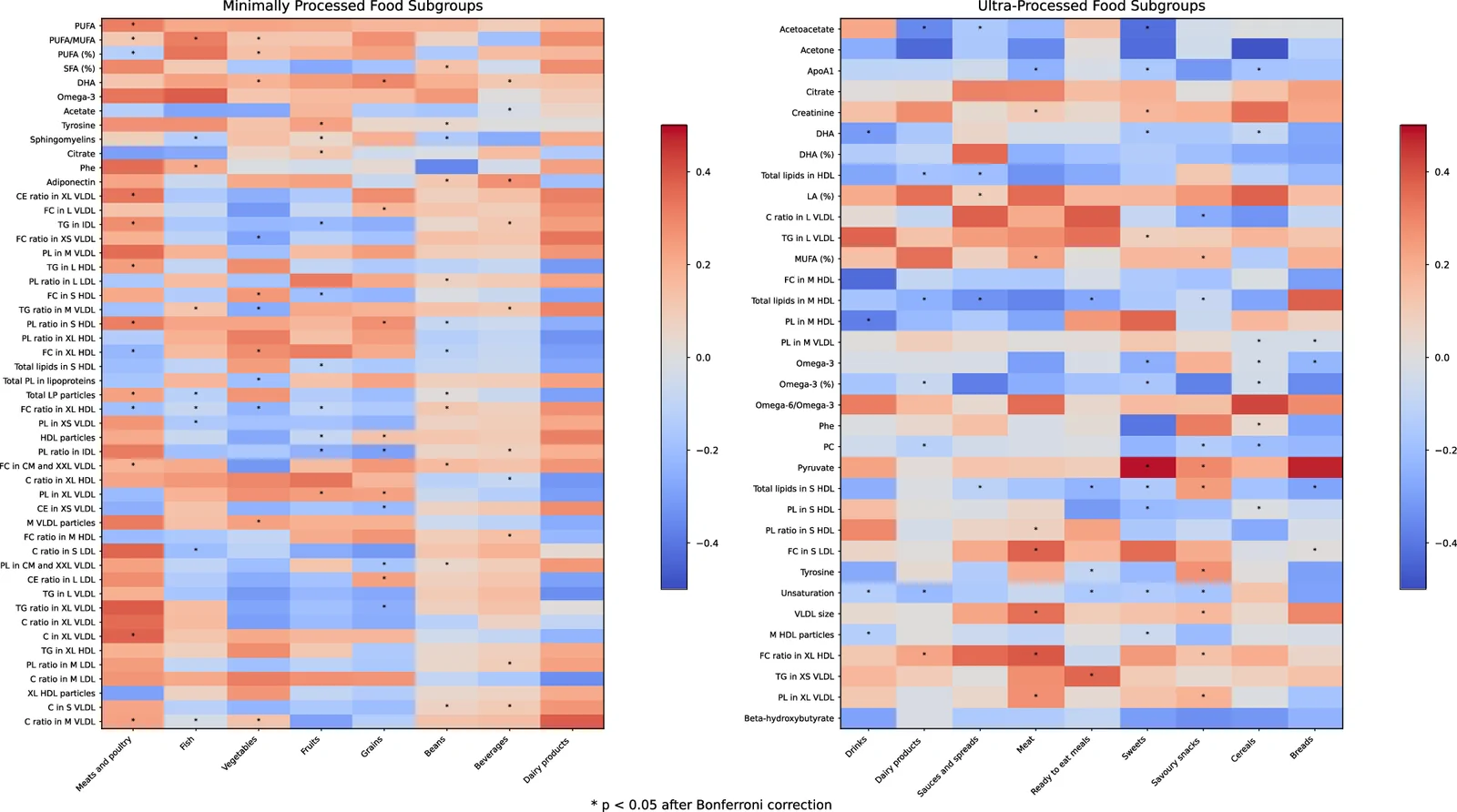
Correlation Between Food Subgroup Intake and Circulating Metabolites for Minimally and Ultra-Processed Foods. Legend: Heatmaps display correlation coefficients (r) between intakes of MPF subgroups (left panel) and UPF subgroups (right panel) and metabolomic measures (rows). Warmer colours indicate positive correlations, and cooler colours indicate negative correlations, with colour intensity proportional to the magnitude of r (scale shown). Asterisks denote correlations with p < 0.05 after Bonferroni correction. Abbreviations: MPF minimally processed food, UPF ultra-processed food, C cholesterol, FC free cholesterol, CE cholesteryl esters, PL phospholipids, TG triglycerides, PC phosphatidylcholines, ApoA1 apolipoprotein A-I, LP lipoprotein, VLDL very-low-density lipoprotein, IDL intermediate-density lipoprotein, LDL low-density lipoprotein, HDL high-density lipoprotein, CM chylomicrons, XXL extremely large, XL very large, L large, M medium, S small, XS very small, PUFA polyunsaturated fatty acids, MUFA monounsaturated fatty acids, SFA saturated fatty acids, DHA docosahexaenoic acid, LA linoleic acid, Omega-3 omega-3 fatty acids, Omega-6/Omega-3 ratio of omega-6 to omega-3 fatty acids, Unsaturation degree of unsaturation, Phe phenylalanine. “VLDL size” denotes mean VLDL particle diameter; “ratio in” a subclass denotes the ratio of that component to total lipids within the subclass; “(%)” denotes the metabolite expressed as a percentage of total fatty acids

Of the 233 metabolites analysed, 120 were significantly associated with UPF and 122 with MPF after correction for multiple testing (FDR-adjusted p < 0.002; Additional file 2: Tables S7–S8). The metabolomic signature scores based on selected metabolites were significantly correlated with UPF intake (Pearson r = 0.34, p value < 0.001) or MPF intakes (Pearson r = 0.32, p value < 0.001) (Additional file 1: Fig. S3). The UPF and MPF metabolomic signature scores were weakly and inversely correlated (Spearman r = –0.04, p = 0.0018). Among UPF-related metabolites, 34 were selected by the elastic net model (non-zero coefficients at the optimal penalty) and were used to construct the UPF metabolomic signature score as a weighted sum of these metabolites. Of the selected metabolites, 20 exhibited negative coefficient values in the UPF metabolomic signature score (Fig. 1). The metabolites most strongly and inversely associated with UPF intake included docosahexaenoic acid (DHA), omega-3 fatty acids, the ratio of DHA to total fatty acids, omega-3 fatty acids to total fatty acids, phospholipids in medium HDL particles, and acetoacetate. Conversely, 14 metabolites had positive coefficients, for which the strongest coefficients were for free cholesterol-to-total lipid ratio in very large HDL particles, citrate, omega-6 to omega-3 fatty acids, pyruvate, phospholipids in very large VLDL particles, and triglycerides in very small VLDL particles. The positive correlations with the above UPF-related metabolites were also observed for certain UPF subgroups, including ultra-processed meats, drinks, savoury snacks, and sweets (Fig. 2).

**Fig. 3:**
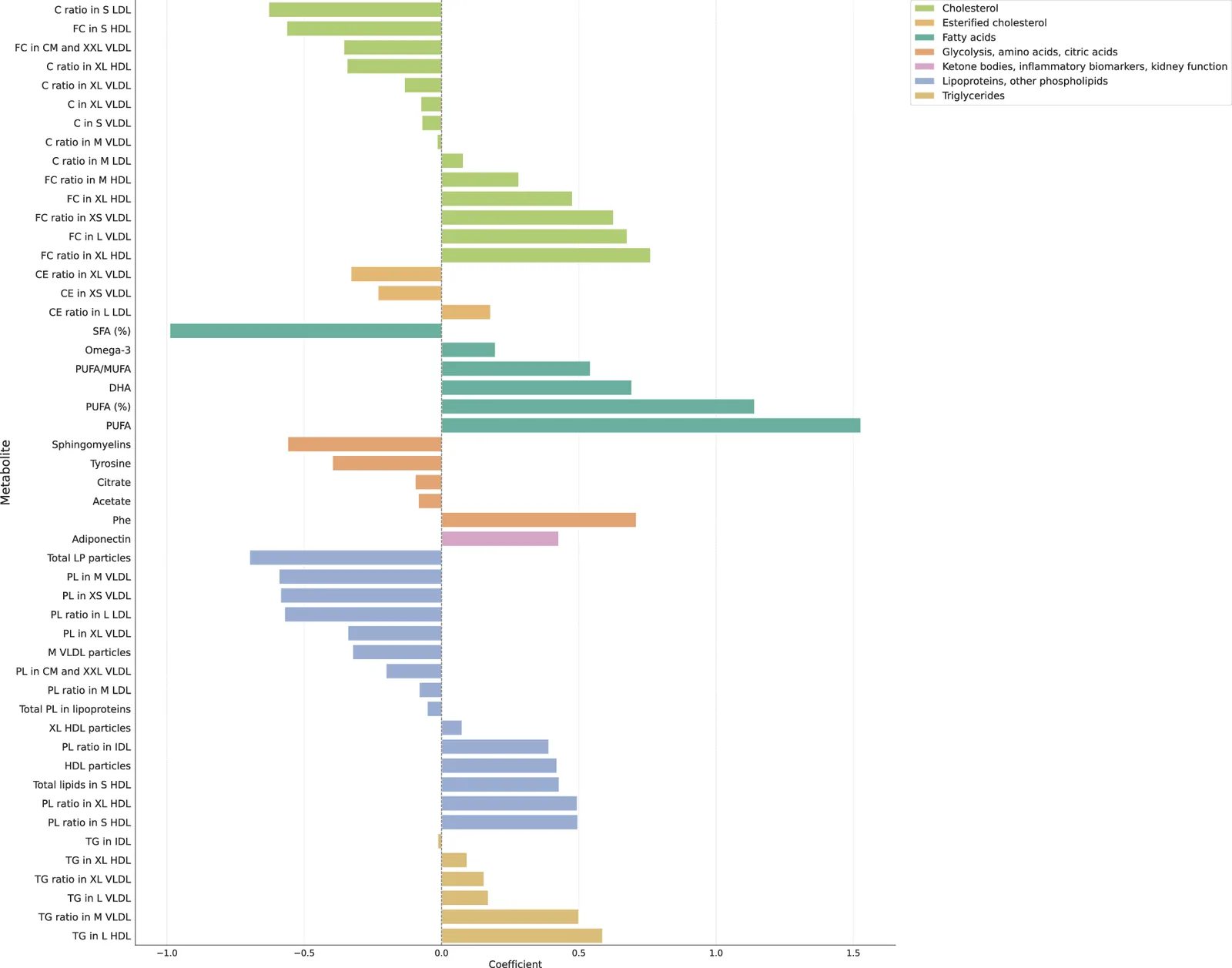
The coefficients of 50 metabolites with the strongest influence on the component in the MPF metabolomic signature. Legend: Bars show the non-zero coefficients for the metabolites retained in the final elastic-net model used to derive the MPF signature score. Positive coefficients indicate biomarkers contributing to a higher MPF signature score (i.e. higher circulating levels with higher MPF intake), whereas negative coefficients indicate inverse contributions. Bar colours denote metabolite class. Abbreviations: C cholesterol, FC free cholesterol, CE cholesteryl esters, PL phospholipids, TG triglycerides, LP lipoprotein, VLDL very-low-density lipoprotein, IDL intermediate-density lipoprotein, LDL low-density lipoprotein, HDL high-density lipoprotein, CM chylomicrons, XXL extremely large, XL very large, L large, M medium, S small, XS very small, PUFA polyunsaturated fatty acids, MUFA monounsaturated fatty acids, SFA saturated fatty acids, DHA docosahexaenoic acid, Omega-3 omega-3 fatty acids, Phe phenylalanine. “ratio in” a subclass denotes the ratio of that component to total lipids within the subclass; “(%)” denotes the metabolite expressed as a percentage of total fatty acids

Using a leave-one-out elastic-net model for MPF intake, 50 of 122 metabolites contributed to the MPF metabolomic score, with 25 positive coefficients. Strongest positive contributors included PUFA, the PUFA-to-total fatty acids ratio, DHA, free cholesterol-to-total lipids in very large HDL, and phenylalanine (Fig. 3). The strongest negative coefficients were for saturated-to-total fatty acids ratio, total lipoprotein particles, cholesterol-to-total lipids in small LDL, and phospholipids in very small VLDL.

Eleven metabolites contributed to both UPF and MPF signature scores (Additional file 1: Fig. S4). Three were concordant: the free cholesterol to total lipids ratio in very large HDL, triglycerides in large VLDL were positive in both signatures, whereas tyrosine was negative in both. The remaining eight were discordant: DHA, omega-3 fatty acids, phenylalanine, total lipids in small HDL, and phospholipids to total lipids ratio in small HDL were positive for MPF but negative for UPF, whereas citrate, phospholipids in medium VLDL, and phospholipids in very large VLDL showed the reverse pattern.

Pairwise correlations showed distinct metabolite-food subgroup patterns, displayed as heatmaps (Fig. 2). Among MPF subgroups, fish drove the strongest omega-3, DHA, and PUFA correlations, grains and beans contributed most to tyrosine associations, and vegetables to sphingomyelins and citrate. All MPF subgroups generally correlated positively with HDL lipids and negatively with VLDL triglycerides, with fish showing the largest effect sizes. However, minimally processed meats and poultry showed a more mixed profile, with some positive VLDL associations. Among UPF subgroups, meats, sweets, and ready-to-eat meals showed the strongest and most consistent adverse correlations, including positive associations with pyruvate, creatinine, VLDL triglycerides, and MUFA, and negative associations with DHA, omega-3 fatty acids, and HDL measures. However, there was notable heterogeneity across UPF subtypes: pyruvate correlated most strongly with ultra-processed meats, breads, and sweets, but weakly with drinks and cereals; creatinine was most prominent for meats and ready-to-eat meals; and drinks showed a distinctive profile with significant inverse associations for acetoacetate. Savoury snacks and cereals showed comparatively muted signals across most metabolites.

### Association between the metabolomic signatures score of UPF and MPF intake and the risk of CVD

Among 6010 participants, over a median follow-up of 20 years, totalling 120,200 person-years, 1094 incident CVD events were documented. Table 2 presents the associations of UPF and MPF intake and their corresponding metabolomic signatures with incident CVD.

**Table 2:** Prospective associations of tertiles of UPF and MPF intake and metabolic signature scores with incident cardiovascular disease in the Whitehall II cohort: multivariable Cox regression models (N = 6010; 1094 CVD cases)

Exposure	Tertile 1 (low)	Tertile 2	Tertile 3 (high)	P trend	Per 1 SD increase
***n***	2003	2003	2004		6010
**UPF intake**					
Events	324	361	409		1094
Model 1	Reference	1.15 (0.99–1.33)	1.26 (1.08–1.48)	0.004	1.19 (1.04–1.37)
Model 2	Reference	1.13 (0.98–1.31)	1.23 (1.05–1.44)	0.012	1.17 (1.03–1.35)
**MPF intake**					
Events	376	352	366		1094
Model 1	Reference	0.89 (0.83–1.03)	0.85 (0.71–0.99)	0.036	0.86 (0.78–0.93)
Model 2	Reference	0.91 (0.86–1.05)	0.82 (0.72–0.97)	0.035	0.87 (0.79–0.95)
**UPF metabolic signature**					
Events	333	361	400		1094
Model 1	Reference	1.09 (0.94–1.26)	1.20 (1.03–1.39)	0.021	1.16 (1.03–1.32)
Model 2	Reference	1.07 (0.92–1.24)	1.19 (1.02–1.39)	0.032	1.14 (1.02–1.31)
**MPF metabolic signature**					
Events	413	390	291		1094
Model 1	Reference	0.92 (0.80–1.06)	0.78 (0.67–0.90)	0.003	0.84 (0.79–0.93)
Model 2	Reference	0.94 (0.81–1.08)	0.81 (0.69–0.94)	0.01	0.86 (0.80–0.94)

Model 1: Adjusted for age at baseline, sex, ethnicity, education, employment grade, marital status, physical activity, alcohol consumption, smoking status, family history of CVD, total energy intake, BMI, hypertension, and dyslipidaemia

Model 2: Model 1 additionally adjusted for indicators of diet quality (total sugar, sodium, total fat, and fibre)

P-trend was calculated by treating the categorical variable as continuous using the median value within each category. Per 1 SD estimates represent the HR associated with each standard deviation increment in the exposure, modelled as a continuous variable

Abbreviations: *CI* confidence interval, *HR* hazard ratio, *MPF* minimally processed foods, *SD* standard deviation, *UPF* ultra-processed foods

Higher UPF intake was associated with increased CVD risk in a dose-response manner (Table 2), with 29.6, 33.0, and 37.4% of cases occurring across increasing tertiles. Participants in the highest tertile exhibited a 26% elevated risk compared to the lowest tertile (HR 1.26, 95% CI 1.08–1.48, p-trend = 0.004) in Model 1. This association slightly attenuated but remained significant after additional adjustment for diet quality indicators (HR 1.23, 95% CI 1.05–1.44, p-trend = 0.012). Per 1-SD increment in UPF intake, the fully adjusted HR was 1.17 (95% CI 1.03–1.35). Conversely, higher MPF intake was inversely associated with CVD risk, with participants in the highest tertile having an 18% lower risk compared to the lowest tertile in the fully adjusted model (HR 0.82, 95% CI 0.72–0.97, p-trend = 0.035). Each 1-SD increment in MPF intake was associated with a HR of 0.87 (95% CI 0.79–0.95).

The UPF-associated metabolomic signature showed a similar positive dose-response relationship with CVD incidence, with 30.4, 33.0, and 36.6% of cases across increasing tertiles. The highest tertile conferred a 20% increased risk in Model 1 (HR 1.20, 95% CI 1.03–1.39, p-trend = 0.021) and 19% increased risk in Model 2 (HR 1.19, 95% CI 1.02–1.39, p-trend = 0.032), with a per-1-SD HR of 1.14 (95% CI 1.02–1.31) in the fully adjusted model. Conversely, the MPF-associated metabolomic signature was inversely associated with CVD risk, with 37.8, 35.6, and 26.6% of cases across increasing tertiles. The MPF metabolomic signature showed a slightly stronger inverse association with CVD risk than dietary MPF intake alone, with a 22% lower risk in the highest tertile in Model 1 (HR 0.78, 95% CI 0.67–0.90, p-trend = 0.003) and 19% in Model 2 (HR 0.81, 95% CI 0.69–0.94, p-trend = 0.01), corresponding to a per-1-SD HR of 0.86 (95% CI 0.80–0.94).

### Association between metabolomic signature scores of UPF and MPF intake and the risk of T2D

Among 6010 participants, over a median follow-up of 18 years (105,318 person-years), 853 incident T2D cases were recorded. Higher UPF intake was associated with increased T2D risk in a dose-response manner (Table 3), with 30.6, 33.5, and 35.9% of cases occurring across increasing tertiles. After adjustment for sociodemographic and lifestyle factors, total energy intake, and clinical measures, the highest tertile was associated with a 20% elevated risk in T2D incidence (HR 1.20, 95% CI 1.08–1.33, p-trend = 0.008), which persisted after additional adjustment for diet quality indicators (HR 1.18, 95% CI 1.03–1.31, p-trend = 0.025). Each 1-SD increment in UPF intake was associated with a HR of 1.15 (95% CI 1.02–1.28) in the fully adjusted model. By contrast, higher MPF intake showed a significant inverse association with T2D risk, with an 18% reduced risk in Model 1 (tertile 3 vs. 1: HR 0.82, 95% CI 0.69–0.97, p-trend = 0.030) and a 15% reduced risk after adjusting for diet quality (Model 2: HR 0.85, 95% CI 0.73–0.97, p-trend = 0.034; per 1-SD HR 0.86, 95% CI 0.79–0.93).

**Table 3:** Prospective associations of tertiles of UPF and MPF intake and metabolic signature scores with incident type 2 diabetes in the Whitehall II cohort: multivariable Cox regression models (N = 6010; 853 T2D cases)

Exposure	Tertile 1 (low)	Tertile 2	Tertile 3 (high)	P-trend	Per 1 SD increase
***n***	2003	2003	2004		6010
**UPF intake**					
Events	261	286	306		853
Model 1	Reference	1.04 (0.89–1.23)	1.20 (1.08–1.33)	0.008	1.18 (1.04–1.32)
Model 2	Reference	1.02 (0.87–1.20)	1.18 (1.03–1.31)	0.025	1.15 (1.02–1.28)
**MPF intake**					
Events	285	291	277		853
Model 1	Reference	0.90 (0.75–1.08)	0.82 (0.69–0.97)	0.030	0.84 (0.76–0.92)
Model 2	Reference	0.88 (0.74–1.03)	0.85 (0.73–0.97)	0.034	0.86 (0.79–0.93)
**UPF metabolic signature**					
Events	274	270	309		853
Model 1	Reference	1.02 (0.87–1.25)	1.21 (1.02–1.43)	0.027	1.19 (1.05–1.38)
Model 2	Reference	0.99 (0.82–1.17)	1.20 (1.03–1.42)	0.030	1.18 (1.04–1.36)
**MPF metabolic signature**					
Events	331	288	234		853
Model 1	Reference	0.75 (0.64–0.88)	0.55 (0.43–0.67)	1.4 × 10^−10^	0.68 (0.59–0.79)
Model 2	Reference	0.79 (0.68–0.96)	0.60 (0.48–0.73)	4.9 × 10^−7^	0.72 (0.62–0.84)

Model 1: Adjusted for age at baseline, sex, ethnicity, education, employment grade, marital status, physical activity, alcohol consumption, smoking status, family history of CVD, total energy intake, BMI, hypertension, and dyslipidaemia

Model 2: Model 1 additionally adjusted for indicators of diet quality (total sugar, sodium, total fat, and fibre)

P-trend was calculated by treating the categorical variable as continuous using the median value within each category. Per 1 SD estimates represent the HR associated with each standard deviation increment in the exposure, modelled as a continuous variable

Abbreviations: *CI* confidence interval, *HR* hazard ratio, *MPF* minimally processed foods, *SD* standard deviation, *UPF* ultra-processed foods, *T2D* Type 2 diabetes

The UPF metabolomic signature showed a positive dose-response relationship with T2D risk, with 32.1, 31.7, and 36.2% of cases across increasing tertiles, and exhibited comparable associations to dietary UPF intake (tertile 3: HR 1.21, 95% CI 1.02–1.43, p-trend = 0.027 in Model 1; HR 1.20, 95% CI 1.03–1.42, p-trend = 0.030 in Model 2; per-1-SD HR 1.18, 95% CI 1.04–1.36). The MPF metabolomic signature showed stronger inverse associations with T2D risk than dietary MPF intake alone, with 38.8, 33.8, and 27.4% of cases across increasing tertiles. Participants in the highest tertile had a 45% lower risk in Model 1 (HR 0.55, 95% CI 0.43–0.67, p-trend = 1.4 × 10⁻¹⁰) and 40% lower risk in Model 2 (HR 0.60, 95% CI 0.48–0.73, p-trend = 4.9×10⁻⁷). Per 1-SD increment, the MPF metabolomic signature was associated with a HR of 0.72 (95% CI 0.62–0.84).

### Mediation analysis

Mediation analyses were conducted to examine whether the UPF- and MPF-associated metabolomic signatures mediated the associations between dietary intake and cardiometabolic outcomes (Table 4). For CVD, the UPF metabolomic signature mediated 51.1% (95% CI 41.8–71.3) of the total effect (NIE HR 1.09, 95% CI 1.03–1.23), while the MPF metabolomic signature mediated 46.6% (95% CI 34.6–75.9; NIE HR 0.93, 95% CI 0.85–0.98). Individual nutrient mediators (sugar, sodium, saturated fat) did not show statistically significant indirect effects for CVD with either UPF or MPF intake (Table 4).

**Table 4:** Mediation of associations between ultra-processed and minimally processed food intake and cardiometabolic outcomes by its metabolomic signatures and key nutrients (N = 6010)

Outcome	Mediator	Total effect HR (95% CI)^a^	Natural direct effect HR (95% CI)^b^	Natural indirect effect HR (95% CI)^c^	Proportion mediated % (95% CI)^d^
CVD					
UPF intake					
	Metabolomic signature	1.18 (1.02–1.37)	1.08 (1.02–1.24)	1.09 (1.03–1.23)	51.1 (41.8–71.3)
	Sugar		1.15 (1.07–1.31)	1.03 (0.97–1.24)	Null
	Sodium		1.19 (1.08–1.35)	0.99 (0.91–1.13)	Null
	Saturated fat		1.16 (1.06–1.33)	1.02 (0.98–1.27)	Null
MPF intake					
	Metabolomic signature	0.86 (0.78–0.95)	0.92 (0.87–0.97)	0.93 (0.85–0.98)	46.6 (34.6–75.9)
	Sugar		0.87 (0.81–0.94)	0.99 (0.91–1.03)	Null
	Sodium		0.88 (0.84–0.96)	0.98 (0.91–1.09)	Null
	Saturated fat		0.89 (0.86–0.96)	0.97 (0.95–1.03)	Null
T2D					
UPF intake					
	Metabolomic signature	1.19 (1.05–1.38)	1.11 (1.04–1.23)	1.08 (1.03–1.21)	42.8 (30.2–65.4)
	Sugar		1.14 (1.07–1.21)	1.05 (1.02–1.22)	26.8 (19.7–47.6)
	Sodium		1.17 (1.08–1.28)	1.03 (0.97–1.19)	Null
	Saturated fat		1.15 (1.09–1.32)	1.04 (0.94–1.18)	Null
MPF intake					
	Metabolomic signature	0.84 (0.76–0.92)	0.91 (0.87–0.97)	0.92 (0.89–0.98)	47.8 (28.9–59.1)
	Sugar		0.86 (0.79–0.91)	0.98 (0.93–1.02)	Null
	Sodium		0.82 (0.75–1.03)	1.02 (0.99–1.17)	Null
	Saturated fat		0.87 (0.74–1.09)	0.97 (0.91–1.08)	Null

Models adjusted for age at baseline, sex, ethnicity, education, employment grade, marital status, physical activity, alcohol consumption, smoking status, family history of CVD/T2D, total energy intake, BMI, hypertension, and dyslipidaemia

Total, natural direct, and natural indirect effects estimated using causal mediation models with the plasma UPF- and MPF-associated metabolic signatures as mediators. Each mediator was examined in a separate model

Abbreviations: *HR* hazard ratio, *CI* confidence interval, *CVD* cardiovascular disease, *MPF* minimally processed foods, *T2D* type 2 diabetes, *UPF* ultra-processed foods

^a^Total effect of dietary exposure on outcome

^b^Natural direct effect: effect of UPF/MPF intake on outcome not mediated through the specified mediator

^c^Natural indirect effect: effect of UPF/MPF intake on outcome mediated through the specified mediator

^d^Proportion mediated with 95% CI calculated using bootstrap methods (1000 resamples). Null indicates the natural indirect effect was not statistically significant (95% CI included the null value)

For T2D, the UPF metabolomic signature mediated 42.8% (95% CI 30.2–65.4) of the total effect (NIE HR 1.08, 95% CI 1.03–1.21). Notably, sugars also showed a significant indirect effect on the UPF–T2D association (NIE HR 1.05, 95% CI 1.02–1.22), with a proportion mediated of 26.8% (95% CI 19.7–47.6). Sodium and saturated fat did not significantly mediate this association. For MPF intake, the metabolomic signature mediated 47.8% (95% CI 28.9–59.1) of the association with T2D (NIE HR 0.92, 95% CI 0.89–0.98), while individual nutrients showed non-significant indirect effects.

### Subgroup and sensitivity analyses

In stratified analyses (Additional file 2: Table S10), associations were broadly consistent across subgroups of smoking status, sex, and BMI category, with no statistically significant interactions observed (P_int = 0.06–0.42).

Among UPF subgroups modelled separately, ultra-processed drinks (HR 1.08, 95% CI: 1.02–1.15 for CVD; 1.13, 1.02–1.24 for T2D) and meats (HR 1.17, 1.04–1.26 for CVD; 1.18, 1.03–1.30 for T2D) were significantly associated with both incident CVD and T2D, while ultra-processed breads were inversely associated with T2D (HR 0.85, 95% CI 0.79–0.92; Additional file 2: Table S11). No other subgroups showed significant associations with either outcome.

Sensitivity analyses, including MICE, complete-case analyses (N = 4551), exclusion of participants with incident events within two years of baseline, and additional adjustment for AHEI-2010, yielded largely consistent results (Additional file 2: Tables S12–S17). Under alternative Nova reclassification approaches (35 ambiguous FFQ items; Additional file 2: Table S18), Spearman correlations between reference and reclassified intakes were high (ρ ≥ 0.87), and associations between UPF and MPF intake and incident CVD and T2D remained statistically significant, though slightly attenuated under the lower bound scenario (e.g., UPF–T2D: HR 1.12, 95% CI 1.01–1.20 vs. reference HR 1.15, 1.02–1.28; Additional file 2: Table S19). When metabolomic signature models were further adjusted for self-reported UPF or MPF intake, effect estimates were modestly attenuated but remained significant (Additional file 2: Table S20). Re-deriving the signatures with confounders as unpenalised terms in the elastic net (UPF r = 0.98; MPF r = 0.97; Additional file 2: Table S21) and using phase 5 intake alone (UPF r = 0.94; MPF r = 0.91; Additional file 2: Table S22) produced signatures highly correlated with the primary signatures, and with associations materially unchanged in both. Harrell’s C-index for CVD improved from 0.642 (covariates only) to 0.655 with the addition of MPF intake and its metabolomic signature; for T2D, the corresponding improvement was from 0.735 to 0.757, with metabolomic signatures consistently providing greater incremental discrimination than self-reported intake alone (Additional file 2: Table S23).

## Discussion

Leveraging metabolomic data from 6010 participants, we derived distinct metabolomic signature scores for habitual UPF and MPF intake assessed over a five-year dietary assessment period. These signatures showed divergent associations with incident CVD and T2D over up to 20 years of follow-up, with the UPF signature associated with elevated cardiometabolic risk and the MPF signature inversely associated, independent of clinical risk factors, indicators of diet quality and sociodemographic factors. Mediation analyses suggested that these metabolomic profiles substantially explained the dietary intake–disease associations. To our knowledge, no prior studies have examined the prospective associations of UPF- and MPF- related metabolomic signature scores with incident CVD and T2D.

### Findings in context

Prior metabolomics studies using both LC-MS and NMR platforms in large population cohorts from the UK, US, and Sweden have consistently identified an adverse metabolomic profile associated with higher UPF intake, and have linked these signatures to diverse outcomes, including MASLD, diabetic microvascular complications, mental disorders, and all-cause mortality [23–25, 43–46]. Our findings were broadly consistent with this literature, particularly in showing lower DHA, omega-3, and HDL-related measures, including ApoA1 and HDL phospholipid features, together with a more atherogenic VLDL and triglyceride-related profile.

Despite this overall concordance, we observed differences in specific metabolite classes that have been inconsistently reported in prior work. For lipid subclasses, total cholesterol and selected VLDL measures showed positive associations that appear weaker or null elsewhere, potentially attributable to differences in subclass resolution and adjustment strategy [45]. For amino acids, tyrosine contributed negatively to the UPF signature, mirroring inverse associations reported in children [46], although aromatic amino acids had limited prominence in NMR-based UPF signatures from larger adult cohorts [22, 43]. This pattern is further supported by our food-level correlations (Fig. 2), in which tyrosine was positively correlated with minimally processed protein sources (meats and poultry, fish, beans, grains) rather than with UPF. Lower circulating aromatic amino acids in relation to UPF intake may therefore indicate displacement of protein-rich whole foods by carbohydrate- and fat-dense UPFs in this midlife cohort, rather than the insulin-resistant amino acid profile described previously [47].

Directions for ketone bodies and glycolysis intermediates also vary across studies because these metabolites are highly sensitive to metabolic state and sampling conditions. Acetoacetate and acetone were inversely associated with UPF in both our signature and the UK Biobank [22], while pyruvate and citrate were additionally identified in our model, in line with reports of elevated glycolysis intermediates linked to high sugar dietary patterns [22, 46]. At the food subgroup level, this heterogeneity was also evident: pyruvate showed strong positive correlations with ultra-processed sweets, breads, and meat products, but weaker associations with drinks and cereals [48, 49]. The correlations with sweets and breads are consistent with elevated glycolytic flux related to refined carbohydrate content [50], aligning with reports that circulating pyruvate and lactate characterise high-sugar dietary patterns and are prospectively associated with insulin resistance and T2D risk [51]. Despite this within-category heterogeneity, the overall pattern of UPF metabolite associations remained broadly consistent with prior signature-based analyses of UPF intake [25, 52].

Evidence on metabolomic profiles related to MPF intake is comparatively limited, with only one recent UK Biobank study directly addressing this [22], and recent randomised trials have also assessed processing contrasts, reporting changes in HDL cholesterol and LDL cholesterol that were not fully consistent across studies [52–54]. In our cohort, the MPF signature reflected a composite of nutritionally distinct contributions rather than a uniform metabolic effect [53]: omega-3 and HDL-related features appeared to be driven primarily by fish, whereas amino acid and citrate associations were distributed across grains, beans, and vegetables. Positive associations with phenylalanine may reflect higher intakes of high-quality protein sources, although this metabolite is not specific to MPF and may also index broader dietary patterns [54, 55]. Cross-cohort differences in metabolomic signature composition may additionally reflect variation in the distribution of food subtypes consumed within each Nova category.

### Potential mechanisms of UPF/MPF metabolomic signatures associated with risk of CVD and T2D

The distinct metabolomic signatures of UPF and MPF intake were independently associated with CVD and T2D after adjustment for dietary indicators, clinical risk factors, and adiposity, suggesting that they capture cardiometabolic variation beyond self-reported dietary exposure and conventional dietary indicators [13, 56]. Associations also remained significant after additional adjustment for UPF or MPF intake, indicating that the signatures reflect metabolic information not fully captured by dietary assessment alone. This residual information may arise from improved measurement of habitual intake, inter-individual variation in metabolic response, and gut microbiome-mediated processing [57]. Supplementary C-index analyses confirmed modestly higher discrimination when metabolomic signatures were added to models (Additional file 2: Table S23); however, as these signatures were derived to reflect dietary patterns rather than optimise prediction, replication in independent cohorts is needed.

The most prominent mechanistic feature of the UPF signature was an atherogenic lipoprotein pattern compatible with insulin-resistant dyslipidaemia, in which higher hepatic substrate load and de novo lipogenesis promote VLDL overproduction, while downstream lipoprotein remodelling is associated with lower HDL-related measures, including ApoA1 and a less favourable lipoprotein profile. Given the central role of ApoA1 in HDL formation and cholesterol efflux, this pattern is consistent with reduced reverse cholesterol transport [58, 59]. By contrast, the MPF signature was characterised by higher PUFAs, particularly DHA, and a more favourable HDL profile, features that were attenuated or absent in the UPF signature [60–64]. This pattern is consistent with evidence that nutrient-dense diets rich in fish, whole grains, nuts, and legumes are associated with favourable lipoprotein distributions [65–67]. Biologically, higher circulating PUFAs and DHA may reflect a more favourable fatty acid composition and lipid metabolic profile, potentially linked to improved triglyceride metabolism and lower inflammation [68].

The metabolomic signatures mediated 43 to 51% of the total association across all exposure–outcome pairs, substantially more than individual nutrients. Sodium and saturated fat did not significantly mediate any association, and sugar mediated only the UPF–T2D pathway, accounting for a smaller proportion than the corresponding metabolomic signature (26.8 vs 42.8%; full pathway-specific estimates in Additional file 2: Table S24). This pattern aligns with findings from the Moli-sani cohort, where sugar was the only individual nutrient to meaningfully mediate the UPF–mortality association (up to 36.3%), saturated fat contributed negligibly, and all nutrients combined explained no more than 41% of the strongest outcome [69]. Together, these results support the interpretation that food processing may influence cardiometabolic risk through mechanisms beyond individual nutrient pathways, including structural changes to the food matrix, compounds formed during processing, additives used in UPFs, and chemicals migrating from packaging [70–72]. The unmediated proportion likely reflects mechanisms not captured by the Nightingale NMR platform, including gut microbial metabolites, inflammatory pathways, and non-metabolomic factors such as appetite dysregulation and altered satiety signalling [73, 74], which warrant further investigation.

### Strengths and limitations

This is, to our knowledge, the first investigation to derive metabolomic signature scores for both UPF and MPF and examine their prospective associations with long-term CVD and T2D risk. We used repeated dietary measures across two phases to characterise habitual intake, with dietary assessment preceding outcome ascertainment. The 20-year follow-up provides one of the longest prospective evaluations of UPF intake in relation to cardiometabolic risk in a UK population.

Several limitations warrant consideration. First, the Nightingale NMR platform, while comprehensive, cannot capture certain biomarkers of food processing such as those related to additives, packaging-derived contaminants, or gut microbiota alterations [74–77], and it remains difficult to distinguish metabolites directly reflecting dietary exposure from downstream endogenous responses. Second, the Nova classification, while the most widely used system and recently adopted by the Scientific Advisory Committee on Nutrition (SACN) [78], may have limitations when applied to the Whitehall II FFQ, which was not designed to capture all details needed for precise categorisation. Self-reported dietary data are subject to recall bias, though evidence suggests good agreement between FFQ-based UPF estimates and 24-h dietary recalls [79]. Additionally, as ultra-processing intensity and market penetration have increased since our dietary data collection period (1991–2003), our findings likely represent conservative estimates of present-day associations. Third, missing data may not be entirely missing at random, potentially introducing bias; however, complete-case analyses yielded similar results (Additional file 2: Tables S13–S14).

Fourth, the metabolomic signatures were derived and their disease associations tested within the same cohort. Although we applied elastic net regularisation with 10-fold cross-validation and a 70/30 training and testing split to mitigate overfitting of the signature itself, these strategies constitute internal rather than external validation. Estimates from the signature derivation, disease association, and mediation analyses may therefore be optimistic, and generalisability is further limited by cohort-specific dietary patterns and platform-specific metabolite quantification. Moreover, although we applied a causal mediation framework, the estimates rely on four identifying assumptions (A1–A4; Additional file 1: Fig. S5) that cannot be empirically verified in observational data [80]. The high-dimensional derivation of the metabolomic signature may also capture shared variance with unmeasured confounders, meaning that reported mediation proportions should be interpreted with caution [81]. These findings should be considered hypothesis-generating, and replication in independent cohorts with comparable dietary and metabolomic data, ideally incorporating MS-based platforms, would strengthen the evidence base.

Fifth, although the Whitehall II study uses a multi-criterion approach to outcome ascertainment combining NHS record linkage, clinical examinations, and verified self-report, some degree of outcome misclassification is expected over a 20-year follow-up period. Such misclassification is likely non-differential with respect to dietary exposure and would therefore bias results toward the null. The number of incident CVD and T2D events may also have limited power to detect modest associations and to examine effect modification. Sixth, as with all observational studies, causality cannot be inferred, and residual confounding may remain despite comprehensive adjustment. Finally, the predominantly midlife British civil servant cohort may limit generalisability to more socioeconomically and ethnically diverse populations, although cardiometabolic risk factor associations from the Whitehall II cohort have been shown to be broadly consistent with those from nationally representative samples [82].

## Conclusions

In conclusion, we identified multi-metabolite signatures of habitual UPF and MPF intake using an NMR-based approach that showed opposing prospective associations with the risk of CVD and T2D in a midlife occupational cohort. The mediation of these associations by the metabolomic signature scores suggests that these metabolites may capture biological effects beyond those reflected by conventional measures of diet quality. Replication in diverse populations with different metabolomic platforms, including MS-based approaches, would help strengthen the evidence base for potential biological pathways linking UPF intake to cardiometabolic risk.

## Supplementary information

Supplementary Material 1: Additional file 1: Figures S1–S5.

Supplementary Material 2: Additional file 2: Tables S1–S24.

## Data Availability

Owing to constraints imposed by the study’s ethics approval and participants consent, Whitehall II data are not publicly available for download. Data, protocols, and other metadata are, however, available to researchers through a managed data-sharing process, subject to approval and a data-sharing agreement. Please refer to the Whitehall II data sharing policy at https://www.ucl.ac.uk/psychiatry/research/mental-health-older-people/whitehall-ii/data-sharing.

## Abbreviations

AHEI-2010: Alternative Healthy Eating Index 2010
BMI: Body Mass Index
CI: Confidence Interval
CVD: Cardiovascular Disease
FFQ: Food Frequency Questionnaire
HDL: High Density Lipoprotein
HES: Hospital Episode Statistics
HR: Hazard Ratio
ICD: International Classification of Diseases
IDL: Intermediate Density Lipoprotein
IQR: Interquartile Range
LDL: Low Density Lipoprotein
MPF: Minimally Processed Foods
NMR: Nuclear Magnetic Resonance
PUFA: Polyunsaturated Fatty Acids
SACN: Scientific Advisory Committee on Nutrition
SES: Socioeconomic Status
T2D: Type 2 Diabetes
TCA: Tricarboxylic Acid Cycle
UPF: Ultra Processed Foods
VLDL: Very Low Density Lipoprotein
