# Supplementary material for: Circulating metabolomic signatures of ultra-processed and minimally processed food intake and risk of cardiovascular disease and type 2 diabetes: 20-year follow-up in the Whitehall II cohort
Source: BMC Med. 2026 Jul 21;24:535. doi: 10.1186/s12916-026-05081-7 (PMC13632048; doi:10.1186/s12916-026-05081-7)
Supplement: Supplementary file 1 [file 12916_2026_5081_MOESM1_ESM.docx]

**List of Supplementary Figures**

Fig. S1. Study timeline of the Whitehall II cohort showing dietary exposure assessment, metabolomics measurement, and follow-up periods for CVD and T2D.

Fig. S2. Flowchart of study sample selection and schematic study design.

Fig. S3. Correlation between UPF and MPF intake and the corresponding metabolomic signature scores.

Fig. S4. Overlapping metabolites associated with UPF and MPF intake.

Fig. S5. Directed acyclic graph (DAG) for the causal mediation analysis of UPF and MPF intake and incident CVD/T2D via metabolomic and nutrient pathways.

**Fig. S1.** Study timeline of the Whitehall II cohort showing dietary exposure assessment, metabolomics measurement, and follow-up periods for CVD and T2D.

**Fig. S2.** Flowchart of study sample selection and schematic study design.

**Fig. S3.** Correlation between UPF and MPF intake and the corresponding metabolomic signature scores. Colours indicate training versus testing sets (70/30 split). Signature scores were calculated as weighted sums of standardised metabolites selected by elastic net in the training set and applied to the test set. The line shows the fitted linear association; r is the overall Pearson correlation.

**Fig. S4.** Overlapping metabolites associated with UPF and MPF intake.

**Fig. S5.** Directed acyclic graph (DAG) for the causal mediation analysis of UPF and MPF intake and incident CVD/T2D via metabolomic and nutrient pathways. A (exposure): UPF/MPF intake; M1 (mediator): metabolomic signature; M2 (mediator): sugar, sodium, and saturated fat; Y (outcome): incident CVD/T2D; C1 (covariates): sociodemographic and lifestyle factors; C2 (covariates): clinical factors. The diagram encodes the no-unmeasured-confounding assumptions (A1–A4) required for causal mediation analysis.
