# Supplementary material for: Circulating metabolomic signatures of ultra-processed and minimally processed food intake and risk of cardiovascular disease and type 2 diabetes: 20-year follow-up in the Whitehall II cohort
Source: BMC Med. 2026 Jul 21;24:535. doi: 10.1186/s12916-026-05081-7 (PMC13632048; doi:10.1186/s12916-026-05081-7)
Supplement: Supplementary file 2 [file 12916_2026_5081_MOESM2_ESM.docx]

**List of Supplementary Tables**

Table S1. Comparison of included/excluded Whitehall II participants’ characteristics at baseline (phase 5, 1997-1999)

Table S2. Complete list and description of the 233 metabolites analysed in the Whitehall II cohort

Table S3. Nova classification of 127 food frequency questionnaire food items

Table S4. Ultra-processed food and minimally processed food subgroup classification

Table S5. ICD-9 and ICD-10 codes for cardiovascular diseases and type 2 diabetes

Table S6. Distribution of variables with missing data before and after multiple imputation

Table S7. Cross-sectional associations between NMR-derived metabolites and ultra-processed food (UPF) intake in the Whitehall II cohort

Table S8. Cross-sectional associations between 233 metabolites and minimally processed food (MPF) intake in the Whitehall II cohort

Table S9. Comparison of characteristics between training (N=4,207) and testing (N=1,803) sets used for metabolomic signature derivation

Table S10. Stratified analyses: associations of UPF and MPF intake and their metabolomic signatures (per 1-SD) with incident CVD and T2D in the Whitehall II cohort (N=6,010)

Table S11. Associations between UPF subgroup intake (per 1-SD increment, %g/day) and incident CVD and T2D in the Whitehall II cohort (N=6,010)

Table S12. Sensitivity analysis using multiple imputation by chained equations (MICE): prospective associations of tertiles of UPF and MPF intake and metabolomic signature scores with incident CVD and T2D in the Whitehall II cohort (N=6,010)

Table S13. Complete-case sensitivity analysis: associations between UPF/MPF metabolomic signature scores and risk of CVD in the Whitehall II cohort (N=4,551; 998 CVD events)

Table S14. Complete-case sensitivity analysis: associations between UPF/MPF metabolomic signature scores and risk of T2D in the Whitehall II cohort (N=4,551; 742 T2D events)

Table S15. Two-year lag sensitivity analysis: associations of UPF intake, MPF intake and metabolomic signature scores with incident CVD in the Whitehall II cohort (N=5,970; CVD events=1,008)

Table S16. Two-year lag sensitivity analysis: associations of UPF intake, MPF intake, and metabolomic signature scores with incident T2D in the Whitehall II cohort (N=5,970; T2D events=832)

Table S17. Sensitivity analysis: associations of UPF intake, MPF intake, and metabolomic signature scores with incident CVD and T2D, additionally adjusted for AHEI-2010, in the Whitehall II cohort (N=6,010)

Table S18. Nova classification sensitivity analysis using lower and upper bound assumptions for reclassification

Table S19. Nova reclassification sensitivity analysis: associations between UPF/MPF intake and risk of CVD and T2D in the Whitehall II cohort (N=6,010)

Table S20. Associations of UPF and MPF metabolomic signature scores with incident CVD and T2D after additional adjustment for dietary UPF or MPF intake

Table S21. Sensitivity analysis: UPF and MPF metabolomic signatures re-derived with confounders entered as unpenalised terms in the elastic net

Table S22. Sensitivity analysis for dietary exposure timing: intake consistency and phase 5–only metabolomic signatures

Table S23. Comparison of discriminative performance (Harrell’s C-index) for incident CVD and T2D across models

Table S24. Pathway-specific estimates from causal mediation analyses: exposure–mediator and mediator–outcome associations for UPF and MPF intake with incident CVD and T2D in the Whitehall II cohort (N=6,010)

Table S1. Comparison of included/excluded Whitehall II participants’ characteristics at baseline (phase 5, 1997-1999)

|  |  | Participants in 1997/1999 (n=8,628) | |
| --- | --- | --- | --- |
| Characteristics |  | **Included (n=6,010)** | **Excluded (n=2,618)** |
| Socioeconomic factors | | | |
| Sex | Male | 76.7 | 73.7 |
|  | Female | 23.3 | 26.3 |
| Age, years |  | 55.8 ± 5.9 | 57.8 ± 6.1 |
| Ethnicity | White | 90.8 | 90.3 |
|  | Non-White | 9.2 | 9.7 |
| Marital status | Yes | 76.1 | 75.2 |
|  | No | 23.9 | 24.8 |
| Employment grade | Low | 15.3 | 17.4 |
|  | Medium | 46.4 | 45.9 |
|  | High | 38.3 | 36.7 |
| Education level | < Secondary | 35.5 | 32.9 |
|  | Secondary | 25.0 | 26.6 |
|  | University | 39.5 | 40.5 |
| Health behaviour factors | | | |
| Smoking status | Current smokers | 9.2 | 10.2 |
|  | Former smokers | 37.4 | 34.6 |
|  | Non-smokers | 53.4 | 55.2 |
| Alcohol consumption | Abstainers | 16.8 | 18.1 |
|  | Moderate | 48.0 | 57.5 |
|  | High | 35.2 | 32.3 |
| Physical activity | Inactive | 37.4 | 36.3 |
|  | Moderately active | 17.8 | 15.4 |
|  | Active | 44.8 | 48.3 |
| Health status factors | | | |
| Hypertension | Yes | 20.0 | 17.9 |
|  | No | 80.0 | 72.1 |
| Family history of CVD | Yes | 10.0 | 8.0 |
|  | No | 90.0 | 92.0 |
| Family history of T2D | Yes | 8.4 | 8.9 |
|  | No | 91.6 | 91.1 |
| Body mass index, kg/m² |  | 26.1 ± 15.4 | 26.5 ± 4.2 |
| Dyslipidaemia | Yes | 18.5 | 16.5 |
|  | No | 81.5 | 84.5 |

Abbreviations: CVD, cardiovascular disease; SD, standard deviation; T2D, type 2 diabetes.

Values are column percentages for categorical variables and mean ± SD for continuous variables.

Table S2. Complete list and description of the 233 metabolites analysed in the Whitehall II cohort

| Name | Label | Pathway |
| --- | --- | --- |
| Acetate | Acetate (mmol/l) | Ketone bodies |
| Acetoacetate | Acetoacetate (mmol/l) | Ketone bodies |
| Acetone | Acetone (mmol/l) | Ketone bodies |
| Ala | Alanine (mmol/l) | Amino acid metabolism |
| ApoA1 | Apolipoprotein A1 (g/l) | Apolipoproteins |
| ApoB | Apolipoprotein B (g/l) | Apolipoproteins |
| ApoB_by_ApoA1 | Ratio of apolipoprotein B to apolipoprotein A1 (ratio) | Apolipoproteins |
| Cholines | Total cholines (mmol/l) | Choline metabolism |
| Citrate | Citrate (mmol/l) | Glycolysis / TCA cycle |
| Creatinine | Creatinine (mmol/l) | Renal function |
| DHA | Docosahexaenoic acid (mmol/l) | Other / Unknown |
| DHA_pct | Ratio of docosahexaenoic acid to total fatty acids (%) | Fatty acid metabolism |
| Gln | Glutamine (mmol/l) | Amino acid metabolism |
| Glucose | Glucose (mmol/l) | Glycolysis / TCA cycle |
| Gly | Glycine (mmol/l) | Amino acid metabolism |
| GlycA | Glycoprotein acetyls (mmol/l) | Inflammation |
| Glycerol | Glycerol (mmol/l) | Glycolysis / TCA cycle |
| HDL_C | HDL cholesterol (mmol/l) | Lipoprotein metabolism |
| HDL_CE | Cholesteryl esters in HDL (mmol/l) | Lipoprotein metabolism |
| HDL_FC | Free cholesterol in HDL (mmol/l) | Lipoprotein metabolism |
| HDL_L | Total lipids in HDL (mmol/l) | Lipoprotein metabolism |
| HDL_P | Concentration of HDL particles (mmol/l) | Lipoprotein metabolism |
| HDL_PL | Phospholipids in HDL (mmol/l) | Lipoprotein metabolism |
| HDL_TG | Triglycerides in HDL (mmol/l) | Lipoprotein metabolism |
| HDL_size | Average diameter for HDL particles (nm) | Lipoprotein metabolism |
| IDL_C | Cholesterol in IDL (mmol/l) | Lipoprotein metabolism |
| IDL_CE | Cholesteryl esters in IDL (mmol/l) | Lipoprotein metabolism |
| IDL_CE_pct | Cholesteryl esters to total lipids ratio in IDL (%) | Lipoprotein metabolism |
| IDL_C_pct | Cholesterol to total lipids ratio in IDL (%) | Lipoprotein metabolism |
| IDL_FC | Free cholesterol in IDL (mmol/l) | Lipoprotein metabolism |
| IDL_FC_pct | Free cholesterol to total lipids ratio in IDL (%) | Lipoprotein metabolism |
| IDL_L | Total lipids in IDL (mmol/l) | Lipoprotein metabolism |
| IDL_P | Concentration of IDL particles (mmol/l) | Lipoprotein metabolism |
| IDL_PL | Phospholipids in IDL (mmol/l) | Lipoprotein metabolism |
| IDL_PL_pct | Phospholipids to total lipids ratio in IDL (%) | Lipoprotein metabolism |
| IDL_TG | Triglycerides in IDL (mmol/l) | Lipoprotein metabolism |
| IDL_TG_pct | Triglycerides to total lipids ratio in IDL (%) | Lipoprotein metabolism |
| LA | Linoleic acid (mmol/l) | Other / Unknown |
| LA_pct | Ratio of linoleic acid to total fatty acids (%) | Fatty acid metabolism |
| LDL_C | LDL cholesterol (mmol/l) (s5) | Lipoprotein metabolism |
| LDL_CE | Cholesteryl esters in LDL (mmol/l) | Lipoprotein metabolism |
| LDL_FC | Free cholesterol in LDL (mmol/l) | Lipoprotein metabolism |
| LDL_L | Total lipids in LDL (mmol/l) | Lipoprotein metabolism |
| LDL_P | Concentration of LDL particles (mmol/l) | Lipoprotein metabolism |
| LDL_PL | Phospholipids in LDL (mmol/l) | Lipoprotein metabolism |
| LDL_TG | Triglycerides in LDL (mmol/l) | Lipoprotein metabolism |
| LDL_size | Average diameter for LDL particles (nm) | Lipoprotein metabolism |
| L_HDL_C | Cholesterol in large HDL (mmol/l) | Lipoprotein metabolism |
| L_HDL_CE | Cholesteryl esters in large HDL (mmol/l) | Lipoprotein metabolism |
| L_HDL_CE_pct | Cholesteryl esters to total lipids ratio in large HDL (%) | Lipoprotein metabolism |
| L_HDL_C_pct | Cholesterol to total lipids ratio in large HDL (%) | Lipoprotein metabolism |
| L_HDL_FC | Free cholesterol in large HDL (mmol/l) | Lipoprotein metabolism |
| L_HDL_FC_pct | Free cholesterol to total lipids ratio in large HDL (%) | Lipoprotein metabolism |
| L_HDL_L | Total lipids in large HDL (mmol/l) | Lipoprotein metabolism |
| L_HDL_P | Concentration of large HDL particles (mmol/l) | Lipoprotein metabolism |
| L_HDL_PL | Phospholipids in large HDL (mmol/l) | Lipoprotein metabolism |
| L_HDL_PL_pct | Phospholipids to total lipids ratio in large HDL (%) | Lipoprotein metabolism |
| L_HDL_TG | Triglycerides in large HDL (mmol/l) | Lipoprotein metabolism |
| L_HDL_TG_pct | Triglycerides to total lipids ratio in large HDL (%) | Lipoprotein metabolism |
| L_LDL_C | Cholesterol in large LDL (mmol/l) | Lipoprotein metabolism |
| L_LDL_CE | Cholesteryl esters in large LDL (mmol/l) | Lipoprotein metabolism |
| L_LDL_CE_pct | Cholesteryl esters to total lipids ratio in large LDL (%) | Lipoprotein metabolism |
| L_LDL_C_pct | Cholesterol to total lipids ratio in large LDL (%) | Lipoprotein metabolism |
| L_LDL_FC | Free cholesterol in large LDL (mmol/l) | Lipoprotein metabolism |
| L_LDL_FC_pct | Free cholesterol to total lipids ratio in large LDL (%) | Lipoprotein metabolism |
| L_LDL_L | Total lipids in large LDL (mmol/l) | Lipoprotein metabolism |
| L_LDL_P | Concentration of large LDL particles (mmol/l) | Lipoprotein metabolism |
| L_LDL_PL | Phospholipids in large LDL (mmol/l) | Lipoprotein metabolism |
| L_LDL_PL_pct | Phospholipids to total lipids ratio in large LDL (%) | Lipoprotein metabolism |
| L_LDL_TG | Triglycerides in large LDL (mmol/l) (s5) | Lipoprotein metabolism |
| L_LDL_TG_pct | Triglycerides to total lipids ratio in large LDL (%) | Lipoprotein metabolism |
| L_VLDL_C | Cholesterol in large VLDL (mmol/l) | Lipoprotein metabolism |
| L_VLDL_CE | Cholesteryl esters in large VLDL (mmol/l) | Lipoprotein metabolism |
| L_VLDL_CE_pct | Cholesteryl esters to total lipids ratio in large VLDL (%) | Lipoprotein metabolism |
| L_VLDL_C_pct | Cholesterol to total lipids ratio in large VLDL (%) | Lipoprotein metabolism |
| L_VLDL_FC | Free cholesterol in large VLDL (mmol/l) | Lipoprotein metabolism |
| L_VLDL_FC_pct | Free cholesterol to total lipids ratio in large VLDL (%) | Lipoprotein metabolism |
| L_VLDL_L | Total lipids in large VLDL (mmol/l) | Lipoprotein metabolism |
| L_VLDL_P | Concentration of large VLDL particles (mmol/l) | Lipoprotein metabolism |
| L_VLDL_PL | Phospholipids in large VLDL (mmol/l) | Lipoprotein metabolism |
| L_VLDL_PL_pct | Phospholipids to total lipids ratio in large VLDL (%) | Lipoprotein metabolism |
| L_VLDL_TG | Triglycerides in large VLDL (mmol/l) | Lipoprotein metabolism |
| L_VLDL_TG_pct | Triglycerides to total lipids ratio in large VLDL (%) | Lipoprotein metabolism |
| Lactate | Lactate (mmol/l) | Glycolysis / TCA cycle |
| MUFA | Monounsaturated fatty acids (mmol/l) | Fatty acid metabolism |
| MUFA_pct | Ratio of monounsaturated fatty acids to total fatty acids (%) | Fatty acid metabolism |
| M_HDL_C | Cholesterol in medium HDL (mmol/l) | Lipoprotein metabolism |
| M_HDL_CE | Cholesteryl esters in medium HDL (mmol/l) | Lipoprotein metabolism |
| M_HDL_CE_pct | Cholesteryl esters to total lipids ratio in medium HDL (%) | Lipoprotein metabolism |
| M_HDL_C_pct | Cholesterol to total lipids ratio in medium HDL (%) | Lipoprotein metabolism |
| M_HDL_FC | Free cholesterol in medium HDL (mmol/l) | Lipoprotein metabolism |
| M_HDL_FC_pct | Free cholesterol to total lipids ratio in medium HDL (%) | Lipoprotein metabolism |
| M_HDL_L | Total lipids in medium HDL (mmol/l) | Lipoprotein metabolism |
| M_HDL_P | Concentration of medium HDL particles (mmol/l) | Lipoprotein metabolism |
| M_HDL_PL | Phospholipids in medium HDL (mmol/l) | Lipoprotein metabolism |
| M_HDL_PL_pct | Phospholipids to total lipids ratio in medium HDL (%) | Lipoprotein metabolism |
| M_HDL_TG | Triglycerides in medium HDL (mmol/l) | Lipoprotein metabolism |
| M_HDL_TG_pct | Triglycerides to total lipids ratio in medium HDL (%) | Lipoprotein metabolism |
| M_LDL_C | Cholesterol in medium LDL (mmol/l) | Lipoprotein metabolism |
| M_LDL_CE | Cholesteryl esters in medium LDL (mmol/l) | Lipoprotein metabolism |
| M_LDL_CE_pct | Cholesteryl esters to total lipids ratio in medium LDL (%) | Lipoprotein metabolism |
| M_LDL_C_pct | Cholesterol to total lipids ratio in medium LDL (%) | Lipoprotein metabolism |
| M_LDL_FC | Free cholesterol in medium LDL (mmol/l) | Lipoprotein metabolism |
| M_LDL_FC_pct | Free cholesterol to total lipids ratio in medium LDL (%) | Lipoprotein metabolism |
| M_LDL_L | Total lipids in medium LDL (mmol/l) | Lipoprotein metabolism |
| M_LDL_P | Concentration of medium LDL particles (mmol/l) | Lipoprotein metabolism |
| M_LDL_PL | Phospholipids in medium LDL (mmol/l) | Lipoprotein metabolism |
| M_LDL_PL_pct | Phospholipids to total lipids ratio in medium LDL (%) | Lipoprotein metabolism |
| M_LDL_TG | Triglycerides in medium LDL (mmol/l) | Lipoprotein metabolism |
| M_LDL_TG_pct | Triglycerides to total lipids ratio in medium LDL (%) | Lipoprotein metabolism |
| M_VLDL_C | Cholesterol in medium VLDL (mmol/l) | Lipoprotein metabolism |
| M_VLDL_CE | Cholesteryl esters in medium VLDL (mmol/l) | Lipoprotein metabolism |
| M_VLDL_CE_pct | Cholesteryl esters to total lipids ratio in medium VLDL (%) | Lipoprotein metabolism |
| M_VLDL_C_pct | Cholesterol to total lipids ratio in medium VLDL (%) | Lipoprotein metabolism |
| M_VLDL_FC | Free cholesterol in medium VLDL (mmol/l) | Lipoprotein metabolism |
| M_VLDL_FC_pct | Free cholesterol to total lipids ratio in medium VLDL (%) | Lipoprotein metabolism |
| M_VLDL_L | Total lipids in medium VLDL (mmol/l) | Lipoprotein metabolism |
| M_VLDL_P | Concentration of medium VLDL particles (mmol/l) | Lipoprotein metabolism |
| M_VLDL_PL | Phospholipids in medium VLDL (mmol/l) | Lipoprotein metabolism |
| M_VLDL_PL_pct | Phospholipids to total lipids ratio in medium VLDL (%) | Lipoprotein metabolism |
| M_VLDL_TG | Triglycerides in medium VLDL (mmol/l) | Lipoprotein metabolism |
| M_VLDL_TG_pct | Triglycerides to total lipids ratio in medium VLDL (%) | Lipoprotein metabolism |
| Omega_3 | Omega-3 fatty acids (mmol/l) | Fatty acid metabolism |
| Omega_3_pct | Ratio of omega-3 fatty acids to total fatty acids (%) | Fatty acid metabolism |
| Omega_6 | Omega-6 fatty acids (mmol/l) | Fatty acid metabolism |
| Omega_6_by_Omega_3 | Ratio of omega-6 fatty acids to omega-3 fatty acids (ratio) | Fatty acid metabolism |
| Omega_6_pct | Ratio of omega-6 fatty acids to total fatty acids (%) | Fatty acid metabolism |
| PUFA | Polyunsaturated fatty acids (mmol/l) | Fatty acid metabolism |
| PUFA_by_MUFA | Ratio of polyunsaturated fatty acids to monounsaturated fatty acids (ratio) | Fatty acid metabolism |
| PUFA_pct | Ratio of polyunsaturated fatty acids to total fatty acids (%) | Fatty acid metabolism |
| Phe | Phenylalanine (mmol/l) | Amino acid metabolism |
| Phosphatidylc | Phosphatidylcholines (mmol/l) | Choline metabolism |
| Phosphoglyc | Phosphoglycerides (mmol/l) | Membrane lipid metabolism |
| Pyruvate | Pyruvate (mmol/l) | Glycolysis / TCA cycle |
| SFA | Saturated fatty acids (mmol/l) | Fatty acid metabolism |
| SFA_pct | Ratio of saturated fatty acids to total fatty acids (%) | Fatty acid metabolism |
| S_HDL_C | Cholesterol in small HDL (mmol/l) | Lipoprotein metabolism |
| S_HDL_CE | Cholesteryl esters in small HDL (mmol/l) | Lipoprotein metabolism |
| S_HDL_CE_pct | Cholesteryl esters to total lipids ratio in small HDL (%) | Lipoprotein metabolism |
| S_HDL_C_pct | Cholesterol to total lipids ratio in small HDL (%) | Lipoprotein metabolism |
| S_HDL_FC | Free cholesterol in small HDL (mmol/l) | Lipoprotein metabolism |
| S_HDL_FC_pct | Free cholesterol to total lipids ratio in small HDL (%) | Lipoprotein metabolism |
| S_HDL_L | Total lipids in small HDL (mmol/l) | Lipoprotein metabolism |
| S_HDL_P | Concentration of small HDL particles (mmol/l) | Lipoprotein metabolism |
| S_HDL_PL | Phospholipids in small HDL (mmol/l) | Lipoprotein metabolism |
| S_HDL_PL_pct | Phospholipids to total lipids ratio in small HDL (%) | Lipoprotein metabolism |
| S_HDL_TG | Triglycerides in small HDL (mmol/l) | Lipoprotein metabolism |
| S_HDL_TG_pct | Triglycerides to total lipids ratio in small HDL (%) | Lipoprotein metabolism |
| S_LDL_C | Cholesterol in small LDL (mmol/l) | Lipoprotein metabolism |
| S_LDL_CE | Cholesteryl esters in small LDL (mmol/l) | Lipoprotein metabolism |
| S_LDL_CE_pct | Cholesteryl esters to total lipids ratio in small LDL (%) | Lipoprotein metabolism |
| S_LDL_C_pct | Cholesterol to total lipids ratio in small LDL (%) | Lipoprotein metabolism |
| S_LDL_FC | Free cholesterol in small LDL (mmol/l) | Lipoprotein metabolism |
| S_LDL_FC_pct | Free cholesterol to total lipids ratio in small LDL (%) | Lipoprotein metabolism |
| S_LDL_L | Total lipids in small LDL (mmol/l) | Lipoprotein metabolism |
| S_LDL_P | Concentration of small LDL particles (mmol/l) | Lipoprotein metabolism |
| S_LDL_PL | Phospholipids in small LDL (mmol/l) | Lipoprotein metabolism |
| S_LDL_PL_pct | Phospholipids to total lipids ratio in small LDL (%) | Lipoprotein metabolism |
| S_LDL_TG | Triglycerides in small LDL (mmol/l) | Lipoprotein metabolism |
| S_LDL_TG_pct | Triglycerides to total lipids ratio in small LDL (%) | Lipoprotein metabolism |
| S_VLDL_C | Cholesterol in small VLDL (mmol/l) | Lipoprotein metabolism |
| S_VLDL_CE | Cholesteryl esters in small VLDL (mmol/l) | Lipoprotein metabolism |
| S_VLDL_CE_pct | Cholesteryl esters to total lipids ratio in small VLDL (%) | Lipoprotein metabolism |
| S_VLDL_C_pct | Cholesterol to total lipids ratio in small VLDL (%) | Lipoprotein metabolism |
| S_VLDL_FC | Free cholesterol in small VLDL (mmol/l) | Lipoprotein metabolism |
| S_VLDL_FC_pct | Free cholesterol to total lipids ratio in small VLDL (%) | Lipoprotein metabolism |
| S_VLDL_L | Total lipids in small VLDL (mmol/l) | Lipoprotein metabolism |
| S_VLDL_P | Concentration of small VLDL particles (mmol/l) | Lipoprotein metabolism |
| S_VLDL_PL | Phospholipids in small VLDL (mmol/l) | Lipoprotein metabolism |
| S_VLDL_PL_pct | Phospholipids to total lipids ratio in small VLDL (%) | Lipoprotein metabolism |
| S_VLDL_TG | Triglycerides in small VLDL (mmol/l) | Lipoprotein metabolism |
| S_VLDL_TG_pct | Triglycerides to total lipids ratio in small VLDL (%) | Lipoprotein metabolism |
| Sphingomyelins | Sphingomyelins (mmol/l) | Membrane lipid metabolism |
| TG_by_PG | Ratio of triglycerides to phosphoglycerides (ratio) | Lipid metabolism |
| Tyr | Tyrosine (mmol/l) | Amino acid metabolism |
| Unsaturation | Degree of unsaturation (degree) | Other / Unknown |
| VLDL_C | VLDL cholesterol (mmol/l) | Lipoprotein metabolism |
| VLDL_CE | Cholesteryl esters in VLDL (mmol/l) | Lipoprotein metabolism |
| VLDL_FC | Free cholesterol in VLDL (mmol/l) | Lipoprotein metabolism |
| VLDL_L | Total lipids in VLDL (mmol/l) | Lipoprotein metabolism |
| VLDL_P | Concentration of VLDL particles (mmol/l) | Lipoprotein metabolism |
| VLDL_PL | Phospholipids in VLDL (mmol/l) | Lipoprotein metabolism |
| VLDL_TG | Triglycerides in VLDL (mmol/l) | Lipoprotein metabolism |
| VLDL_size | Average diameter for VLDL particles (nm) | Lipoprotein metabolism |
| XL_HDL_C | Cholesterol in very large HDL (mmol/l) | Lipoprotein metabolism |
| XL_HDL_CE | Cholesteryl esters in very large HDL (mmol/l) | Lipoprotein metabolism |
| XL_HDL_CE_pct | Cholesteryl esters to total lipids ratio in very large HDL (%) | Lipoprotein metabolism |
| XL_HDL_C_pct | Cholesterol to total lipids ratio in very large HDL (%) | Lipoprotein metabolism |
| XL_HDL_FC | Free cholesterol in very large HDL (mmol/l) | Lipoprotein metabolism |
| XL_HDL_FC_pct | Free cholesterol to total lipids ratio in very large HDL (%) | Lipoprotein metabolism |
| XL_HDL_L | Total lipids in very large HDL (mmol/l) | Lipoprotein metabolism |
| XL_HDL_P | Concentration of very large HDL particles (mmol/l) | Lipoprotein metabolism |
| XL_HDL_PL | Phospholipids in very large HDL (mmol/l) | Lipoprotein metabolism |
| XL_HDL_PL_pct | Phospholipids to total lipids ratio in very large HDL (%) | Lipoprotein metabolism |
| XL_HDL_TG | Triglycerides in very large HDL (mmol/l) | Lipoprotein metabolism |
| XL_HDL_TG_pct | Triglycerides to total lipids ratio in very large HDL (%) | Lipoprotein metabolism |
| XL_VLDL_C | Cholesterol in very large VLDL (mmol/l) | Lipoprotein metabolism |
| XL_VLDL_CE | Cholesteryl esters in very large VLDL (mmol/l) | Lipoprotein metabolism |
| XL_VLDL_CE_pct | Cholesteryl esters to total lipids ratio in very large VLDL (%) | Lipoprotein metabolism |
| XL_VLDL_C_pct | Cholesterol to total lipids ratio in very large VLDL (%) | Lipoprotein metabolism |
| XL_VLDL_FC | Free cholesterol in very large VLDL (mmol/l) | Lipoprotein metabolism |
| XL_VLDL_FC_pct | Free cholesterol to total lipids ratio in very large VLDL (%) | Lipoprotein metabolism |
| XL_VLDL_L | Total lipids in very large VLDL (mmol/l) | Lipoprotein metabolism |
| XL_VLDL_P | Concentration of very large VLDL particles (mmol/l) | Lipoprotein metabolism |
| XL_VLDL_PL | Phospholipids in very large VLDL (mmol/l) | Lipoprotein metabolism |
| XL_VLDL_PL_pct | Phospholipids to total lipids ratio in very large VLDL (%) | Lipoprotein metabolism |
| XL_VLDL_TG | Triglycerides in very large VLDL (mmol/l) | Lipoprotein metabolism |
| XL_VLDL_TG_pct | Triglycerides to total lipids ratio in very large VLDL (%) | Lipoprotein metabolism |
| XS_VLDL_C | Cholesterol in very small VLDL (mmol/l) | Lipoprotein metabolism |
| XS_VLDL_CE | Cholesteryl esters in very small VLDL (mmol/l) | Lipoprotein metabolism |
| XS_VLDL_CE_pct | Cholesteryl esters to total lipids ratio in very small VLDL (%) | Lipoprotein metabolism |
| XS_VLDL_C_pct | Cholesterol to total lipids ratio in very small VLDL (%) | Lipoprotein metabolism |
| XS_VLDL_FC | Free cholesterol in very small VLDL (mmol/l) | Lipoprotein metabolism |
| XS_VLDL_FC_pct | Free cholesterol to total lipids ratio in very small VLDL (%) | Lipoprotein metabolism |
| XS_VLDL_L | Total lipids in very small VLDL (mmol/l) | Lipoprotein metabolism |
| XS_VLDL_P | Concentration of very small VLDL particles (mmol/l) | Lipoprotein metabolism |
| XS_VLDL_PL | Phospholipids in very small VLDL (mmol/l) | Lipoprotein metabolism |
| XS_VLDL_PL_pct | Phospholipids to total lipids ratio in very small VLDL (%) | Lipoprotein metabolism |
| XS_VLDL_TG | Triglycerides in very small VLDL (mmol/l) | Lipoprotein metabolism |
| XS_VLDL_TG_pct | Triglycerides to total lipids ratio in very small VLDL (%) | Lipoprotein metabolism |
| XXL_VLDL_C | Cholesterol in chylomicrons and extremely large VLDL (mmol/l) | Lipoprotein metabolism |
| XXL_VLDL_CE | Cholesteryl esters in chylomicrons and extremely large VLDL (mmol/l) | Lipoprotein metabolism |
| XXL_VLDL_CE_pct | Cholesteryl esters to total lipids ratio in chylomicrons and extremely large VLDL (%) | Lipoprotein metabolism |
| XXL_VLDL_C_pct | Cholesterol to total lipids ratio in chylomicrons and extremely large VLDL (%) | Lipoprotein metabolism |
| XXL_VLDL_FC | Free cholesterol in chylomicrons and extremely large VLDL (mmol/l) | Lipoprotein metabolism |
| XXL_VLDL_FC_pct | Free cholesterol to total lipids ratio in chylomicrons and extremely large VLDL (%) | Lipoprotein metabolism |
| XXL_VLDL_L | Total lipids in chylomicrons and extremely large VLDL (mmol/l) | Lipoprotein metabolism |
| XXL_VLDL_P | Concentration of chylomicrons and extremely large VLDL particles (mmol/l) | Lipoprotein metabolism |
| XXL_VLDL_PL | Phospholipids in chylomicrons and extremely large VLDL (mmol/l) | Lipoprotein metabolism |
| XXL_VLDL_PL_pct | Phospholipids to total lipids ratio in chylomicrons and extremely large VLDL (%) | Lipoprotein metabolism |
| XXL_VLDL_TG | Triglycerides in chylomicrons and extremely large VLDL (mmol/l) | Lipoprotein metabolism |
| XXL_VLDL_TG_pct | Triglycerides to total lipids ratio in chylomicrons and extremely large VLDL (%) | Lipoprotein metabolism |
| bOHbutyrate | 3-Hydroxybutyrate (mmol/l) | Ketone bodies |

Table S3. Nova classification of 127 food frequency questionnaire food items

| Food category | Food description | Nova Category |
| --- | --- | --- |
| Bread | Average brown bread/rolls | UPF |
| Bread | Average crispbread | UPF |
| Bread | Average white bread/rolls | UPF |
| Bread | Average whole meal bread/rolls | UPF |
| Cereals | Average All-Bran, Bran Flakes | UPF |
| Cereals | Average Corn Flakes, Rice Krispies | UPF |
| Cereals | Average Frosties, Ricicles etc | UPF |
| Cereals | Average Muesli, Fruit &Fibre | UPF |
| Cereals | Average Shredded wheat, Weetabix | UPF |
| Cereals | Average porridge, Readybrek | MPF |
| Dairy | Average Channel Islands milk | MPF |
| Dairy | Average cheese - Cheddar, Brie | PF |
| Dairy | Average cottage cheese, low fat soft | PF |
| Dairy | Average double or clotted cream | PCI |
| Dairy | Average dried milk | MPF |
| Dairy | Average full cream milk | MPF |
| Dairy | Average semi-skimmed milk | MPF |
| Dairy | Average single cream | PCI |
| Dairy | Average skimmed milk | MPF |
| Dairy | Average sterilized milk | MPF |
| Dairy | Average yoghurt | MPF |
| Drink | Average Horlicks, Ovaltine | UPF |
| Drink | Average beer, lager or cider | PF |
| Drink | Average coffee whitener | UPF |
| Drink | Average coffee, decaffeinated | MPF |
| Drink | Average coffee, regular | MPF |
| Drink | Average fizzy soft drinks | UPF |
| Drink | Average fruit squash or cordial | UPF |
| Drink | Average liqueurs | UPF |
| Drink | Average port, cherry, vermouth | UPF |
| Drink | Average low cal, diet fizzy drinks | UPF |
| Drink | Average real fruit juice | MPF |
| Drink | Average spirits | UPF |
| Drink | Average tea | MPF |
| Drink | Average wine | PF |
| Eggs | Average eggs | MPF |
| Fat | Average butter | PCI |
| Fat | Average hard margarine | UPF |
| Fat | Average low fat spread | UPF |
| Fat | Average other soft margarine | UPF |
| Fat | Average peanut butter | PF |
| Fat | Average polyunsaturated margarine | UPF |
| Fish | Average fish fingers, fish cakes | UPF |
| Fish | Average fried fish in batter | UPF |
| Fish | Average oily fish - fresh/canned | MPF |
| Fish | Average shellfish | MPF |
| Fish | Average white fish - fresh/frozen | MPF |
| Fruit | Average apples | MPF |
| Fruit | Average bananas | MPF |
| Fruit | Average dried fruit:raisin,prune | MPF |
| Fruit | Average grapefruit | MPF |
| Fruit | Average grapes | MPF |
| Fruit | Average melon | MPF |
| Fruit | Average oranges, satsumas | MPF |
| Fruit | Average peaches, plums, apricots | MPF |
| Fruit | Average pears | MPF |
| Fruit | Average strawberries, raspberries | MPF |
| Fruit | Average tinned fruit | PF |
| Meat | Average bacon | UPF |
| Meat | Average beef - roast, steak etc | MPF |
| Meat | Average beef burgers | UPF |
| Meat | Average chicken or other poultry | MPF |
| Meat | Average corned beef, luncheon m. | UPF |
| Meat | Average ham | UPF |
| Meat | Average lamb - roast, chops, stew | MPF |
| Meat | Average liver, liver pate | UPF |
| Meat | Average meat soup | UPF |
| Meat | Average pork - roast, chops, stew | MPF |
| Meat | Average sausages | UPF |
| Meat | Average savory pies | UPF |
| Pasta | Average lasagne | UPF |
| Pasta | Average pizza | UPF |
| Pasta | Average quiche | UPF |
| Pasta | Average white or green pasta | MPF |
| Pasta | Average wholemeal pasta | MPF |
| Potato | Average boiled, mashed potatoes | MPF |
| Potato | Average chips or French fries | UPF |
| Potato | Average potato salad | PF |
| Potato | Average roast potatoes | MPF |
| Rice | Average brown rice | MPF |
| Rice | Average white rice | MPF |
| Sauces | Average Marmite, Bovril | UPF |
| Sauces | Average french dressing, vinaigre | UPF |
| Sauces | Average pickles, chutney | UPF |
| Sauces | Average salad cream | UPF |
| Sauces | Average sauces - white, gravy | UPF |
| Sauces | Average tomato ketchup | UPF |
| Sweets/snacks | Average biscuits | UPF |
| Sweets/snacks | Average buns and pastries | UPF |
| Sweets/snacks | Average cakes | UPF |
| Sweets/snacks | Average chocolates, choc bars | UPF |
| Sweets/snacks | Average cocoa, hot chocolate | UPF |
| Sweets/snacks | Average cream crack, cheese biscuits | UPF |
| Sweets/snacks | Average crisps, packet snacks | UPF |
| Sweets/snacks | Average fruit pies,tarts,crumble | UPF |
| Sweets/snacks | Average ice cream, choc ices | UPF |
| Sweets/snacks | Average jam, marmalade, honey | UPF |
| Sweets/snacks | Average milk puddings | UPF |
| Sweets/snacks | Average sponge puddings | UPF |
| Sweets/snacks | Average sugar in tea, coffee | PCI |
| Sweets/snacks | Average sweets, toffees, mints | UPF |
| Vegetable | Average Brussels sprouts | MPF |
| Vegetable | Average baked beans | UPF |
| Vegetable | Average beans - green, broad, etc | MPF |
| Vegetable | Average broccoli | MPF |
| Vegetable | Average cabbage | MPF |
| Vegetable | Average carrots | MPF |
| Vegetable | Average cauliflower | MPF |
| Vegetable | Average coleslaw | UPF |
| Vegetable | Average dried lentils, beans,etc | MPF |
| Vegetable | Average garlic | MPF |
| Vegetable | Average green salad | MPF |
| Vegetable | Average leeks | MPF |
| Vegetable | Average marrow, courgettes | MPF |
| Vegetable | Average mushrooms | MPF |
| Vegetable | Average onions | MPF |
| Vegetable | Average parsnip, turnip, swede | MPF |
| Vegetable | Average peanuts and other nuts | MPF |
| Vegetable | Average peas | MPF |
| Vegetable | Average soya meat, TVP, vegburger | UPF |
| Vegetable | Average spinach | MPF |
| Vegetable | Average spring greens, kale | MPF |
| Vegetable | Average sweet peppers | MPF |
| Vegetable | Average tofu or soya bean curd | UPF |
| Vegetable | Average tomatoes | MPF |
| Vegetable | Average vegetable soup | UPF |

Table S4. Ultra-processed food and minimally processed food subgroup classification

| UPF Subgroup | Food Items |
| --- | --- |
| Drinks | Horlicks, coffee whitener, fizzy soft drinks, fruit squash, liqueurs, port, low-calorie diet fizzy drinks, spirits |
| Dairy products | Soya milk, ice-cream |
| Sauces and spreads | Hard margarine, soft margarine, low-fat spread, polyunsaturated margarine, ketchup, Marmite, salad cream, gravy, chutney, baked beans, coleslaw |
| Meats, fish and alternatives | Bacon, beef burgers, corned beef, ham, liver pâté, meat soup, sausages, savoury pies, fish fingers, fried fish, tofu, TVP, soya meat |
| Sweets | Biscuits, buns and pastries, cakes, chocolates, cocoa, fruit pies, jam, milk puddings, sponge puddings, sweets |
| Savoury snacks | Crisps, cream crackers, chips, fried potato |
| Cereals | Bran Flakes, Corn Flakes, Frosties, Muesli, Shredded Wheat, Weetabix |
| Ready-to-eat meals | Pizza, lasagna, quiche, vegetable soup, meat soup |
| Breads | White bread, brown bread, wholegrain bread |
| MPF Subgroup | **Food Items** |
| Beverages | Coffee (decaffeinated), coffee (regular), real fruit juice, tea |
| Dairy products | Channel Island milk, dried milk, full cream milk, semi-skimmed milk, skimmed milk, sterilized milk, yogurt |
| Fish | Oily fish, shellfish, white fish |
| Meat and poultry | Beef, pork, lamb, chicken, eggs |
| Fruits | Apples, bananas, dried fruits, grapefruit, grapes, melon, oranges, peaches, pears, strawberries |
| Vegetables | Boiled potato, roast potatoes, broccoli, cabbage, carrots, cauliflower, marrow, mushrooms, onions, parsnip, spinach, kale, sweet peppers, tomatoes, peanuts, sprouts |
| Grains | White pasta, green pasta, wholemeal pasta, brown rice, white rice |
| Beans | Green beans, lentils, peas |

Table S5. ICD-9 and ICD-10 codes for cardiovascular diseases and type 2 diabetes

| Outcome | ICD-9 | ICD-10 |
| --- | --- | --- |
| Cardiovascular diseases (CVD) | | |
| Stroke | 430, 431, 434, 436 | I60, I61, I63-64 |
| Myocardial infarction | 410 | I21 |
| Definite angina | 410 | I20 |
| Heart failure | — | I50 |
| Peripheral artery disease | 250.7, 440.2, 440.4, 443.8-9, 444.2, 444.81 | I70.2, I73.3, I73.9, I74.3-5, E10.5, E115, E12.5, E13.5, E14.5 |
| Coronary heart diseases | 410-414 | I20-I25 |
| Type 2 diabetes (T2D) | | |
| Type 2 diabetes | 250 | E11 |

Abbreviations: Cardiovascular diseases; ICD, International classification code; T2D, Type II diabetes.

Table S6. Distribution of variables with missing data before and after multiple imputation

| Characteristics | Before imputation | After imputation  N=6,010 | After imputation  (MICE)  N=6,010 | Number (%) with missing data |
| --- | --- | --- | --- | --- |
| UPF | 19.3 ± 7.6 | 20.1 ± 8.5 | 19.1± 8.6 | -^b^ |
| MPF | 55.9 ± 6.0 | 61.0 ± 11.52 | 60.8±11.4 | -^b^ |
| Marital status |  | |  | 194 (3.2) |
| Yes | 4416 (75.9) | 4574 (76.1) | 4239 (70.5) |  |
| No | 1400 (24.1) | 1436 (23.9) | 1771 (29.5) |  |
| Family history of CVD | | |  | 98 (2.3) |
| Yes | 510 (8.6) | 599 (10.0) | 480 (8.0) |  |
| No | 5402 (91.4) | 5411 (90.0) | 5530 (92.0) |  |
| Family history of T2D |  |  |  | 124 (2.1) |
| Yes | 498 (8.5) | 504 (8.4) | 511 (8.5) |  |
| No | 5388 (91.5) | 5506 (91.6) | 5499 (91.5) |  |
| Education | | |  | 1459 (24.3) |
| < Secondary | 1344 (29.5) | 2132 (35.5) | 1943 (32.3) |  |
| Secondary | 1246 (27.4) | 1501 (25.0) | 1589 (26.4) |  |
| University | 1961 (43.1) | 2377 (39.5) | 2478 (41.2) |  |
| Employment grade | | |  | 408 (6.8) |
| Administrative | 790 (14.9) | 918 (15.3) | 820 (13.6) |  |
| Prof/executive | 2706 (51.0) | 2791 (46.4) | 3041 (50.6) |  |
| Clerical/support | 1806 (34.1) | 2301 (38.3) | 2149 (35.8) |  |
| Physical activity | | |  | 375 (6.2) |
| Inactive | 2115 (37.5) | 2249 (37.4) | 2272 (37.8) |  |
| Active | 2532 (44.9) | 2694 (44.8) | 2701(44.9) |  |
| Moderate active | 988 (17.5) | 1067 (17.8) | 1037 (17.3) |  |
| Smoking | | |  | 480 (8.0) |
| Never smoking | 2889 (52.2) | 3210 (53.4) | 3172 (52.8) |  |
| Former smoker | 2192 (39.6) | 2248 (37.4) | 2152 (35.8) |  |
| Current smoker | 449 (8.2) | 552 (9.2) | 686 (11.4) |  |
| Alcohol consumption | | |  | 238 (4.0) |
| Abstainers | 1005 (17.4) | 1011 (16.8) | 1021(17.0) |  |
| Moderate drinkers | 2775 (48.1) | 2884 (48.0) | 2891 (48.1) |  |
| Heavy drinkers | 1992 (34.5) | 2115 (35.2) | 2098 (11.4) |  |
| Body mass index (kg/m²) | 26.1 ± 15.4 | 26.3 ± 15.2 | 27.2 ± 14.8 | 568 (10.8) |
| Hypertension |  |  |  | 130 (2.2) |
| Yes | 1167 (19.8) | 1199 (20.0) | 1103 (18.4) |  |
| No | 4713 (80.2) | 4811 (80.0) | 4907 (81.6) |  |
| Dyslipidaemia |  |  |  | 147 (2.4) |
| Yes | 1024 (17.5) | 1113 (18.5) | 1098 (18.3) |  |
| No | 4839 (82.5) | 4897 (81.5) | 4912 (81.7) |  |
| Sodium intake (mg/day) | 2711.0 ± 1020.5 | 2713.4 ± 1021.3 | 2709 ±1018.3 | 326 (4.6) |
| Sugar intake (g/day) | 132.9 ± 48.9 | 134.2 ± 50.6 | 133.8±50.9 | 476 (6.7) |
| Fat intake (g/day) | 77.7 ± 30.7 | 76.9 ± 29.5 | 76.9 ± 29.5 | 433 (6.1) |
| Saturated fat (g/day) | 26.1 ± 16.5 | 29.8 ± 17.9 | 28.6± 17.5 | 433 (6.1) |
| Total energy intake (kcal/day) | 2168 ± 593 | 2197 ± 596 | 2191 ± 588 | 326 (4.6) |

**Abbreviations**: CVD, cardiovascular diseases; T2D, type 2 diabetes; SD, standard deviation.

^a^Dietary intake was assessed at phases 3 and 5. Participants missing data at both phases were excluded (n = 1374). Among included participants, 516 (8.6%) were missing dietary data at phase 3 and 980 (16.3%) at phase 5.

^b^ Percentages calculated based on non-missing observations for each variable. Values for continuous variables shown as mean ± (standard deviation) SD

Table S7. Cross-sectional associations between NMR-derived metabolites and ultra-processed food (UPF) intake in the Whitehall II cohort

| Metabolite | β (95% CI) | P value |
| --- | --- | --- |
| Acetoacetate | -0.0220 (-0.0310, -0.0010) | < 0.0001 |
| Ala | -0.0032 (-0.0042, -0.0010) | < 0.0001 |
| Creatinine | -0.0020 (-0.0030, -0.0010) | < 0.0001 |
| Acetone | -0.0180 (-0.0230, -0.0010) | < 0.0001 |
| Acetate | -0.0020 (-0.0030, -0.0016) | 0.0002 |
| ApoA1 | -0.0800 (-0.08100, -0.0500) | < 0.0001 |
| ApoB | -0.0007 (-0.0178, -0.0000) | 0.001 |
| ApoB_by_ApoA1 | 0.0020 (0.0010, 0.0080) | 0.002 |
| Gln | 0.0040 (0.0022, 0.0089) | 0.0001 |
| GlycA | 0.0020 (0.0001, 0.0030) | 0.0001 |
| Gly | -0.0020 (-0.0099, -0.0010) | 0.0001 |
| Citrate | 0.0560 (0.0420, 0.0660) | < 0.0001 |
| Glycerol | 0.0030 (0.0021, 0.0050) | 0.0001 |
| Lactate | -0.0130 (-0.0250, -0.0030) | < 0.0001 |
| LA | -0.0020 (-0.0030, 0.0020) | 0.140 |
| LA_pct | 0.230 (0.160, 0.380) | < 0.0001 |
| CRP | 0.0290 (0.0016, 0.0430) | < 0.0001 |
| VLDL_TG | 0.0040 (-0.0060, 0.0050) | 0.110 |
| VLDL_L | 0.0030 (-0.0060, 0.0040) | 0.110 |
| VLDL_C | 0.0015 (-0.0066, 0.0030) | 0.110 |
| S_VLDL_C | 0.0022 (-0.0070, 0.0030) | 0.180 |
| MUFA_pct | 0.0200 (0.0066, 0.0350) | < 0.0001 |
| L_LDL_TG_pct | -0.0005 (-0.0011, 0.0090) | 0.060 |
| L_LDL_TG | -0.0001 (-0.0020, 0.0080) | 0.110 |
| L_LDL_L | -0.0210 (-0.0290, -0.0030) | < 0.0001 |
| L_LDL_C | 0.0220 (0.0130, 0.0310) | 0.0001 |
| L_LDL_PL | 0.0060 (0.0030, 0.0110) | 0.001 |
| L_LDL_CE | -0.0140 (-0.0210, -0.0020) | 0.0001 |
| L_LDL_FC | -0.0020 (-0.0080, -0.0010) | 0.034 |
| L_LDL_P | 0.0070 (0.0040, 0.0090) | 0.0001 |
| L_LDL_CE_pct | 0.0050 (0.0020, 0.0060) | 0.0001 |
| L_LDL_PL_pct | 0.0020 (0.0120, 0.0070) | 0.010 |
| XXL_VLDL_C | 0.0060 (-0.0010, 0.0090) | 0.110 |
| XXL_VLDL_L | 0.0060 (-0.0090, 0.0090) | 0.210 |
| XL_VLDL_L | 0.0058 (-0.0012, 0.0090) | 0.220 |
| XL_VLDL_C | 0.0055 (0.0001, 0.0100) | 0.001 |
| XL_HDL_L | -0.124 (-0.155, 0.006) | 0.220 |
| XL_HDL_FC_pct | 0.139 (0.008, 0.156) | 0.001 |
| XL_HDL_C | -0.0120 (-0.0190, 0.0080) | 0.120 |
| L_HDL_CE_pct | -0.0130 (-0.0170, -0.0080) | 0.0008 |
| L_HDL_CE | -0.0010 (-0.0017, 0.0080) | 0.110 |
| L_HDL_FC | -0.0000 (-0.0030, 0.0060) | 0.080 |
| L_HDL_FC_pct | -0.0250 (-0.0330, 0.0070) | 0.080 |
| L_HDL_L | -0.0143 (-0.0190, 0.0005) | 0.110 |
| L_HDL_P | -0.0000 (-0.0080, 0.0015) | 0.110 |
| L_HDL_PL | -0.0002 (-0.0040, 0.0100) | 0.070 |
| L_HDL_PL_pct | -0.0290 (-0.0310, -0.0020) | 0.0001 |
| L_HDL_TG | -0.0430 (-0.0510, -0.0190) | < 0.0001 |
| L_HDL_C | -0.0230 (-0.0330, -0.0100) | < 0.0001 |
| L_VLDL_C | 0.0047 (-0.0020, 0.0090) | 0.120 |
| L_VLDL_TG | 0.0040 (0.0010, 0.0080) | < 0.0001 |
| L_VLDL_PL_pct | 0.110 (0.080, 0.190) | < 0.0001 |
| L_VLDL_P | 0.0830 (0.0710, 0.1400) | < 0.0001 |
| L_VLDL_PL | 0.0210 (-0.0040, 0.0340) | 0.210 |
| L_VLDL_TG_pct | 0.120 (0.008, 0.190) | < 0.0001 |
| L_VLDL_FC_pct | 0.0670 (0.0330, 0.1200) | < 0.0001 |
| L_VLDL_CE | 0.0790 (0.0610, 0.0980) | < 0.0001 |
| L_VLDL_FC | 0.0010 (-0.0050, 0.0020) | 0.060 |
| L_VLDL_L | 0.0055 (0.0030, 0.0060) | < 0.0001 |
| M_LDL_FC_pct | -0.0050 (-0.0060, -0.0030) | < 0.0001 |
| M_LDL_L | 0.0160 (0.0080, 0.0220) | < 0.0001 |
| M_LDL_C_pct | 0.0590 (0.0450, 0.1300) | 0.0001 |
| M_LDL_FC | 0.0020 (0.0010, 0.0080) | 0.033 |
| M_LDL_C | -0.0150 (-0.0190, -0.0050) | < 0.0001 |
| Phe | -0.0080 (-0.0160, -0.0020) | < 0.0001 |
| Phosphoglyc | -0.0130 (-0.0180, -0.0080) | 0.0001 |
| Cholines | -0.0030 (-0.0050, -0.0015) | 0.001 |
| PUFA_pct | -0.0080 (-0.0110, -0.0040) | < 0.0001 |
| PUFA | -0.0097 (-0.0150, -0.0048) | < 0.0001 |
| Omega_6_by_Omega_3 | 0.0600 (0.0350, 0.0800) | < 0.0001 |
| Omega_6_pct | 0.0150 (0.0030, 0.0400) | < 0.0001 |
| Omega_6 | 0.0340 (0.0240, 0.0460) | 0.001 |
| Omega_3_pct | -0.280 (-0.400, -0.110) | < 0.0001 |
| Omega_3 | -0.0180 (-0.0210, -0.0010) | < 0.0001 |
| DHA | -0.203 (-0.390, -0.100) | 0.001 |
| DHA_pct | -0.0100 (-0.0650, 0.0069) | 0.200 |
| S_HDL_C_pct | -0.0330 (-0.0400, 0.0090) | 0.750 |
| S_HDL_C | 0.0050 (0.0030, 0.0090) | < 0.0001 |
| SFA_pct | -0.0490 (-0.0570, -0.0210) | < 0.0001 |
| SFA | -0.130 (-0.210, -0.090) | 0.001 |
| HDL_C | -0.0110 (-0.0150, -0.0087) | 0.017 |
| Adiponectin | -0.0050 (-0.0085, -0.0016) | < 0.0001 |
| XXL_VLDL_TG_pct | 0.0990 (0.0750, 0.1300) | < 0.0001 |
| XXL_VLDL_TG | 0.0060 (0.0038, 0.0110) | 0.040 |
| XXL_VLDL_PL | 0.0690 (0.0150, 0.0980) | < 0.0001 |
| XXL_VLDL_P | 0.0500 (0.0300, 0.0800) | < 0.0001 |
| XXL_VLDL_FC_pct | -0.0980 (-0.1900, -0.0120) | < 0.0001 |
| XXL_VLDL_FC | 0.0070 (0.0030, 0.0120) | 0.001 |
| XXL_VLDL_CE | 0.0080 (0.0070, 0.0190) | 0.0001 |
| XS_VLDL_PL_pct | 0.0020 (-0.0180, 0.0030) | 0.060 |
| XS_VLDL_FC_pct | 0.0030 (-0.0130, 0.0060) | 0.700 |
| XS_VLDL_TG_pct | -0.0500 (-0.0630, 0.0160) | 0.310 |
| XS_VLDL_CE_pct | -0.0880 (-0.1200, -0.0050) | < 0.0001 |
| XL_VLDL_TG | 0.0070 (-0.0010, 0.0090) | 0.062 |
| XL_VLDL_PL_pct | 0.0240 (0.0010, 0.0300) | 0.030 |
| XL_VLDL_P | -0.0080 (-0.0100, -0.0030) | < 0.0001 |
| XL_VLDL_C_pct | -0.0700 (-0.0810, -0.0030) | 0.0001 |
| XL_VLDL_FC | -0.0080 (-0.0100, -0.0030) | 0.0001 |
| XL_VLDL_CE_pct | -0.0760 (-0.1400, -0.0070) | 0.001 |
| XL_VLDL_CE | -0.0070 (-0.0090, -0.0020) | 0.0001 |
| XL_HDL_TG_pct | -0.0080 (-0.0170, -0.0030) | < 0.0001 |
| XL_HDL_TG | -0.0050 (-0.0180, -0.0030) | 0.035 |
| XL_HDL_PL_pct | -0.0590 (-0.0650, -0.0090) | 0.0003 |
| XL_HDL_PL | -0.0097 (-0.0170, -0.0080) | < 0.0001 |
| XL_HDL_P | -0.0110 (-0.0210, 0.0100) | 0.990 |
| XL_HDL_FC | -0.0080 (-0.0110, 0.0100) | 0.088 |
| XL_HDL_C_pct | -0.0500 (-0.0700, 0.0030) | 0.080 |
| XL_HDL_CE_pct | -0.0200 (-0.0280, -0.0040) | < 0.0001 |
| XL_HDL_CE | -0.0100 (-0.0130, -0.0030) | < 0.0001 |
| VLDL_size | 0.0260 (0.0030, 0.0420) | < 0.0001 |
| VLDL_PL | 0.0046 (0.0040, 0.0130) | < 0.0001 |
| VLDL_P | 0.0330 (-0.0110, 0.0420) | 0.800 |
| VLDL_FC | 0.0050 (-0.0020, 0.0090) | 0.066 |
| VLDL_CE | 0.0039 (-0.0045, 0.0140) | 0.068 |
| Unsaturation | -0.0500 (-0.0600, -0.0130) | 0.005 |
| TG_by_PG | 0.0090 (-0.0080, 0.0150) | 0.980 |
| Sphingomyelins | -0.0080 (-0.0160, 0.0070) | 0.560 |
| S_VLDL_TG_pct | 0.0003 (-0.0080, 0.0060) | 0.310 |
| S_VLDL_TG | 0.0020 (0.0002, 0.0030) | < 0.0001 |
| S_VLDL_CE_pct | 0.0120 (-0.0150, 0.0180) | 0.340 |
| S_VLDL_C_pct | 0.0120 (0.0050, 0.0140) | < 0.0001 |
| S_VLDL_L | 0.0500 (-0.0080, 0.0600) | 0.070 |
| S_VLDL_PL | 0.0040 (0.0010, 0.0110) | < 0.0001 |
| S_VLDL_P | 0.0040 (0.0023, 0.0100) | < 0.0001 |
| S_VLDL_FC | 0.0040 (0.0020, 0.0110) | 0.0006 |
| S_VLDL_FC_pct | 0.0020 (0.0013, 0.0040) | 0.0003 |
| S_VLDL_PL_pct | -0.0030 (-0.0230, 0.0010) | 0.330 |
| S_VLDL_CE | 0.0050 (0.0038, 0.0075) | 0.001 |
| PUFA_by_MUFA | -0.0098 (-0.0130, -0.0060) | 0.001 |
| M_VLDL_C_pct | 0.0080 (0.0040, 0.0130) | 0.001 |
| M_VLDL_PL_pct | 0.0069 (0.0023, 0.0100) | 0.003 |
| M_VLDL_FC_pct | 0.0070 (0.0020, 0.0110) | 0.001 |
| M_VLDL_TG_pct | 0.0059 (0.0010, 0.0110) | < 0.0001 |
| M_VLDL_TG | 0.0060 (-0.0030, 0.0100) | 0.063 |
| M_VLDL_PL | 0.0500 (0.0100, 0.1000) | < 0.0001 |
| M_VLDL_P | 0.0700 (0.0080, 0.0810) | < 0.0001 |
| M_VLDL_L | 0.0050 (0.0013, 0.0100) | 0.0001 |
| M_VLDL_FC | 0.0040 (-0.0020, 0.0090) | 0.270 |
| M_VLDL_CE_pct | 0.0010 (-0.0020, 0.0030) | 0.180 |
| M_VLDL_CE | 0.0040 (-0.0060, 0.0050) | 0.890 |
| M_VLDL_C | 0.0030 (0.0020, 0.0080) | 0.031 |
| M_LDL_TG_pct | -0.0090 (-0.0010, -0.0020) | 0.0001 |
| M_LDL_TG | -0.0050 (-0.0060, 0.0090) | 0.430 |
| M_LDL_PL_pct | -0.0030 (-0.0040, 0.0070) | 0.710 |
| M_LDL_PL | 0.0030 (-0.0010, 0.0090) | 0.240 |
| M_LDL_P | -0.0040 (-0.0080, 0.0020) | 0.270 |
| M_LDL_CE_pct | -0.0130 (-0.0170, -0.0080) | 0.001 |
| M_LDL_CE | -0.0060 (-0.0180, -0.0010) | 0.010 |
| M_HDL_TG | -0.0030 (-0.0180, 0.0020) | 0.260 |
| M_HDL_PL_pct | 0.0040 (-0.0170, 0.0080) | 0.810 |
| M_HDL_PL | -0.0600 (-0.0900, -0.0300) | < 0.0001 |
| M_HDL_P | -0.0230 (-0.0340, -0.0060) | 0.0001 |
| M_HDL_C | -0.0002 (-0.0006, 0.0100) | 0.890 |
| M_HDL_FC | -0.0140 (-0.0210, 0.0100) | 0.370 |
| M_HDL_CE | -0.0500 (-0.0600, -0.0100) | < 0.0001 |
| M_HDL_L | -0.0900 (-0.1300, -0.0500) | < 0.0001 |
| M_HDL_FC_pct | -0.0050 (-0.0080, 0.0010) | 0.059 |
| HDL_size | -0.0002 (-0.0040, 0.0070) | 0.660 |
| HDL_TG | -0.0002 (-0.0010, 0.0090) | 0.090 |
| HDL_PL | -0.0040 (-0.0080, -0.0020) | 0.027 |
| HDL_P | -0.0030 (-0.0050, 0.0050) | 0.170 |
| HDL_L | -0.0230 (-0.0370, -0.0090) | < 0.0001 |
| HDL_FC | -0.0130 (-0.0180, -0.0090) | < 0.0001 |
| HDL_CE | -0.0140 (-0.0180, -0.0090) | 0.001 |
| Glucose | -0.0030 (-0.0120, 0.0090) | 0.660 |
| bOHbutyrate | -0.0130 (-0.0230, -0.0030) | < 0.0001 |
| Pyruvate | 0.0660 (0.0540, 0.1800) | < 0.0001 |
| XS_VLDL_TG | 0.0130 (0.0090, 0.0150) | < 0.0001 |
| XS_VLDL_PL | -0.0012 (-0.0060, 0.0040) | 0.110 |
| XS_VLDL_P | -0.0014 (-0.0060, 0.0040) | 0.190 |
| XS_VLDL_L | -0.0020 (-0.0060, -0.0018) | < 0.0001 |
| XS_VLDL_FC | -0.0010 (-0.0060, -0.0008) | < 0.0001 |
| XS_VLDL_CE | -0.0018 (-0.0070, -0.0003) | < 0.0001 |
| XS_VLDL_C | -0.0016 (-0.0066, -0.0003) | 0.030 |
| XS_VLDL_C_pct | 0.0018 (-0.0016, 0.0050) | 0.304 |
| LDL_size | 0.0012 (-0.0090, 0.0090) | 0.645 |
| IDL_C | 0.0012 (0.0001, 0.0100) | 0.020 |
| IDL_CE | -0.0014 (-0.0030, 0.0060) | 0.587 |
| IDL_CE_pct | -0.0020 (-0.0030, 0.0080) | 0.521 |
| IDL_C_pct | -0.0010 (-0.0019, 0.0080) | 0.711 |
| IDL_FC | -0.0018 (-0.0020, 0.0080) | 0.487 |
| IDL_FC_pct | -0.0016 (-0.0017, 0.0080) | 0.546 |
| IDL_L | 0.0046 (-0.0030, 0.0060) | 0.060 |
| IDL_P | -0.0030 (-0.0130, -0.0010) | 0.010 |
| IDL_PL | 0.0039 (-0.0030, 0.0070) | 0.127 |
| IDL_PL_pct | -0.0130 (-0.0190, 0.0050) | 0.076 |
| IDL_TG | 0.0020 (-0.0140, 0.0040) | 0.175 |
| IDL_TG_pct | -0.0040 (-0.0080, 0.0015) | 0.070 |
| S_HDL_L | 0.0040 (0.0006, 0.0100) | 0.001 |
| S_HDL_P | 0.140 (0.008, 0.220) | 0.001 |
| S_HDL_PL | 0.0050 (0.0010, 0.0100) | 0.006 |
| S_HDL_PL_pct | 0.0010 (-0.0040, 0.0100) | 0.080 |
| S_HDL_FC_pct | 0.0080 (0.0020, 0.0090) | < 0.0001 |
| S_HDL_FC | 0.0130 (0.0020, 0.0220) | 0.001 |
| S_HDL_CE_pct | 0.0060 (0.0010, 0.0100) | 0.020 |
| S_HDL_CE | -0.0020 (-0.0120, 0.0080) | 0.310 |
| S_HDL_TG | 0.0030 (-0.0010, 0.0060) | 0.180 |
| S_HDL_TG_pct | 0.0030 (0.0010, 0.0100) | 0.006 |
| S_LDL_C | 0.0030 (0.0010, 0.0090) | < 0.0001 |
| S_LDL_CE | 0.0160 (0.0080, 0.0210) | < 0.0001 |
| S_LDL_CE_pct | 0.0230 (0.0150, 0.0330) | < 0.0001 |
| S_LDL_C_pct | 0.0310 (0.0190, 0.0410) | < 0.0001 |
| S_LDL_FC | -0.0040 (-0.0050, -0.0020) | 0.0006 |
| S_LDL_FC_pct | 0.0210 (0.0130, 0.0290) | 0.001 |
| S_LDL_L | 0.0050 (0.0030, 0.0060) | 0.020 |
| S_LDL_P | 0.0500 (-0.0200, 0.0800) | 0.170 |
| S_LDL_PL | 0.0040 (-0.0019, 0.0080) | 0.110 |
| S_LDL_PL_pct | 0.0040 (-0.0020, 0.0080) | 0.201 |
| S_LDL_TG | 0.0030 (0.0020, 0.0080) | 0.0003 |
| S_LDL_TG_pct | -0.0010 (-0.0030, 0.0060) | 0.051 |
| LDL_C | -0.0030 (-0.0130, -0.0010) | 0.0001 |
| LDL_CE | 0.0400 (-0.0030, 0.0700) | 0.060 |
| LDL_FC | 0.0200 (-0.0090, 0.0500) | 0.150 |
| LDL_L | -0.0040 (-0.0140, -0.0030) | 0.001 |
| LDL_P | -0.0030 (-0.0080, 0.0015) | 0.820 |
| LDL_PL | 0.0020 (0.0006, 0.0100) | 0.046 |
| LDL_TG | 0.0020 (-0.0080, 0.0030) | 0.088 |
| L_HDL_C_pct | -0.0018 (-0.0120, 0.0090) | 0.457 |
| L_HDL_TG_pct | -0.0110 (-0.0180, -0.0030) | < 0.0001 |
| L_LDL_FC_pct | 0.0230 (0.0110, 0.0560) | < 0.0001 |
| L_LDL_C_pct | 0.0039 (0.0010, 0.0090) | 0.030 |
| L_VLDL_CE_pct | -0.0200 (-0.0500, 0.0015) | 0.070 |
| L_VLDL_C_pct | -0.0010 (-0.0040, 0.0030) | 0.722 |
| MUFA | -0.0160 (-0.0180, -0.0110) | 0.003 |
| M_HDL_CE_pct | -0.0600 (-0.0700, -0.0090) | 0.007 |
| M_HDL_C_pct | -0.0900 (-0.1100, 0.0130) | 0.210 |
| M_HDL_TG_pct | -0.0110 (-0.0160, -0.0070) | < 0.0001 |
| Phosphatidylc | 0.0310 (0.0180, 0.0450) | < 0.0001 |
| Tyr | -0.370 (-0.590, -0.220) | 0.001 |
| XXL_VLDL_C_pct | -0.0050 (-0.0060, 0.0040) | 0.310 |
| XXL_VLDL_PL_pct | -0.0068 (-0.0100, -0.0030) | < 0.0001 |
| XXL_VLDL_CE_pct | -0.0069 (-0.0100, -0.0030) | < 0.0001 |
| XL_VLDL_PL | 0.0520 (0.0190, 0.0780) | < 0.0001 |

**Notes**: β coefficients represent the change in standardised metabolite concentration (z-score) per 10% increase in weight from ultra-processed food intake.

Models were adjusted for age, sex, ethnicity, marital status, employment grade, education level, smoking status, alcohol consumption, physical activity, total energy intake, and prevalent hypertension, dyslipidaemia, family history of cardiovascular disease, and family history of type 2 diabetes at baseline (phase 5, 1997–1999).

*P* values were adjusted for multiple testing using the Benjamini–Hochberg false discovery rate (FDR) correction.

Metabolites were quantified using high-throughput nuclear magnetic resonance (NMR) spectroscopy on the Nightingale Health platform (233 metabolites).

**Abbreviations**: Ala, alanine; Apo, apolipoprotein; β, regression coefficient; bOHbutyrate, β-hydroxybutyrate; C, cholesterol; CE, cholesteryl esters; CI, confidence interval; CRP, C-reactive protein; DHA, docosahexaenoic acid; FC, free cholesterol; FDR, false discovery rate; Gln, glutamine; Gly, glycine; GlycA, glycoprotein acetyls; HDL, high-density lipoprotein; IDL, intermediate-density lipoprotein; L, large; LA, linoleic acid; LDL, low-density lipoprotein; M, medium;

MPF, minimally processed foods; MUFA, monounsaturated fatty acids; P, particle concentration; pct, percentage of total lipids in lipoprotein subfraction; PG, phosphoglycerides; Phe, phenylalanine; Phosphatidylc, phosphatidylcholine; Phosphoglyc, phosphoglycerides; PL, phospholipids; PUFA, polyunsaturated fatty acids; S, small; SFA, saturated fatty acids; TG, triglycerides; Tyr, tyrosine; UPF, ultra-processed foods; VLDL, very low-density lipoprotein; XL, extra-large; XS, extra-small; XXL, extremely large.

Table S8. Cross-sectional associations between 233 metabolites and minimally processed food (MPF) intake in the Whitehall II cohort

| **Metabolite** | **β (95% CI)** | **P value** |
| --- | --- | --- |
| Acetate | 0.0000 (-0.0030, 0.0030) | 0.971 |
| Acetone | -0.0002 (-0.0006, 0.0001) | 0.263 |
| Acetoacetate | -0.0001 (-0.0020, 0.0020) | 0.922 |
| IDL_PL | -0.0002 (-0.0003, -0.0000) | 0.033 |
| IDL_L | -0.0008 (-0.0014, -0.0001) | 0.018 |
| HDL_C | -0.0020 (-0.0020, -0.0010) | < 0.0001 |
| Citrate | -0.0180 (-0.0210, -0.0090) | 0.002 |
| ApoB_by_ApoA1 | 0.0050 (0.0030, 0.0070) | 0.001 |
| Adiponectin | 0.0010 (-0.0020, 0.0020) | 0.900 |
| Sphingomyelins | -0.0060 (-0.0084, -0.0036) | < 0.0001 |
| CRP | -0.0050 (-0.0076, -0.0029) | < 0.0001 |
| Ala | -0.0010 (-0.0040, 0.0009) | 0.247 |
| Pyruvate | -0.0040 (-0.0064, -0.0016) | 0.001 |
| Lactate | -0.0028 (-0.0053, -0.0004) | 0.022 |
| Gly | -0.0009 (-0.0030, 0.0008) | 0.330 |
| Glycerol | -0.0075 (-0.0105, -0.0045) | < 0.0001 |
| Glucose | -0.0027 (-0.0051, -0.0003) | 0.027 |
| LA_pct | 0.0090 (0.0060, 0.0110) | < 0.0001 |
| LA | -0.0030 (-0.0050, 0.0001) | 0.060 |
| Gln | 0.0040 (0.0016, 0.0064) | 0.001 |
| XXL_VLDL_PL_pct | -0.0030 (-0.0055, -0.0005) | 0.020 |
| XXL_VLDL_L | -0.0054 (-0.0078, -0.0030) | < 0.0001 |
| Creatinine | 0.0043 (-0.0047, 0.0069) | 0.310 |
| XXL_VLDL_FC_pct | -0.0070 (-0.0100, 0.0020) | 0.520 |
| XXL_VLDL_FC | -0.0040 (-0.0058, -0.0022) | < 0.0001 |
| XXL_VLDL_P | -0.0040 (-0.0060, -0.0020) | 0.001 |
| XXL_VLDL_C_pct | 0.0040 (0.0016, 0.0064) | 0.001 |
| XXL_VLDL_CE_pct | -0.0050 (-0.0068, -0.0032) | < 0.0001 |
| XXL_VLDL_PL | -0.0040 (-0.0060, -0.0020) | 0.001 |
| XXL_VLDL_CE | 0.0040 (0.0020, 0.0060) | 0.0001 |
| XXL_VLDL_TG | -0.0030 (-0.0060, -0.0010) | 0.003 |
| XXL_VLDL_C | -0.0050 (-0.0072, -0.0028) | < 0.0001 |
| XS_VLDL_TG_pct | -0.0040 (-0.0064, -0.0016) | 0.001 |
| XS_VLDL_TG | -0.0050 (-0.0070, -0.0030) | < 0.0001 |
| XS_VLDL_PL_pct | -0.0030 (-0.0051, -0.0009) | 0.006 |
| XS_VLDL_PL | -0.0040 (-0.0064, -0.0016) | 0.001 |
| XS_VLDL_P | -0.0040 (-0.0068, -0.0012) | 0.005 |
| XS_VLDL_L | -0.0040 (-0.0065, -0.0015) | 0.002 |
| XS_VLDL_FC_pct | 0.0050 (0.0030, 0.0070) | < 0.0001 |
| XS_VLDL_FC | -0.0032 (-0.0056, -0.0008) | 0.010 |
| XS_VLDL_C_pct | 0.0044 (0.0026, 0.0062) | < 0.0001 |
| XS_VLDL_CE_pct | 0.0042 (0.0017, 0.0067) | 0.001 |
| XS_VLDL_C | -0.0027 (-0.0052, -0.0002) | 0.032 |
| M_LDL_C | -0.0010 (-0.0040, 0.0090) | 0.230 |
| L_VLDL_C | -0.0020 (-0.0040, 0.0007) | 0.170 |
| L_VLDL_TG_pct | -0.0010 (-0.0030, 0.0010) | 0.390 |
| L_VLDL_FC_pct | 0.0008 (-0.0020, 0.0030) | 0.500 |
| L_VLDL_C_pct | 0.0020 (-0.0006, 0.0040) | 0.150 |
| L_VLDL_CE_pct | 0.0020 (-0.0001, 0.0050) | 0.060 |
| XL_VLDL_TG | -0.0041 (-0.0065, -0.0017) | 0.001 |
| XL_VLDL_PL | -0.0042 (-0.0067, -0.0017) | 0.001 |
| XL_VLDL_P | -0.0050 (-0.0067, -0.0033) | < 0.0001 |
| XL_VLDL_L | -0.0047 (-0.0068, -0.0026) | < 0.0001 |
| XL_VLDL_FC | -0.0043 (-0.0058, -0.0027) | < 0.0001 |
| XL_VLDL_C_pct | 0.0047 (0.0026, 0.0068) | < 0.0001 |
| XL_VLDL_CE_pct | 0.0051 (0.0030, 0.0071) | < 0.0001 |
| XL_VLDL_CE | -0.0040 (-0.0066, -0.0014) | 0.003 |
| XL_VLDL_C | -0.0040 (-0.0064, -0.0016) | 0.001 |
| XL_HDL_TG_pct | -0.0060 (-0.0087, -0.0033) | < 0.0001 |
| XL_HDL_TG | -0.0040 (-0.0065, -0.0015) | 0.002 |
| XL_HDL_PL | 0.0030 (0.0005, 0.0055) | 0.020 |
| XL_HDL_P | 0.0030 (0.0005, 0.0055) | 0.020 |
| XL_HDL_L | 0.0030 (0.0008, 0.0052) | 0.007 |
| XL_HDL_FC | 0.0040 (0.0025, 0.0055) | < 0.0001 |
| XL_HDL_CE | 0.0035 (0.0012, 0.0058) | 0.003 |
| XL_HDL_C | 0.0040 (0.0016, 0.0064) | 0.001 |
| VLDL_size | -0.0045 (-0.0063, -0.0027) | < 0.0001 |
| VLDL_TG | -0.0050 (-0.0068, -0.0032) | < 0.0001 |
| VLDL_PL | -0.0043 (-0.0069, -0.0017) | 0.001 |
| VLDL_P | -0.0040 (-0.0065, -0.0015) | 0.002 |
| VLDL_L | -0.0050 (-0.0070, -0.0030) | < 0.0001 |
| VLDL_FC | -0.0040 (-0.0065, -0.0015) | 0.002 |
| VLDL_CE | -0.0030 (-0.0055, -0.0005) | 0.020 |
| VLDL_C | -0.0033 (-0.0057, -0.0009) | 0.007 |
| Unsaturation | 0.0087 (0.0055, 0.0119) | < 0.0001 |
| TG_by_PG | -0.0040 (-0.0064, -0.0016) | 0.001 |
| S_VLDL_TG_pct | -0.0030 (-0.0054, -0.0006) | 0.013 |
| S_VLDL_TG | -0.0045 (-0.0062, -0.0028) | < 0.0001 |
| S_VLDL_PL_pct | 0.0040 (0.0016, 0.0064) | 0.001 |
| S_VLDL_PL | -0.0028 (-0.0052, -0.0004) | 0.024 |
| S_VLDL_P | -0.0040 (-0.0066, -0.0014) | 0.003 |
| S_VLDL_L | -0.0037 (-0.0061, -0.0013) | 0.003 |
| S_VLDL_FC_pct | 0.0039 (0.0014, 0.0064) | 0.002 |
| S_VLDL_C_pct | 0.0030 (0.0006, 0.0054) | 0.015 |
| S_VLDL_CE | -0.0030 (-0.0056, -0.0004) | 0.024 |
| S_VLDL_C | -0.0030 (-0.0059, -0.0001) | 0.040 |
| S_LDL_TG_pct | -0.0050 (-0.0067, -0.0033) | < 0.0001 |
| S_LDL_CE | -0.0010 (-0.0030, 0.0010) | 0.380 |
| S_LDL_C | -0.0049 (-0.0071, -0.0027) | < 0.0001 |
| S_LDL_C_pct | -0.0010 (-0.0040, 0.0010) | 0.250 |
| S_LDL_L | 0.0001 (-0.0020, 0.0020) | 0.930 |
| S_LDL_P | 0.0006 (-0.0022, 0.0020) | 0.960 |
| S_LDL_PL | 0.0020 (-0.0005, 0.0040) | 0.120 |
| S_LDL_TG | -0.0051 (-0.0071, -0.0031) | < 0.0001 |
| S_LDL_FC | 0.0020 (0.0008, 0.0050) | 0.049 |
| S_LDL_PL_pct | 0.0080 (0.0048, 0.0112) | < 0.0001 |
| S_LDL_FC_pct | 0.0044 (0.0024, 0.0064) | < 0.0001 |
| S_LDL_CE_pct | -0.0080 (-0.0112, -0.0048) | < 0.0001 |
| S_HDL_TG_pct | -0.0042 (-0.0063, -0.0021) | 0.0001 |
| S_HDL_TG | -0.0070 (-0.0101, -0.0039) | < 0.0001 |
| S_HDL_PL_pct | -0.0050 (-0.0075, -0.0025) | 0.0001 |
| S_HDL_PL | -0.0096 (-0.0139, -0.0053) | < 0.0001 |
| S_HDL_P | -0.0076 (-0.0110, -0.0042) | < 0.0001 |
| S_HDL_L | -0.0094 (-0.0132, -0.0056) | < 0.0001 |
| S_HDL_FC_pct | 0.0046 (0.0029, 0.0063) | < 0.0001 |
| S_HDL_FC | -0.0076 (-0.0106, -0.0046) | < 0.0001 |
| S_HDL_C_pct | 0.0057 (0.0034, 0.0080) | < 0.0001 |
| S_HDL_CE_pct | 0.0047 (0.0028, 0.0066) | < 0.0001 |
| S_HDL_CE | -0.0061 (-0.0085, -0.0037) | < 0.0001 |
| XL_HDL_PL_pct | 0.0020 (-0.0010, 0.0040) | 0.160 |
| M_VLDL_C_pct | 0.0040 (0.0010, 0.0060) | 0.002 |
| M_HDL_FC_pct | -0.0020 (-0.0050, -0.0003) | 0.030 |
| S_HDL_C | -0.0069 (-0.0094, -0.0044) | < 0.0001 |
| SFA_pct | -0.0086 (-0.0120, -0.0052) | < 0.0001 |
| SFA | -0.0068 (-0.0098, -0.0038) | < 0.0001 |
| Phosphoglyc | -0.0056 (-0.0080, -0.0031) | < 0.0001 |
| Phosphatidylc | -0.0050 (-0.0070, -0.0030) | < 0.0001 |
| PUFA | -0.0300 (-0.0500, -0.0100) | 0.0001 |
| PUFA_pct | 0.0098 (0.0059, 0.0137) | < 0.0001 |
| PUFA_by_MUFA | 0.0088 (0.0056, 0.0120) | < 0.0001 |
| Omega_6_pct | 0.0060 (0.0033, 0.0087) | < 0.0001 |
| Omega_6_by_Omega_3 | -0.0062 (-0.0089, -0.0035) | < 0.0001 |
| Omega_6 | -0.0036 (-0.0060, -0.0011) | 0.004 |
| Omega_3_pct | 0.0082 (0.0046, 0.0118) | < 0.0001 |
| Omega_3 | 0.0042 (0.0021, 0.0063) | 0.0001 |
| XS_VLDL_CE | -0.0030 (-0.0050, -0.0007) | 0.010 |
| M_VLDL_TG_pct | -0.0034 (-0.0058, -0.0010) | 0.005 |
| M_VLDL_TG | -0.0041 (-0.0065, -0.0017) | 0.001 |
| M_VLDL_PL | -0.0030 (-0.0055, -0.0005) | 0.018 |
| M_VLDL_P | -0.0030 (-0.0054, -0.0006) | 0.015 |
| M_VLDL_L | -0.0030 (-0.0053, -0.0008) | 0.009 |
| M_VLDL_FC | -0.0010 (-0.0040, 0.0010) | 0.270 |
| M_VLDL_FC_pct | 0.0020 (-0.0003, 0.0040) | 0.099 |
| M_VLDL_PL_pct | 0.0020 (-0.0001, 0.0040) | 0.170 |
| M_VLDL_CE_pct | 0.0030 (0.0007, 0.0053) | 0.011 |
| M_LDL_TG_pct | -0.0037 (-0.0061, -0.0013) | 0.003 |
| M_LDL_TG | -0.0053 (-0.0076, -0.0029) | < 0.0001 |
| M_LDL_PL_pct | 0.0048 (0.0027, 0.0069) | < 0.0001 |
| M_LDL_FC_pct | 0.0040 (0.0015, 0.0065) | 0.002 |
| M_LDL_CE_pct | -0.0045 (-0.0065, -0.0025) | < 0.0001 |
| M_LDL_C_pct | 0.0030 (0.0004, 0.0050) | 0.020 |
| M_LDL_CE | -0.0027 (-0.0051, -0.0003) | 0.030 |
| M_HDL_TG_pct | -0.0054 (-0.0076, -0.0032) | < 0.0001 |
| M_HDL_TG | -0.0078 (-0.0109, -0.0047) | < 0.0001 |
| M_HDL_PL_pct | -0.0051 (-0.0074, -0.0028) | < 0.0001 |
| M_HDL_PL | -0.0066 (-0.0092, -0.0040) | < 0.0001 |
| M_HDL_P | -0.0053 (-0.0076, -0.0029) | < 0.0001 |
| M_HDL_L | -0.0059 (-0.0083, -0.0036) | < 0.0001 |
| M_HDL_FC | -0.0048 (-0.0069, -0.0027) | < 0.0001 |
| M_HDL_C_pct | 0.0053 (0.0032, 0.0074) | < 0.0001 |
| M_HDL_CE_pct | 0.0056 (0.0031, 0.0080) | < 0.0001 |
| M_HDL_CE | -0.0039 (-0.0062, -0.0016) | 0.001 |
| M_HDL_C | -0.0041 (-0.0059, -0.0023) | < 0.0001 |
| MUFA_pct | -0.0074 (-0.0107, -0.0041) | < 0.0001 |
| MUFA | -0.0067 (-0.0097, -0.0037) | < 0.0001 |
| L_VLDL_TG | -0.0041 (-0.0065, -0.0017) | 0.001 |
| L_VLDL_PL_pct | -0.0020 (-0.0044, 0.0004) | 0.099 |
| L_VLDL_PL | -0.0037 (-0.0060, -0.0014) | 0.002 |
| L_VLDL_P | -0.0045 (-0.0065, -0.0025) | < 0.0001 |
| L_VLDL_L | -0.0043 (-0.0062, -0.0024) | < 0.0001 |
| L_VLDL_FC | -0.0032 (-0.0056, -0.0008) | 0.009 |
| L_VLDL_CE | -0.0030 (-0.0054, -0.0006) | 0.013 |
| L_LDL_TG_pct | -0.0042 (-0.0067, -0.0017) | 0.001 |
| L_LDL_PL | -0.0020 (-0.0040, 0.0004) | 0.110 |
| L_LDL_TG | -0.0057 (-0.0082, -0.0032) | < 0.0001 |
| L_LDL_PL_pct | 0.0024 (0.0001, 0.0047) | 0.042 |
| L_LDL_FC_pct | 0.0051 (0.0028, 0.0074) | < 0.0001 |
| L_LDL_C | -0.0050 (-0.0120, 0.0001) | 0.090 |
| L_LDL_C_pct | 0.0031 (0.0007, 0.0055) | 0.013 |
| L_HDL_TG_pct | -0.0052 (-0.0075, -0.0029) | < 0.0001 |
| L_HDL_TG | -0.0053 (-0.0076, -0.0029) | < 0.0001 |
| L_HDL_PL_pct | -0.0065 (-0.0091, -0.0039) | < 0.0001 |
| L_HDL_C_pct | 0.0059 (0.0033, 0.0085) | < 0.0001 |
| L_HDL_CE_pct | 0.0060 (0.0033, 0.0087) | < 0.0001 |
| LDL_L | -0.0013 (-0.0021, 0.0000) | 0.082 |
| LDL_FC | -0.0001 (-0.0003, 0.0002) | 0.704 |
| LDL_CE | -0.0007 (-0.0015, 0.0000) | 0.062 |
| LDL_size | 0.0001 (0.0000, 0.0002) | 0.040 |
| L_HDL_PL | -0.0040 (-0.0070, -0.0020) | 0.001 |
| L_HDL_P | -0.0030 (-0.0050, -0.0010) | 0.003 |
| L_HDL_L | -0.0040 (-0.0060, -0.0020) | 0.0001 |
| L_HDL_CE | -0.0020 (-0.0040, 0.0001) | 0.120 |
| L_HDL_C | -0.0020 (-0.0040, 0.0002) | 0.070 |
| LDL_PL | -0.0020 (-0.0050, 0.0000) | 0.210 |
| LDL_P | -0.0090 (-0.0180, 0.0060) | 0.780 |
| L_LDL_L | -0.0090 (-0.0100, -0.0003) | < 0.0001 |
| L_LDL_FC | -0.0080 (-0.0090, 0.0009) | 0.380 |
| L_LDL_CE_pct | -0.0001 (-0.0020, 0.0020) | 0.910 |
| L_LDL_CE | -0.0050 (-0.0100, 0.0000) | 0.330 |
| LDL_C | -0.0007 (-0.0020, 0.0002) | 0.140 |
| LDL_TG | -0.0060 (-0.0084, -0.0036) | < 0.0001 |
| IDL_TG_pct | -0.0050 (-0.0072, -0.0028) | < 0.0001 |
| IDL_P | -0.0007 (-0.0010, 0.0008) | 0.365 |
| IDL_CE | -0.0003 (-0.0007, 0.0000) | 0.064 |
| IDL_TG | -0.0056 (-0.0081, -0.0031) | < 0.0001 |
| IDL_FC | -0.0000 (-0.0000, 0.0001) | 0.306 |
| IDL_PL_pct | 0.0010 (-0.0010, 0.0030) | 0.365 |
| IDL_FC_pct | 0.0042 (0.0017, 0.0067) | 0.001 |
| IDL_C | -0.0004 (-0.0008, 0.0001) | 0.103 |
| IDL_C_pct | 0.0051 (0.0025, 0.0077) | 0.0001 |
| IDL_CE_pct | 0.0048 (0.0027, 0.0069) | < 0.0001 |
| HDL_TG | -0.0073 (-0.0105, -0.0041) | < 0.0001 |
| HDL_PL | -0.0046 (-0.0064, -0.0028) | < 0.0001 |
| HDL_P | -0.0058 (-0.0084, -0.0032) | < 0.0001 |
| HDL_size | -0.0010 (-0.0030, 0.0009) | 0.220 |
| HDL_CE | -0.0060 (-0.0090, -0.0004) | 0.010 |
| HDL_L | -0.0038 (-0.0055, -0.0021) | < 0.0001 |
| HDL_FC | -0.0025 (-0.0047, -0.0003) | 0.024 |
| GlycA | -0.0056 (-0.0081, -0.0031) | < 0.0001 |
| DHA_pct | 0.0104 (0.0058, 0.0150) | < 0.0001 |
| DHA | 0.0068 (0.0038, 0.0098) | < 0.0001 |
| Cholines | -0.0049 (-0.0069, -0.0029) | < 0.0001 |
| ApoA1 | -0.0050 (-0.0070, -0.0030) | < 0.0001 |
| Phe | -0.0003 (-0.0030, 0.0020) | 0.781 |
| L_HDL_FC_pct | -0.0070 (-0.0105, -0.0035) | 0.0001 |
| L_LDL_P | -0.0027 (-0.0050, -0.0004) | 0.020 |
| M_LDL_FC | -0.0026 (-0.0050, -0.0002) | 0.037 |
| M_LDL_L | -0.0025 (-0.0049, 0.0091) | 0.052 |
| M_LDL_PL | -0.0054 (-0.0074, -0.0034) | < 0.0001 |
| M_LDL_P | -0.0056 (-0.0078, 0.0034) | 0.310 |
| M_VLDL_C | -0.0027 (-0.0051, -0.0003) | 0.030 |
| M_VLDL_CE | -0.0048 (-0.0066, -0.0030) | < 0.0001 |
| S_VLDL_CE_pct | -0.0090 (-0.0110, 0.0060) | 0.330 |
| S_VLDL_FC | -0.0045 (-0.0065, -0.0025) | < 0.0001 |
| XL_HDL_FC_pct | -0.0055 (-0.0077, -0.0033) | < 0.0001 |
| XL_VLDL_PL_pct | -0.0056 (-0.0076, -0.0035) | < 0.0001 |
| XL_HDL_CE_pct | -0.0060 (-0.0084, -0.0036) | < 0.0001 |
| XXL_VLDL_TG_pct | -0.0020 (-0.0050, 0.0001) | 0.065 |
| bOHbutyrate | -0.0026 (-0.0048, -0.0004) | 0.023 |
| Tyr | 0.0048 (0.0024, 0.0072) | 0.0001 |
| XL_HDL_C_pct | -0.0028 (-0.0052, -0.0004) | 0.023 |
| ApoB | -0.0033 (-0.0057, 0.0095) | 0.210 |
| L_HDL_FC | 0.0280 (0.0210, 0.0360) | < 0.0001 |

**Notes**: β coefficients represent the change in standardised metabolite concentration (z-score) per 10% increase in weight from minimally processed food intake.

Models were adjusted for age, sex, ethnicity, marital status, employment grade, education level, smoking status, alcohol consumption, physical activity, total energy intake, and prevalent hypertension, dyslipidaemia, family history of cardiovascular disease, and family history of type 2 diabetes at baseline (phase 5, 1997–1999).

*P* values were adjusted for multiple testing using the Benjamini–Hochberg false discovery rate (FDR) correction.

Metabolites were quantified using high-throughput nuclear magnetic resonance (NMR) spectroscopy on the Nightingale Health platform (233 metabolites).

**Abbreviations**: Ala, alanine; Apo, apolipoprotein; β, regression coefficient; bOHbutyrate, β-hydroxybutyrate; C, cholesterol; CE, cholesteryl esters; CI, confidence interval; CRP, C-reactive protein; DHA, docosahexaenoic acid; FC, free cholesterol; FDR, false discovery rate; Gln, glutamine; Gly, glycine; GlycA, glycoprotein acetyls; HDL, high-density lipoprotein; IDL, intermediate-density lipoprotein; L, large; LA, linoleic acid; LDL, low-density lipoprotein; M, medium;

MPF, minimally processed foods; MUFA, monounsaturated fatty acids; P, particle concentration; pct, percentage of total lipids in lipoprotein subfraction; PG, phosphoglycerides; Phe, phenylalanine; Phosphatidylc, phosphatidylcholine; Phosphoglyc, phosphoglycerides; PL, phospholipids; PUFA, polyunsaturated fatty acids; S, small; SFA, saturated fatty acids; TG, triglycerides; Tyr, tyrosine; UPF, ultra-processed foods; VLDL, very low-density lipoprotein; XL, extra-large; XS, extra-small; XXL, extremely large.

Table S9. Comparison of characteristics between training (N=4,207) and testing (N=1,803) sets used for metabolomic signature derivation

| **Characteristic** | **Training set (n = 4207)** | **Test set (n = 1803)** |
| --- | --- | --- |
| **Exposure** | | |
| UPF intake (% g/day), median (IQR) | 18.4 (14.0-23.6) | 18.7 (14.4-23.7) |
| MPF intake (% g/day), median (IQR) | 62.1 (54.0-69.3) | 62.4 (54.6-69.0) |
| **Demographics** | | |
| Age (years), median (IQR) | 55.1 (50.6-61.0) | 55.0 (50.6-61.0) |
| Sex, n (%) men | 3215 (76.4) | 1388 (77.0) |
| Ethnicity, n (%) white | 3816 (90.7) | 1641 (91.0) |
| Employment grade, n (%) |  |  |
| *High* | 1614 (38.4) | 687 (38.1) |
| *Intermediate* | 1948 (46.3) | 843 (46.8) |
| *Low* | 645 (15.3) | 273 (15.1) |
| **Lifestyle** | | |
| Smoking status, n (%) | | |
| *Current* | 389 (9.2) | 163 (9.0) |
| *Former smoker* | 1569 (37.3) | 679 (37.7) |
| *Never smoker* | 2238 (53.2) | 961 (53.3) |
| Alcohol consumption, n (%) | | |
| *Abstainers* | 703 (16.7) | 308 (17.1) |
| *Moderate drinkers* | 2015 (47.9) | 869 (48.2) |
| *Heavy drinkers* | 1489 (35.4) | 626 (34.7) |
| Physical activity, n (%) | | |
| *Inactive* | 1573 (37.4) | 676 (37.5) |
| *Moderately Active* | 749 (17.8) | 318 (17.6) |
| *Active* | 1885 (44.8) | 809 (44.9) |
| Total energy intake (kcal/day), median (IQR) | 2150.7 (1765-2576) | 2153.1 (1764-2594) |
| **Clinical** | | |
| BMI (kg/m²), median (IQR) | 25.6 (23.6-28.0) | 25.7 (23.5-28.2) |
| **Outcomes** | | |
| Incident CVD, n (%) | 764 (18.2) | 330 (18.3) |
| Incident T2D, n (%) | 594 (14.1) | 249 (14.4) |

**Abbreviations:** UPF, ultra-processed food; MPF, minimally processed food; CVD, cardiovascular disease; T2D, type 2 diabetes; IQR, interquartile range.

Notes: The analytic sample was randomly split into training (70%) and test (30%) sets.

No formal significance tests are reported as the train/test split was random by design.

Values are n (%); for incident outcomes, n is the number of events.

Table S10. Stratified analyses: associations of UPF and MPF intake and their metabolomic signatures (per 1-SD) with incident CVD and T2D in the Whitehall II cohort (N=6,010)

|  | N | Incident CVD | | | | | | | Incident T2D | | | | | | |
| --- | --- | --- | --- | --- | --- | --- | --- | --- | --- | --- | --- | --- | --- | --- | --- |
|  |  | Events | Dietary intake | | | Metabolomic signature | | | Events | Dietary intake | | Metabolomic signature | | | |
|  |  |  | UPF  HR (95% CI) | MPF  HR (95% CI) | P int | UPF  HR (95% CI) | MPF  HR (95% CI) | P int |  | UPF  HR (95% CI) | MPF HR (95% CI) | P int | UPF HR (95% CI) | MPF HR (95% CI) | P int |
| **Smoking Status** | | | | | 0.42 |  | | 0.17 |  | | | 0.36 |  |  | 0.14 |
| Never | 3,210 | 489 | 1.10 (1.01–1.25) | 0.80 (0.71–0.88) |  | 1.14 (1.02–1.35) | 0.79 (0.74–0.86) |  | 378 | 1.13 (1.04–1.24) | 0.79 (0.66–0.91) |  | 1.12 (1.04–1.29) | 0.81 (0.72–0.91) |  |
| Former | 2,248 | 468 | 1.16 (1.03–1.32) | 0.88 (0.82–1.02) |  | 1.02 (0.97–1.18) | 0.85 (0.79–0.94) |  | 365 | 1.09 (0.98–1.19) | 0.89 (0.81–1.06) |  | 1.15 (1.09–1.38) | 0.82 (0.76–0.93) |  |
| Current | 552 | 137 | 1.05 (0.90–1.17) | 0.91 (0.86–1.09) |  | 1.03 (0.98–1.19) | 0.95 (0.90–1.09) |  | 110 | 1.08 (0.94–1.17) | 0.92 (0.88–1.12) |  | 1.09 (0.92–1.26) | 0.93 (0.85–1.09) |  |
| **Sex** | | | | | 0.16 |  | | 0.06 |  | | | 0.17 |  |  | 0.09 |
| Male | 4,603 | 826 | 1.18 (1.03–1.27) | 0.92 (0.86–0.98) |  | 1.22 (1.10–1.37) | 0.92 (0.86–1.05) |  | 598 | 1.21 (1.10–1.27) | 0.87 (0.76–0.97) |  | 1.22 (1.09–1.36) | 0.77 (0.71–0.84) |  |
| Female | 1,407 | 268 | 1.07 (0.95–1.20) | 0.69 (0.56–0.88) |  | 1.18 (1.05–1.31) | 0.74 (0.61–0.87) |  | 255 | 1.02 (0.94–1.15) | 0.73 (0.68–0.89) |  | 1.16 (1.03–1.29) | 0.75 (0.69–0.88) |  |
| **BMI** | | | | | 0.09 |  | | 0.14 |  |  |  | 0.26 |  |  | 0.15 |
| < 25 kg/m² | 2,489 | 404 | 1.25 (1.10–1.42) | 0.96 (0.84–1.08) |  | 1.20 (1.09–1.36) | 0.97 (0.84–1.08) |  | 207 | 1.03 (0.95–1.19) | 0.88 (0.79–0.97) |  | 1.23 (1.11–1.46) | 0.76 (0.65–0.88) |  |
| 25–29.9 kg/m² | 2,656 | 437 | 1.18 (1.05–1.32) | 0.88 (0.79–0.97) |  | 1.11 (0.95–1.29) | 0.88 (0.79–0.97) |  | 405 | 1.20 (1.10–1.32) | 0.83 (0.74–0.90) |  | 1.09 (0.97–1.20) | 0.79 (0.64–1.15) |  |
| ≥30 kg/m² | 865 | 253 | 1.08 (0.98–1.28) | 0.83 (0.75–0.93) |  | 1.19 (1.07–1.28) | 0.81 (0.74–0.92) |  | 241 | 1.08 (0.98–1.28) | 0.96 (0.84–1.08) |  | 1.26 (1.13–1.48) | 0.84 (0.76–1.13) |  |

Abbreviations: CVD, Cardiovascular diseases; T2D, Type 2 diabetes; HR, Hazard ratio; CI, Confidence interval, UPF, Ultra-processed food; MPF, minimally processed foods. BMI, body mass index.

Models were adjusted for age at baseline, sex, ethnicity, education, employment grade, marital status, physical activity, alcohol consumption, smoking status, family history of CVD/T2D, total energy intake, diet quality indicators (total sugar, sodium, total fat, fibre) and clinical measures.

Table S11**.** Associations between UPF subgroup intake (per 1-SD increment, %g/day) and incident CVD and T2D in the Whitehall II cohort (N=6,010)

| **UPF subgroup** | Contribution to total UPF intake (%) | CVD  HR (95% CI) | T2D  HR (95% CI) |
| --- | --- | --- | --- |
| Drinks | 21.1 | 1.08 (1.02-1.15) | 1.13 (1.02-1.24) |
| Dairy Products | 5.5 | 0.97 (0.89-1.16) | 1.04 (0.97-1.18) |
| Sauces and spreads | 1.2 | 0.93 (0.87-1.10) | 0.89 (0.81-1.10) |
| Meats | 20.4 | 1.17 (1.04-1.26) | 1.18 (1.03-1.30) |
| Ready to eat meals | 6.4 | 1.01 (0.97-1.10) | 0.92 (0.85-1.05) |
| Sweets | 15.0 | 1.07 (0.98-1.16) | 1.06 (0.96-1.15) |
| Savoury snacks | 7.6 | 1.02 (0.97-1.12) | 0.94 (0.89-1.13) |
| Cereals | 14.6 | 0.98 (0.93-1.08) | 1.02 (0.94-1.20) |
| Breads | 8.2 | 0.94 (0.89-1.05) | 0.85 (0.79-0.92) |

Abbreviations: UPF, ultra-processed food; CVD, cardiovascular disease; T2D, type 2 diabetes; HR, hazard ratio; CI, confidence interval; SD, standard deviation.

Models adjusted for age at baseline, sex, ethnicity, education, employment grade, marital status, physical activity, alcohol consumption, smoking status, family history of CVD/T2D, total energy intake, BMI, hypertension, and dyslipidaemia, diet quality indicators (total sugar, sodium, total fat, fibre) and clinical measures.

Each UPF subgroup was modelled separately as a continuous variable (per 1 SD increment, %g/day).

Table S12. Sensitivity analysis using multiple imputation by chained equations (MICE): prospective associations of tertiles of UPF and MPF intake and metabolomic signature scores with incident CVD and T2D in the Whitehall II cohort (N=6,010).

| Exposure | Tertile 1 (low) | Tertile 2 | Tertile 3 (high) | P trend | Per 1-SD HR (95% CI) |
| --- | --- | --- | --- | --- | --- |
| Participants, n | 2,003 | 2,003 | 2,004 |  |  |
| CVD (1,094 cases) | | | | | |
| UPF intake | Reference | 1.14 (0.97–1.29) | 1.24 (1.07–1.38) | 0.015 | 1.18 (1.04–1.31) |
| MPF intake | Reference | 0.93 (0.77–1.08) | 0.84 (0.73–0.94) | 0.035 | 0.86 (0.76–0.96) |
| UPF metabolic signature | Reference | 1.09 (0.96–1.25) | 1.19 (1.05–1.36) | 0.033 | 1.16 (1.03-1.35) |
| MPF metabolic signature | Reference | 0.95 (0.86–1.10) | 0.83 (0.74–0.94) | 0.027 | 0.87 (0.79-0.97) |
| T2D (853 cases) | | | | | |
| UPF intake | Reference | 1.02 (0.88–1.20) | 1.19 (1.04–1.31) | 0.008 | 1.17 (1.03–1.28) |
| MPF intake | Reference | 0.87 (0.76–1.04) | 0.85 (0.74–0.96) | 0.027 | 0.86 (0.77–0.97) |
| UPF metabolic signature | Reference | 1.01 (0.88–1.17) | 1.21 (1.03–1.42) | 0.029 | 1.18 (1.06-1.39) |
| MPF metabolic signature | Reference | 0.80 (0.69–0.97) | 0.61 (0.48–0.73) | 3.8×10^−7^ | 0.68 (0.53-0.79) |

Abbreviations: CI, confidence interval; CVD, cardiovascular disease; HR, hazard ratio; MICE, multiple imputation by chained equations; MPF, minimally processed foods; SD, standard deviation; T2D, type 2 diabetes; UPF, ultra-processed foods.

Models adjusted for age at baseline, sex, ethnicity, education, employment grade, marital status, physical activity, alcohol consumption, smoking status, family history of CVD (T2D models: family history of T2D), total energy intake, BMI, hypertension, dyslipidaemia, and indicators of diet quality (total sugar, sodium, total fat, fibre).

P trend was calculated treating the categorical variable as continuous using the median value within each category. Hazard ratios per 1-SD increment were estimated from separate Cox models fitted with the exposure on a continuous z-score scale.

Table S13. Complete-case sensitivity analysis: associations between UPF/MPF metabolomic signature scores and risk of CVD in the Whitehall II cohort (N=4,551; 998 CVD events).

| Cases | Tertile 1 (reference) | Tertile 2 (HR,95%CI) | Tertile 3 (HR,95%CI) | Per 1 SD (HR,95%CI) | P for trend |
| --- | --- | --- | --- | --- | --- |
| **Panel A: Dietary intake** | | | | | |
| Cases | 316 | 332 | 350 | 998 |  |
| UPF intake (%/day) | Reference | 1.10 (0.93-1.31) | 1.24 (1.08-1.46) | 1.19 (1.03-1.38) | 0.033 |
| Cases | 366 | 331 | 301 | 998 |  |
| MPF intake (%/day) | Reference | 0.95 (0.81-1.12) | 0.85 (0.75-0.94) | 0.88 (0.79-0.97) | 0.042 |
| **Panel B: Metabolic signature** | | | | | |
| Cases | 293 | 346 | 359 | 998 |  |
| UPF metabolic signature | Reference | 1.09 (0.89-1.28) | 1.21 (1.03-1.41) | 1.17 (1.02-1.33) | 0.027 |
| Cases | 365 | 332 | 301 | 998 |  |
| MPF metabolic signature | Reference | 0.94 (0.81-1.08) | 0.82 (0.72-0.94) | 0.84 (0.75-0.90) | 0.031 |

Abbreviations: T2D, Type 2 diabetes; HR, Hazard ratio; CI, Confidence interval, UPF, Ultra-processed food; MPF, Minimally processed food.

Models were adjusted for age at baseline, sex, ethnicity, education, employment grade, marital status, physical activity, alcohol consumption, smoking status, family history of CVD/T2D, total energy intake, diet quality indicators (total sugar, sodium, total fat, fibre) and clinical measures.

Table S14. Complete-case sensitivity analysis: associations between UPF/MPF metabolomic signature scores and risk of T2D in the Whitehall II cohort (N=4,551; 742 T2D events)

|  | Tertile 1 (reference) | Tertile 2 (HR, 95%CI) | Tertile 3 (HR, 95%CI) | Per 1 SD (HR, 95%CI) | P for trend |
| --- | --- | --- | --- | --- | --- |
| **Panel A: Dietary intake** | | | | | |
| Cases/ N | 204 | 232 | 306 | 742 |  |
| UPF intake (%/day) | Reference | 1.04 (0.87-1.25) | 1.22 (1.08-1.38) | 1.16 (1.04-1.29) | 0.031 |
| Cases/ N | 276 | 245 | 221 | 742 |  |
| MPF intake (%/day) | Reference | 0.95 (0.81-1.12) | 0.81 (0.75-0.94) | 0.84 (0.79-0.91) | 0.028 |
| **Panel B: Metabolic signature** | | | | | |
| Cases/N | 208 | 246 | 288 | 742 |  |
| UPF metabolic signature score | Reference | 1.01 (0.79-1.15) | 1.25 (1.10-1.42) | 1.21 (1.05-1.36) | 0.033 |
| Cases/N | 277 | 253 | 212 | 742 |  |
| MPF metabolic signature scores | Reference | 0.74(0.59-0.87) | 0.65(0.48-0.83) | 0.77 (0.69-0.86) | 1.70×10^-6^ |

Abbreviations: T2D, Type 2 diabetes; HR, Hazard ratio; CI, Confidence interval, UPF, Ultra-processed food; MPF, minimally processed food.

Models were adjusted for age at baseline, sex, ethnicity, education, employment grade, marital status, physical activity, alcohol consumption, smoking status, family history of CVD/T2D, total energy intake, diet quality indicators (total sugar, sodium, total fat, fibre) and clinical measures.

Table S15. Two-year lag sensitivity analysis: associations of UPF intake, MPF intake and metabolomic signature scores with incident CVD in the Whitehall II cohort (N=5,970; CVD events=1,008)

|  | Tertile 1 (Ref) | Tertile 2 (HR, 95% CI) | Tertile 3 (HR, 95% CI) | Per 1 SD (HR,95%CI) | P for trend |
| --- | --- | --- | --- | --- | --- |
| **Panel A: Food intake** | | | | | |
| Cases | 301 | 345 | 362 | 1,008 |  |
| UPF intake (% g/day) | Reference | 1.10 (0.93-1.31) | 1.23 (1.08-1.40) | 1.19 (1.04-1.38) | 0.021 |
| Cases | 366 | 331 | 311 | 1,008 |  |
| MPF intake (% g/day) | Reference | 0.95 (0.81-1.12) | 0.81 (0.69-0.95) | 0.87 (0.68-0.98) | 0.033 |
| **Panel B: metabolic signature** | | | | | |
| Cases | 307 | 338 | 363 | 1,008 |  |
| UPF signature | Reference | 1.11 (0.93-1.32) | 1.24 (1.05-1.47) | 1.18 (1.03-1.29) | 0.023 |
| Cases | 372 | 329 | 307 | 1,008 |  |
| MPF signature | Reference | 0.94 (0.81-1.08) | 0.79 (0.69-0.90) | 0.83 (0.71- 0.97) | 0.041 |

Abbreviations: CVD, Cardiovascular diseases; HR, Hazard ratio; CI, Confidence interval, UPF, Ultra-processed food; MPF, minimally processed food.

Models were adjusted for age at baseline, sex, ethnicity, education, employment grade, marital status, physical activity, alcohol consumption, smoking status, family history of CVD/T2D, total energy intake, diet quality indicators (total sugar, sodium, total fat, fibre) and clinical measures.

Table S16.Two-year lag sensitivity analysis: associations of UPF intake, MPF intake, and metabolomic signature scores with incident T2D in the Whitehall II cohort (N=5,970; T2D events=832)

| Exposure | Tertile 1 (Ref) | Tertile 2 (HR, 95% CI) | Tertile 3 (HR, 95% CI) | Per 1 SD (HR,95%CI) | P for trend |
| --- | --- | --- | --- | --- | --- |
| **Panel A: Food intake** | | | | | |
| Cases | 248 | 271 | 313 | 832 |  |
| UPF intake (% g/day) | Reference | 1.13 (0.98-1.31) | 1.19 (1.02-1.39) | 1.17 (1.03-1.35) | 0.014 |
| Cases | 303 | 261 | 268 | 832 |  |
| MPF intake (% g/day) | Reference | 0.97 (0.81-1.15) | 0.80 (0.83-1.02) | 0.85 (0.78-0.94) | 0.038 |
| **Panel B: metabolic signature** | | | | |  |
| Cases | 237 | 268 | 327 | 832 |  |
| UPF signature | Reference | 1.16 (0.97-1.33) | 1.27 (1.05-1.47) | 1.21 (1.07-1.39) | 0.011 |
| Cases | 298 | 277 | 307 | 832 |  |
| MPF signature | Reference | 0.82 (0.73-1.08) | 0.58 (0.52-0.69) | 0.68 (0.57-0.88) | 4.63×10^-8^ |

Abbreviations: T2D, Type 2 diabetes; HR, Hazard ratio; CI, Confidence interval, UPF, Ultra-processed food; MPF, minimally processed food.

Models were adjusted for age at baseline, sex, ethnicity, education, employment grade, marital status, physical activity, alcohol consumption, smoking status, family history of CVD/T2D, total energy intake, diet quality indicators (total sugar, sodium, total fat, fibre) and clinical measures.

Table S17. Sensitivity analysis: associations of UPF intake, MPF intake, and metabolomic signature scores with incident T2D and CVD in the Whitehall II cohort, additionally adjusted for AHEI-2010 in the Whitehall II cohort (N=6,010)

|  | T2D | | | | | CVD | | | | |
| --- | --- | --- | --- | --- | --- | --- | --- | --- | --- | --- |
|  | Tertile 1 (Ref) | Tertile 2 (HR, 95% CI) | Tertile 3 (HR, 95% CI) | Per 1 SD (HR, 95% CI) | P for trend | Tertile 1 (Ref) | Tertile 2 (HR, 95% CI) | Tertile 3 (HR, 95% CI) | Per 1 SD (HR, 95% CI) | P for trend |
| **Panel A: Food intake** | | | | | | | | | | |
| UPF intake | Reference | 1.13 (0.98-1.31) | 1.19 (1.04-1.39) | 1.15 (1.03-1.31) | 0.014 | Reference | 1.13 (0.97-1.28) | 1.23 (1.06-1.42) | 1.17 (1.03-1.33) | 0.028 |
| MPF intake | Reference | 0.97 (0.81-1.15) | 0.85 (0.76-0.91) | 0.87 (0.78-0.97) | 0.041 | Reference | 0.96 (0.82-1.13) | 0.75 (0.63-0.89) | 0.83 (0.77-0.94) | 0.031 |
| **Panel B: Metabolomic signature** | | | | | | | | | | |
| UPF signature | Reference | 1.11 (0.93-1.32) | 1.24 (1.07-1.47) | 1.18 (1.05-1.32) | 0.011 | Reference | 1.13 (0.95-1.28) | 1.25 (1.07-1.37) | 1.20 (1.06-1.33) | 0.013 |
| MPF signature | Reference | 0.82 (0.77-1.08) | 0.58 (0.48-0.69) | 0.67 (0.59-0.76) | 4.73×10⁻⁸ | Reference | 0.93 (0.86-1.05) | 0.84 (0.71-0.96) | 0.87 (0.73-0.95) | 0.037 |

Abbreviations: CVD, Cardiovascular diseases; T2D, Type 2 diabetes; HR, Hazard ratio; CI, Confidence interval, UPF, Ultra-processed food; MPF, minimally processed food.

Models were adjusted for age at baseline, sex, ethnicity, education, employment grade, marital status, physical activity, alcohol consumption, smoking status, family history of CVD/T2D, total energy intake, AHEI-2010 and clinical measures.

Table S18. Nova classification sensitivity analysis using lower and upper bound assumptions for reclassification

| **Ambiguous items** | | | | | |
| --- | --- | --- | --- | --- | --- |
| **Food Category** | **FFQ Item** | **Reference (Main Analysis)** | **Upper Bound (Classify Up)** | **Lower Bound (Classify Down)** | **Rationale for Ambiguity** |
| Bread | Average brown bread/rolls | UPF | UPF | PF | Mass-produced bread (emulsifiers, preservatives) = UPF; traditional bakery bread (flour, water, salt, yeast) = PF. FFQ cannot distinguish. Most UK bread is mass-produced, supporting UPF as reference. |
| Bread | Average white bread/rolls | UPF | UPF | PF | Same rationale as brown bread. White bread particularly likely to be mass-produced in UK context. |
| Bread | Average wholemeal bread/rolls | UPF | UPF | PF | Same rationale. Wholemeal bread from bakeries using only flour, water, salt, yeast = PF. Industrial versions contain additives = UPF. |
| Bread | Average crispbread | UPF | UPF | PF | Simple crispbreads (e.g. Ryvita: rye flour, salt) = PF. Many commercial variants contain added fat, sugar, flavourings = UPF. |
| Cereals | Average Shredded Wheat, Weetabix | UPF | UPF | MPF | Shredded Wheat = 100% whole wheat, no additives → MPF. Weetabix = whole wheat + sugar + salt + barley malt extract → borderline. Grouped item creates ambiguity. |
| Cereals | Average Muesli, Fruit & Fibre | UPF | UPF | PF | Simple muesli (oats, nuts, dried fruit, no added sugar) = MPF. Commercial muesli often has added sugar/honey/oil = PF. Fruit & Fibre contains sugar coating/flavourings = UPF. Grouped item. |
| Cereals | Average porridge, Readybrek | MPF | UPF | MPF | Plain porridge oats = MPF. Readybrek is instant, fortified, industrially processed → could be UPF. Grouping creates ambiguity. Most UK porridge consumption is plain oats. |
| Dairy | Average yoghurt | MPF | UPF | MPF | Plain yoghurt (milk + bacterial cultures) = MPF. Flavoured/fruit yoghurt with added sugar, thickeners, flavourings = UPF. FFQ does not distinguish. UK market dominated by flavoured varieties. |
| Fish | Average oily fish - fresh/canned | MPF | PF | MPF | Fresh oily fish = MPF. Canned fish (preserved in oil/brine with salt) = PF. Grouping fresh and canned together creates ambiguity. |
| Drink | Average spirits | UPF | UPF | PF | Distilled spirits involve industrial processing but basic spirits (vodka, whisky) may not contain UPF-defining additives. Some researchers classify as PF (fermented + distilled). Flavoured spirits = UPF. |
| Drink | Average port, sherry, vermouth | UPF | UPF | PF | Port/sherry = fortified wines, could be PF (wine + spirit). Vermouth contains added botanicals, sugar, flavourings → UPF. Grouped item creates ambiguity. |
| Drink | Average liqueurs | UPF | UPF | PF | Most liqueurs contain added sugar, flavourings, colourings = UPF. However, some traditional liqueurs have simple ingredient lists → PF. Majority are UPF. |
| Fat | Average peanut butter | PF | UPF | PF | Most commercial peanut butter contains added sugar, palm oil, emulsifiers = UPF. Simple peanut butter (peanuts + salt) = PF. UK market predominantly sells versions with additives. |
| Meat | Average beef burgers | UPF | UPF | PF | Mass-produced burgers contain additives, fillers, flavourings = UPF. Butcher's/homemade burgers (minced beef + salt) = PF. FFQ cannot distinguish. |
| Meat | Average liver, liver pâté | UPF | UPF | PF | Liver alone = MPF. Liver pâté (industrial, with additives, preservatives) = UPF. Grouping creates ambiguity. If interpreted as mainly pâté consumption = UPF. |
| Meat | Average sausages | UPF | UPF | PF | Mass-produced sausages contain phosphates, nitrites, fillers = UPF. Traditional butcher's sausages (meat, breadcrumbs, salt, herbs) = PF. UK market mostly mass-produced. |
| Meat | Average meat soup | UPF | UPF | PF | Commercial/tinned soup = UPF (additives, flavour enhancers). Homemade soup = culinary preparation of MPF. FFQ cannot distinguish preparation method. |
| Pasta | Average lasagne | UPF | UPF | PF | Ready-made/frozen lasagne = UPF. Homemade lasagne = culinary preparation. If commercial dominates = UPF. Could be PF if interpreted as simple cheese/meat/pasta dish. |
| Pasta | Average pizza | UPF | UPF | PF | Commercial/frozen/takeaway pizza = UPF. Simple homemade or traditional pizza = PF. UK consumption predominantly commercial. |
| Pasta | Average quiche | UPF | UPF | PF | Commercial/shop-bought quiche = UPF. Homemade quiche = PF. Similar ambiguity to lasagne and pizza. |
| Potato | Average chips or French fries | UPF | UPF | MPF | Frozen/fast-food chips with coatings, additives = UPF. Homemade chips (potato + oil) = MPF + PCI culinary preparation. FFQ cannot distinguish. |
| Potato | Average potato salad | PF | UPF | PF | Commercial potato salad with emulsifiers, preservatives = UPF. Simple potato salad (potato, mayonnaise, herbs) = PF. |
| Sauces | Average french dressing, vinaigrette | UPF | UPF | PCI | Commercial dressings contain emulsifiers, thickeners, flavourings = UPF. Homemade vinaigrette (oil + vinegar + mustard) = PCI. FFQ cannot distinguish. |
| Sauces | Average pickles, chutney | UPF | UPF | PF | Commercial pickles with colourings, preservatives = UPF. Traditional pickles/chutney (vinegar, sugar, salt, spices) = PF. |
| Sauces | Average sauces - white, gravy | UPF | UPF | PCI | Packet/instant gravy and white sauce = UPF. Homemade gravy (drippings + flour) or white sauce (butter, flour, milk) = PCI. FFQ cannot distinguish. |
| Sauces | Average Marmite, Bovril | UPF | UPF | PF | Industrially produced from yeast/meat extract. Contains added vitamins, flavourings. Could be argued as PF (simple concentrated extract). Most researchers classify as UPF. |
| Sweets/snacks | Average jam, marmalade, honey | UPF | UPF | PF | Honey = MPF (unprocessed). Traditional jam/marmalade (fruit + sugar, cooked) = PF. Commercial versions may contain pectin, preservatives = UPF. Grouping honey with jam is problematic. High-impact item. |
| Sweets/snacks | Average ice cream, choc ices | UPF | UPF | PF | Mass-produced ice cream with emulsifiers, stabilisers, flavourings = UPF. Traditional ice cream (cream, sugar, eggs) = PF. Choc ices are clearly UPF. |
| Sweets/snacks | Average milk puddings | UPF | UPF | PF | Commercial/instant milk puddings = UPF. Homemade rice pudding or custard (milk, sugar, eggs) = PF. |
| Sweets/snacks | Average fruit pies, tarts, crumble | UPF | UPF | PF | Shop-bought versions with additives = UPF. Homemade (flour, butter, sugar, fruit) = PF/culinary preparation. |
| Vegetable | Average baked beans | UPF | UPF | PF | Classic ambiguous UK item. Standard baked beans (beans, tomato sauce, sugar, salt) have simple ingredients → PF. Some brands contain modified starch, flavourings → UPF. Heinz (market leader) has relatively simple recipe. |
| Vegetable | Average coleslaw | UPF | UPF | PF | Commercial coleslaw with emulsifiers, preservatives = UPF. Homemade coleslaw (cabbage, carrot, mayonnaise) = PF. |
| Vegetable | Average vegetable soup | UPF | UPF | PF | Commercial/tinned soup = UPF. Homemade vegetable soup = culinary preparation of MPF. Same rationale as meat soup. |
| Vegetable | Average marinated tofu | UPF | UPF | PF | Plain tofu (soybeans, water, coagulant) = MPF. Marinated versions with added flavourings = PF or UPF depending on additives. |
| Vegetable | Average tinned fruit | PF | UPF | PF | Tinned fruit in juice = PF. Tinned fruit in heavy syrup with added preservatives/colourings could be UPF. Most UK tinned fruit has simple ingredients. |

Abbreviations: UPF, ultraprocessed foods; PF, processed foods; MPF, minimally processed foods. PCI, processed culinary ingredients.

Table S19**.** Nova reclassification sensitivity analysis: associations between UPF/MPF intake and risk of CVD and T2D in the Whitehall II cohort (N=6,010)

|  |  | **Reference** | **Upper bound** | **Lower bound** |
| --- | --- | --- | --- | --- |
| **Panel A.** Median intake and Spearman rank correlations across Nova classification scenarios | | | | |
| UPF intake (%g/day) | | | | |
|  | Median (IQR) | 18.3 (14.0–23.5) | 20.0 (15.7–25.5) | 15.1 (9.8–19.2) |
| Spearman ρ | |  |  |  |
|  | Reference | 1.00 | 0.92 | 0.93 |
|  | Upper bound | 0.88 | 1.00 | 0.89 |
|  | Lower bound | 0.89 | 0.93 | 1.00 |
| MPF intake (%g/day) | | | | |
|  | Median (IQR) | 62.1 (54.0–69.3) | 60.1 (51.8–67.2) | 67.8 (53.4–68.2) |
| Spearman ρ | |  |  |  |
|  | Reference | 1.00 | 0.88 | 0.90 |
|  | Upper bound | 0.91 | 1.00 | 0.89 |
|  | Lower bound | 0.87 | 0.90 | 1.00 |
| **Panel B.** Associations with cardiovascular disease (CVD) | | | | |
| UPF intake | | | | |
|  | Per 1-SD increase | 1.17 (1.03–1.35) | 1.18 (1.03–1.35) | 1.16 (1.01–1.32) |
| MPF intake | | | | |
|  | Per 1-SD increase | 0.87 (0.79–0.93) | 0.85 (0.79–0.95) | 0.87 (0.72–0.97) |
| **Panel C.** Associations with type 2 diabetes (T2D) | | | | |
| UPF intake | | | | |
|  | Per 1-SD increase | 1.15 (1.02–1.28) | 1.17 (1.08–1.26) | 1.12 (1.01–1.20) |
| MPF intake | | | | |
|  | Per 1-SD increase | 0.86 (0.75–0.94) | 0.84 (0.72–0.93) | 0.88 (0.73–0.97) |

Abbreviations: CI, confidence interval; CVD, cardiovascular disease; HR, hazard ratio; IQR, interquartile range; MPF, minimally processed food; SD, standard deviation; T2D, type 2 diabetes; UPF, ultra-processed food.

Panel A: Intakes are expressed as percentage of total food weight (g/day). Spearman correlation coefficients (ρ) quantify agreement between participant rankings under each Nova classification scenario. The reference scenario uses the primary Nova classification. Upper and lower bound scenarios reclassify 35 ambiguous FFQ items to maximise or minimise UPF/MPF intake, respectively.

Panels B and C: Values are hazard ratios (95% CI) from Cox proportional hazards models per 1-SD increase in intake. Models adjusted for age at baseline, sex, ethnicity, education, employment grade, marital status, physical activity, alcohol consumption, smoking status, family history of CVD/T2D, total energy intake, BMI, hypertension, dyslipidaemia, diet quality indicators (total sugar, sodium, total fat, fibre), and clinical measures.

Table S20. Associations of UPF and MPF metabolomic signature scores with incident CVD and T2D after additional adjustment for dietary UPF or MPF intake

| **Exposure** | **Outcome** | **Main fully adjusted model**  **HR (95% CI)** | **Additional adjustment for**  **UPF/MPF intake HR (95% CI)** |
| --- | --- | --- | --- |
| UPF signature | CVD | 1.14 (1.02–1.31) | 1.15 (1.03–1.35) |
| MPF signature | CVD | 0.86 (0.80–0.95) | 0.88 (0.75–0.96) |
| UPF signature | T2D | 1.18 (1.04–1.36) | 1.17 (1.03–1.29) |
| MPF signature | T2D | 0.72 (0.62–0.84) | 0.75 (0.66–0.87) |

Abbreviations: HR, hazard ratio; CI, confidence interval; CVD, cardiovascular disease; T2D, type 2 diabetes; UPF, ultra-processed food; MPF, minimally processed food.

Models were adjusted for age at baseline, sex, ethnicity, education, employment grade, marital status, physical activity, alcohol consumption, smoking status, family history of CVD/T2D, total energy intake, diet quality indicators (total sugar, sodium, total fat, fibre) and clinical measures, with additional adjustment for UPF intake (for UPF signature models) or MPF intake (for MPF signature models)

Table S21. Sensitivity analysis: UPF and MPF metabolomic signatures re-derived with confounders entered as unpenalised terms in the elastic net

| **Metric** | **UPF (adjusted)** | **MPF (adjusted)** |
| --- | --- | --- |
| **Signature composition** | | |
| Correlation of signature scores (vs primary) | 0.98 | 0.97 |
| **Diet–outcome association** | | |
| Incident CVD, HR (95% CI) per SD | 1.17 (1.04-1.37) | 0.85 (0.78-0.93) |
| Incident T2D, HR (95% CI) per SD | 1.18 (1.03-1.30) | 0.83 (0.77-0.90) |
| **Signature–outcome association** | | |
| Incident CVD, HR (95% CI) per SD | 1.16 (1.03-1.35) | 0.82 (0.72-0.91) |
| Incident T2D, HR (95% CI) per SD | 1.20 (1.06-1.40) | 0.73 (0.66-0.85) |

Abbreviations: BMI, body mass index; CI, confidence interval; HR, hazard ratio; MPF, minimally processed foods; SD, standard deviation; UPF, ultra-processed foods; CVD, cardiovascular diseases. T2D, type 2 diabetes.

Note: the sensitivity metabolomic signature was re-derived with confounders entered as unpenalised terms (penalty factor = 0), so that metabolite selection was conditional on them. The diet–outcome and signature–outcome Cox models were adjusted for the same covariates as the main analysis (Model 2). Per 1-SD estimates represent the HR associated with each standard deviation increment in the exposure or signature score, modelled as a continuous variable.

Table S22. Sensitivity analysis for dietary exposure timing: intake consistency and phase 5–only metabolomic signatures

Panel A. Consistency of dietary intake between phase 3 and phase 5

| **Dietary exposure** | **Phase 3 median (IQR)** | **Phase 5 median (IQR)** | **Pearson r^a^** |
| --- | --- | --- | --- |
| UPF intake (% g/day) | 19.0 (14.1–24.9) | 17.9 (12.9–23.7) | 0.73 |
| MPF intake (% g/day) | 62.0 (52.8–69.8) | 62.4 (54.0–70.3) | 0.79 |

^a^r, Pearson’s correlation between phase 3 and phase 5 intake. Phases 3 (1991–94) and 5 (1997–99) were approximately 5 years apart.

Panel B. Phase 5–only UPF and MPF metabolomic signatures and their associations with incident CVD and T2D

| **Metric** | **UPF (phase 5)** | **MPF (phase 5)** |
| --- | --- | --- |
| **Signature composition** | | |
| Pearson’s correlation of signature scores (vs primary) | 0.94 | 0.91 |
| **Diet-outcome association** |  |  |
| Incident CVD, HR (95% CI) per SD | 1.18 (1.03–1.38) | 0.85 (0.77–0.94) |
| Incident T2D, HR (95% CI) per SD | 1.17 (1.02–1.36) | 0.83 (0.76–0.91) |
| **Signature-outcome association** |  |  |
| Incident CVD, HR (95% CI) per SD | 1.22 (1.07–1.41) | 0.82 (0.71–0.92) |
| Incident T2D, HR (95% CI) per SD | 1.20 (1.05–1.39) | 0.74 (0.65–0.86) |

Abbreviations: BMI, body mass index; CI, confidence interval; HR, hazard ratio; MPF, minimally processed foods; SD, standard deviation; UPF, ultra-processed foods; CVD, cardiovascular diseases. T2D, type 2 diabetes.

Note: The primary signature was derived from the averaged phase 3 + phase 5 exposure; the sensitivity signature was re-derived using phase 5 UPF/ MPF intake. All models used the same fully adjusted covariate set as the main analysis (Model 2). Per 1-SD estimates represent the HR associated with each standard deviation increment in the exposure, modelled as a continuous variable.

Table S23. Comparison of discriminative performance (Harrell’s C-index) for incident CVD and T2D across models

| Model | CVD C-index (95% CI) | T2D C-index (95 % CI) |
| --- | --- | --- |
| Covariates only | 0.642 (0.612, 0.672) | 0.735 (0.701, 0.769) |
| Covariates +UPF intake | 0.644 (0.614, 0.674) | 0.738 (0.704, 0.772) |
| Covariates + UPF metabolomic signature | 0.651 (0.621, 0.681) | 0.753 (0.720, 0.786) |
| Covariates + UPF intake + UPF metabolomic signature | 0.652 (0.622, 0.682) | 0.754 (0.721, 0.787) |
| Covariates +MPF intake | 0.643 (0.613, 0.673) | 0.737 (0.703, 0.771) |
| Covariates + MPF metabolomic signature | 0.654 (0.624, 0.684) | 0.756 (0.723, 0.789) |
| Covariates + MPF intake +MPF metabolomic signature | 0.655 (0.625, 0.685) | 0.757 (0.724, 0.790) |

Abbreviations: CVD, cardiovascular disease; T2D, type 2 diabetes; UPF, ultra-processed food; MPF, minimally processed food; CI, confidence interval.

Covariates included age at baseline, sex, ethnicity, education, employment grade, marital status, physical activity, alcohol consumption, smoking status, family history of CVD/T2D, total energy intake, diet quality indicators (total sugar, sodium, total fat, fibre) and clinical measures.

Table S24. Pathway-specific estimates from causal mediation analyses: exposure–mediator and mediator–outcome associations for UPF and MPF intake with incident CVD and T2D in the Whitehall II cohort (N = 6,010).

| Outcome | Exposure | Mediator | Exposure–Mediator pathway | | Mediator–Outcome pathway | |
| --- | --- | --- | --- | --- | --- | --- |
|  |  |  | **β** | **95% CI** | **HR** | **95% CI** |
| CVD | UPF intake | UPF metabolomic signature | 0.33 | (0.28, 0.38) | 1.19 | (1.05, 1.32) |
|  |  | Sugar intake | 0.30 | (0.25, 0.35) | 1.10 | (0.96, 1.26) |
|  |  | Sodium intake | 0.25 | (0.20, 0.30) | 0.93 | (0.81, 1.07) |
|  |  | Saturated fat intake | 0.28 | (0.23, 0.33) | 1.11 | (0.97, 1.27) |
|  | MPF intake | MPF metabolomic signature | 0.32 | (0.27, 0.37) | 0.84 | (0.75, 0.93) |
|  |  | Sugar intake | −0.20 | (−0.25, −0.15) | 1.05 | (0.92, 1.20) |
|  |  | Sodium intake | −0.20 | (−0.25, −0.15) | 1.10 | (0.96, 1.27) |
|  |  | Saturated fat intake | −0.22 | (−0.27, −0.17) | 1.09 | (0.95, 1.25) |
| T2D | UPF intake | UPF metabolomic signature | 0.33 | (0.28, 0.38) | 1.18 | (1.03, 1.37) |
|  |  | Sugar intake | 0.30 | (0.25, 0.35) | 1.18 | (1.03, 1.35) |
|  |  | Sodium intake | 0.25 | (0.20, 0.30) | 1.12 | (0.97, 1.30) |
|  |  | Saturated fat intake | 0.28 | (0.23, 0.33) | 1.15 | (0.99, 1.33) |
|  | MPF intake | MPF metabolomic signature | 0.32 | (0.27, 0.37) | 0.74 | (0.69, 0.86) |
|  |  | Sugar intake | −0.20 | (−0.25, −0.15) | 1.10 | (0.96, 1.26) |
|  |  | Sodium intake | −0.20 | (−0.25, −0.15) | 0.90 | (0.78, 1.05) |
|  |  | Saturated fat intake | −0.22 | (−0.27, −0.17) | 1.15 | (0.99, 1.33) |

Abbreviations: CI, confidence interval; CVD, cardiovascular disease; HR, hazard ratio; MPF, minimally processed foods; T2D, type 2 diabetes; UPF, ultra-processed foods.

The exposure–mediator pathway was estimated using multivariable linear regression (β coefficient per 1-SD increase in z-scored exposure). The mediator–outcome pathway was estimated using parametric survival models (HR per 1-SD increase in the mediator, adjusted for the exposure). Both models were adjusted for covariates as specified in Model 1 (age, sex, ethnicity, education, employment grade, marital status, physical activity, alcohol consumption, smoking status, family history of CVD, total energy intake, BMI, hypertension, and dyslipidaemia). Dietary quality indicators were excluded as potential causal pathway variables.
